# Metagenomic analysis of an urban resistome before and after wastewater treatment

**DOI:** 10.1038/s41598-020-65031-y

**Published:** 2020-05-18

**Authors:** Felipe Lira, Ivone Vaz-Moreira, Javier Tamames, Célia M. Manaia, José Luis Martínez

**Affiliations:** 1 Centro Nacional de Biotecnología. Consejo Superior de Investigaciones Científicas (CSIC), Cantoblanco, Madrid USA; 2 Universidade Católica Portuguesa. CBQF - Centro de Biotecnologia e Química Fina – Laboratório Associado. Escola Superior de Biotecnologia, Rua Diogo Botelho 1327, 4169-005 Porto, Portugal

**Keywords:** Metagenomics, Environmental impact

## Abstract

Determining the effect of wastewater treatment in water resistome is a topic of interest for water quality, mainly under re-use and One-Health perspectives. The resistome, the plasmidome, and the bacterial community composition of samples from influents and treated effluents from a wastewater treatment plant located in Northern Portugal were studied using metagenomic techniques. Wastewater treatment contributed to reduce the abundance of resistance genes and of plasmid replicons, coinciding with a decline in the number of intrinsic resistance genes from *Enterobacteriaceae*, as well as with a reduction in the relative abundance of *Firmicutes* and *Proteobacteria* after treatment. These taxons comprise bacterial pathogens, including those belonging to the ESKAPE group, which encompasses bacteria with the highest risk of acquiring antibiotic resistance, being the most relevant hosts of resistance genes acquired through horizontal gene transfer. Our results support that wastewater treatment efficiently removes the hosts of antibiotic resistance genes and, consequently, the harboured antibiotic resistance genes. Principal component analysis indicates that the resistome and the bacterial composition clustered together in influent samples, while did not cluster in final effluent samples. Our results suggest that wastewater treatment mitigates the environmental dissemination of urban resistome, through the removal of the hosts harbouring mobile resistance genes.

## Introduction

Most studies on antibiotic resistance have concentrated their efforts on human pathogens and in human-linked environments (hospitals, human hosts,…). However, the full understanding of the origin, evolution, spread, and maintenance of antibiotic resistance (AR) requires an integrative approach in which all ecosystems that may contribute to such evolution are studied; a One Health approach^1,2^. One Heath is a concept derived from the idea proposed by Rudolph Virchow in the 19th Century, stating that “between animal and human medicine there are no dividing lines- nor should there be”. The idea behind is that, at least in the case of infectious diseases, different ecosystems, not just the human host, may contribute to the spread of the infective agents. This is particularly relevant in the case of AR, since it is generally accepted that antibiotic resistance genes (ARGs), currently present in human pathogens, are originated in environmental organisms and, therefore, natural ecosystems can be reservoirs and participate in the spread of AR^3–9^. Consequently, it has been proposed that AR is the quintessential One Health issue^2^, and that studies, integrating all habitats potentially involved in the evolution and spread of AR are needed^1,10,11^. Indeed, seminal work showed that animal farms could be a reservoir of AR^12^. As a consequence, the use of antibiotics in farming, for non-therapeutic or prophylactic procedures (as animal fattening), has been banned in different countries^13,14^. More recently, non-clinical environments (natural and man-made ecosystems) have been proposed to play a major role in the dissemination of AR^9,15,16^. In particular man-made environments such as wastewater treatment plants (WWTPs) have received special attention^17–19^. Within these ecosystems bacterial pathogens (several of them already carrying acquired ARGs), released within stools, coexist with different types of pollutants, including antibiotics and other selectors of AR^20^. This might make these environments highly relevant for the spread of AR. In allocations in which water is not usually treated, antibiotic resistant bacteria (ARB) can easily disseminate (for example through re-used water). Indeed, water consumption was shown to be one of the major routes for the dissemination of relevant ARGs such as *bla*_*NDM-1*_^21,22^. Nevertheless, water dissemination of ARB has also been reported in places where water is regularly treated^23,24^.

Further knowledge about the influence of WWT on AR abundance is hence of relevance because, since despite their unquestionable role on the environmental and human-health protection, WWTPs are leaky barriers that might influence the dissemination of ARGs. An expected common outcome of WWT is the overall reduction in the number of microorganisms, that should correlate with a reduction in the number of ARGs^25^. However, results on the effect of WWT on the prevalence of specific ARGs are sometimes contradictory. While it is recognized that WWT reduces the amount and diversity of ARGs, and hence reduces the chances of AR dissemination^26,27^, some studies state that WWTPs are non-negligible sources of ARB^28,29^, contributing to the spread of ARGs^26,30,31^. Further, it has been recently suggested that treatment may even enhance, at least on occasions, the prevalence of ARBs and ARGs in wastewater^32^.

Most studies in this topic rely on the quantification of a set of sentinel ARGs, which are used as markers of the overall ARGs in the samples. However, these analyses are limited to the tested genes and, in addition, hinders the comparative meta-analysis of published studies. Besides these potential technical limitations, different results from different studies can also be the consequence of sampling, of the different types of WWT or of gene drift. Gene drift is part of the whole evolution process, which increases stochasticity in evolution, and appears when the microbial population suffers strong bottlenecks^33,34^. In this situation, the individuals that surpass the bottleneck are not necessarily the fittest (although they must be fit enough to be kept in the population), but just the ones that remain. Therefore, variations in the bacterial community after being submitted to a given type of bottleneck-driving stress, for example WWT, can present some differences every time the bottleneck is applied. In the present work, we have studied, using metagenomic techniques, a set of samples from the influent and final effluent of a WWTP located in Northern Portugal. From our study, we have been able to determine that ARGs and bacterial community composition of wastewater is similar in the analysed raw wastewater samples. In line with previous publications, this may suggest the existence of urban microbiomes/resistomes^35,36^ that reflect the overall composition of the global microbiome and resistome of the population served by the corresponding WWTP and hence could be used for continuous global surveillance of AR^35^. We have also been able to determine that treatment specifically eliminates ARGs, likely by removing the hosts harbouring them.

## Results

The resistome of three samples of the influent (RAW) and of the final effluent, after secondary treatment and UV disinfection (UV), which were collected in three distinct months from a WWTP in Northern Portugal, was analysed. The prevalence of a limited set of selected ARGs has been analysed previously by real-time PCR in the same samples^37^.

### Antibiotic resistance genes

Metagenomics sequences were obtained from all samples and the CARD database^38^ was used to screen the presence and relative abundance of ARGs. From the analysis, 259 ARGs included in the database were detected in the metagenomes. The sequences ARGs that currently contribute to AR in bacterial pathogens are highly conserved^39^, and predicting resistance by means of sequence homology can provide false positives^18^. Because of this, we used in our analysis stringent criteria for the identification of ARGs (95% of coverage of the reads length and 95% of identity of the reads length). It is important to highlight that the CARD database includes not only mobile ARGs, but also genes whose mutation lead to antimicrobial resistance. The results from the metagenomics annotation was hence manually curated to remove genes that contribute to resistance just upon their mutation. The resulting 235 ARGs were divided in two categories that were analyzed independently. One category os formed by those genes that have been reported to be present in mobile genetic elements (MGEs). The second one is formed by genes involved in intrinsic resistance that, as been previously discussed^18,39^ are phylogenetic markers more than a risk for the dissemination of resistance. For comparison purposes, the number of ARGs reads in each sample was normalized to ten millions of total reads in this sample.

**Figure 1:**
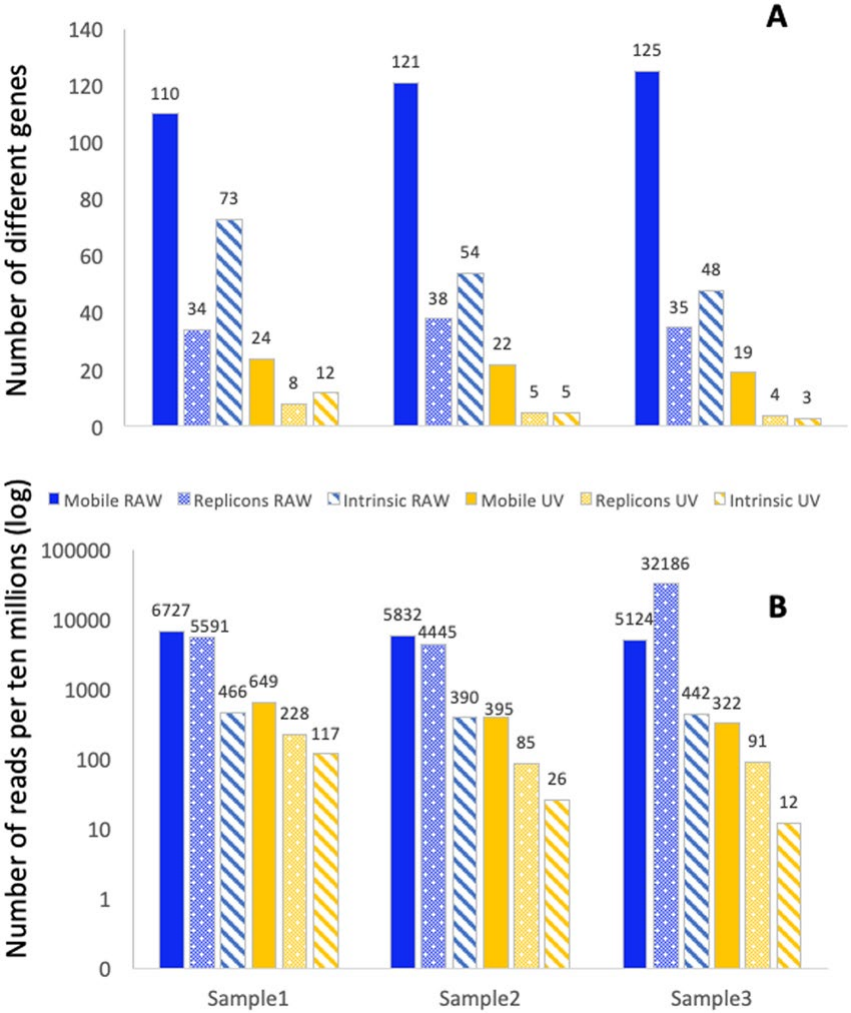
Number of reads and number of different ARGs known to be acquired by bacterial pathogens, plasmids replicons, and intrinsic resistance genes. Panel A shows the number of different ARGs, plasmids replicons and intrinsic resistance genes. Panel B shows the reads corresponding to each of the categories per ten million reads. Data in panel B are presented in a logarithmic scale. Blue: RAW wastewater, yellow: treated wastewater (UV). Solid colour: mobile ARGs, dotted: plasmid replicons, striped: intrinsic resistance genes.

A total of 163 different mobile ARGs were found distributed through all samples. Among them, RAW water presented a total of 162 ARGs (corresponding to an average of 155.7 ± 4.5 per sample), recruiting 6132.39 ± 341.84 reads per ten millions in each sample on average. In comparison, the final UV samples presented a total of 39 ARGs (21.7 ± 2.05 in each sample on average) recruiting an average of 253.52 ± 93.30 reads per ten millions of total reads in each sample. The differences between RAW and UV samples regarding both the number of different ARGs and the total number of ARGs’ reads were statistically significant (p < 0.001), a result clearly showing that WWT specifically decreased the relative abundance (respecting the total number of reads) of those mobile ARGs that have been described to be associated with human pathogens (Fig. 1, Table S1).

**Figure 2:**
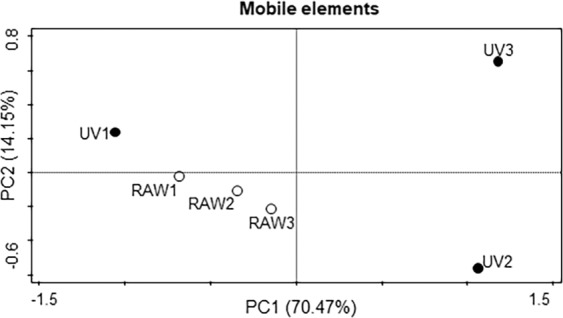
Principal Component Analysis (PCA) of ARGs known to be present in mobile elements in bacterial pathogens, in the different samples of raw wastewater and UV-treated wastewater samples. White dots represent the raw wastewater samples (RAW1, RAW2, RAW3), and the black dots represent the UV-treated wastewater samples (UV1, UV2, UV3).

Once a global trend was found, we focused on the specific pattern of each sample. For this purpose, we performed a principal component analysis (PCA) of the data obtained. As shown in Fig. 2. RAW wastewater samples grouped together, while the samples from final effluent presented a more dispersed distribution. Besides, it was observed that the ARGs that were consistently detected in the final UV samples were only 6, out of the 41 detected in the different sampling dates: *msrE*, *mphE, sul1*, *APH(3”)-Ib*, *BEL-1*, and *aadA11* (Table S1).

### Plasmid replicons

**Figure 3:**
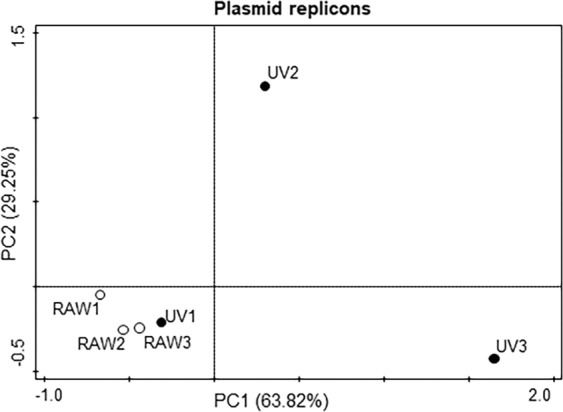
Principal Component Analysis (PCA) of plasmid replicons in the different samples of raw wastewater and UV-treated wastewater samples. White dots represent the raw wastewater samples (RAW1, RAW2, RAW3), and the black dots represent the UV-treated wastewater samples (UV1, UV2, UV3).

Once we have analysed the effect of the WWT on the mobile resistome, we studied the effects of water treatment on the relative abundance of MGEs, which can be involved in the dissemination of resistance. To that goal, since plasmids have a major role in the spread of ARGs, their replication origins were used as markers to determine the abundance of these mobile elements. After normalizing the number of reads to ten millions of reads in each sample, 59 replicon types, with an average of 35.67 ± 15.43 different replicons on each sample and recruiting in each sample an average of 14073.23 ± 12815.14 reads per million of total reads, were found in the RAW wastewater samples. These values decreased down significantly (p < 0.001) to 10 replicon types, with an average of 5.67 ± 1,70 replicons per sample and 134.73 ± 66.13 reads per ten millions of reads in final effluent samples (Table S2, Fig. 1). The most abundant replicons were repUS2, IncP(6), and IncQ2, in this order. RepUS2 plasmids are narrow-range plasmids, present in Gram-positive bacteria, and have been recently found to be prevalent in gut microbiome^40^, while IncP(6) and IncQ2 are broad-range conjugative plasmids that contribute to the acquisition of multidrug resistance in a variety of Gram-negative bacteria^41–45^. (Fig. 1, Table S2). In agreement with the results obtained with ARGs data, PCA showed that influent samples grouped together in comparison with treated wastewater samples with a spreader distribution (Fig. 3). In this case, just one plasmid replicon was detected in the three sampling dates in the final UV, the IncP(Beta) replicon type (Table S2).

### Intrinsic antibiotic resistance genes

The intrinsic resistome has been defined as the ensemble of genes that contribute to the characteristic phenotype of antibiotic resistance of a given bacterial species^46^. Differing to the mobile resistome, which has been recently acquired (in evolutionary terms) and is transmitted via horizontal gene transfer among different bacteria^47–49^, the intrinsic resistome is universally encoded within the genome of a bacterial species and is neither dependent on mutations nor due to horizontal gene transfer^50,51^. It has been then proposed that intrinsic ARGs should be considered as phylogenetic markers^18^. Given that antibiotic resistance analyses have focused mainly on bacterial pathogens, databases, including CARD, are biased in favour of genes present in such pathogens. We took then advantage of this situation and used the analysis of intrinsic resistance genes as a proxy to track the abundance of bacterial pathogens. Like in the other analysis, the number of reads corresponding to intrinsic resistance ARGs was normalized to ten millions of reads. We found that the number of reads associated with intrinsic resistance genes (Fig. 1, Table S3), is significantly lower (p < 0.001) after WWT (51.54 ± 46.57) than in the samples of RAW wastewater (432.92 ± 31.77). A similar trend was observed in the case of *Enterobacteriaceae*; RAW water samples contained an average of 212.64 ± 16.16 reads, while this number was reduced to 37.84 ± 35.96 reads in UV-treated samples (p < 0.005). Further, the number of different intrinsic ARGs (Fig. 1, Table S3) is also significantly lower (p < 0.001) in treated UV water (6.67 ± 3.85) than in RAW water (58.33 ± 10.66). When the analysis focused in *Enterobacteriaceae*, the relative of different intrinsic ARGs belonging to this family of bacteria was also significantly larger p < 0.001) in samples from RAW water (33.33 ± 1.24) than in UV treated water (5.53 ± 3.30). Together with the results of the analysis of mobile ARGs, this result suggests that *Enterobacteriaceae* bacteria harbouring ARGs are efficiently and specifically depleted upon treatment.

### Bacterial community analysis

**Figure 4:**
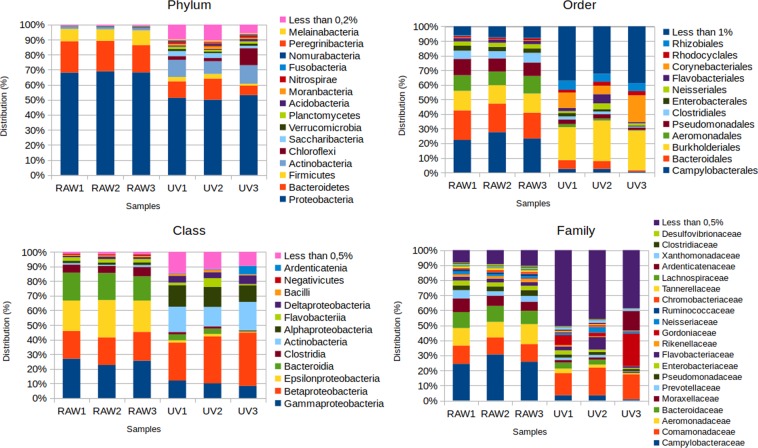
Bacterial composition of wastewater before and after treatment. The Figure shows the composition of the bacterial community at the levels of Phylum, Class, Order and Family of RAW and UV water samples.

The structure of the bacterial community in each sample was analysed by assigning the reads from each of the samples to the corresponding taxonomic group, from Phylum to Family (Supplementary material Tables S4-S7). The results are shown in Fig. 4. The main Phyla in samples from RAW water were *Proteobacteria* (68.47% ± 0.38), followed by *Bacteroidetes* (19.72% ± 1.19) and *Firmicutes* (8.48% ± 3.75). Upon treatment, the relative abundance of each of these Phyla was significantly lower (p < 0.05 in all cases), although *Proteobacteria* was still the most predominant Phylum (51.55% ± 1.17), as well as *Bacteroidetes* (10.41% ± 3,22). A shift was observed for *Actinobacteria*, which relative abundance increased from 1.21% ± 0.56 in RAW water to 10.71% ± 1.62 in UV treated water. In parallel, the relative abundance of *Firmicutes* declined to 2.43% ± 0.79 in UV treated water samples. The analysis of the bacterial community at the Class level showed that the relative abundance of members of the class *Gammaproteobacteria*, which comprises several human pathogens significantly declined (p < 0.001) upon treatment, from 25.09% ± 1,80 in RAW water to 10.15% ± 1,48 in UV-treated samples. The same variation was observed for members of the classes that contain bacteria frequently present in the human gut, as *Epsilonproteobacteria*, which prevalence shifted down from 22.61% ± 2.08 to 1.26% ± 0.59 upon wastewater treatment (p < 0.001) or Bacteroidia, showing a decrease from 18.03% ± 1.08 in RAW water to 2.79% ± 1.48 in UV-treated samples (p < 0.001). A variation in the opposite direction was observed for classes that contain mainly environmental, non-pathogenic, bacteria as *Alphaproteobacteria*, which presence after treatment increases from 1.73% ± 0.14 in RAW water to 13.25% ± 1.36 in UV water (p < 0.001) and *Actinobacteria* that recruits 1.06% ± 0.22 of the reads in RAW water, increasing this percentage to 16.68% ± 2.48 in UV water. The separation between RAW and UV samples was further tested with ANOSIM (phylum, class, order, and family R = 1) and PERMANOVA (phylum, class, order, and family F = 30.39, 34.77, 52.55, and 17.29, respectively). Altogether, these results indicate that wastewater treatment is particularly efficient in the removal of bacterial classes comprising human commensals and bacterial pathogens.

**Figure 5:**
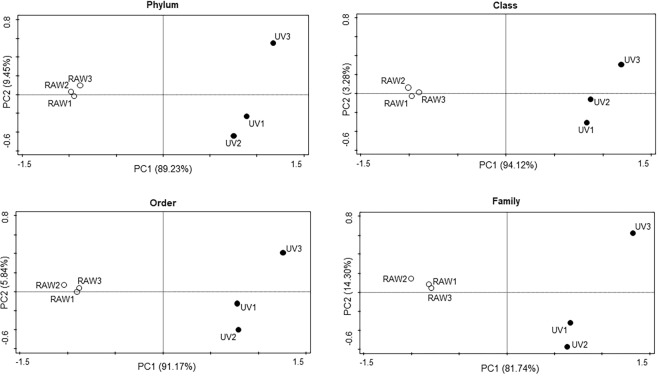
Principal Component Analysis (PCA) of bacterial composition in the different samples before and after wastewater treatment for different taxonomical levels: phylum, class, order, and family. Only the dimensions accounting for most of the variance are shown. White dots represent the influent (RAW1, RAW2, RAW3), and the black dots represent the final effluent samples (UV1, UV2, UV3).

A Principal Component Analysis (PCA) based on the wastewater microbiome classified from Phylum to Family, showed that the RAW microbial communities clustered together, irrespective of sampling date, in contrast to the treated water samples that displayed a larger dispersion (Fig. 5).

## Discussion

Understanding how bacterial pathogens acquire AR and how this resistance spreads and is maintained requires a One Health approach in which all ecosystems potentially involved in the process are analysed^1,2,10^. Among them, water bodies have been proposed to be relevant habitats for the origin and spread of AR^17,25^. Given that water reuse is increasingly needed, understanding the impact of WWT on AR is fundamental in order to optimize those treatments aiming at reducing the prevalence of ARGs. For instance, recent analysis of the microbiome of household water filters suggests that, despite of being a good alternative for in-house water treatment, they can also deteriorate the quality of tap water by acting as reservoirs for sludge/slime deposit that might eventually promote biofilm formation and microbial growth in the home water distribution system^52^.

The recent rise of antibiotic resistant bacteria is one of the few evolution processes that can be studied in real time and, as any evolution process, presents deterministic and stochastic aspects^53,54^. Measures for fighting resistance require predicting the outcome of the intervention, in other words, require that evolution follows a deterministic pattern^55,56^. Our PCA results indicate that influent samples clustered together when analysed in terms of bacterial composition and distribution of ARGs and plasmid replicon types, while, comparatively, samples from treated water were less homogeneous and in this regard, evolution presents some degree of stochasticity.

In agreement with previously published work^35^ these data suggest that the microbiota and the resistome of the water received by an urban WWTP may represent the overall composition of the microbiome of all the population from a city - the urban microbiome and its corresponding resistome. While seasonal variations in the urban microbiome and the resistome can be expected^57^, tracking non-seasonal alterations of this urban microbiome/resistome might serve for detecting overall changes in the city population, which, as stated by other authors can be a tool for stewardship of epidemic outbreaks eventually involved in the dissemination of ARGs^35^.

Despite the observed inter-sample differences in UV-treated water, some aspects were common to all samples, and some general conclusions can be raised concerning the effect of WWT, at least in this WWTP.

We found that WWT reduces the percentage of *Proteobacteria* and *Firmicutes* present in water, particularly those classes such as *Gammaproteobacteria*, or *Epsilonproteobacteria*, which include several human pathogens and commensals. In this regard, it is important to highlight that *Proteobacteria* and *Firmicutes* include several of the most important human bacterial pathogens as *Escherichia coli*, *Salmonella*, *Klebsiella pneumoniae*, *Enterococcus*, *Staphylococcus aureus*, *Acinetobacter baumannii* or *Pseudomonas aeruginosa*. Several of these organisms belong to the group of ESKAPE pathogens, which have been listed as those bacterial species with a higher risk of acquiring multiple antibiotic resistance^58,59^. Since these organisms are hubs for the acquisition and spread of ARGs^60–64^, we may expect that WWT produces a parallel decline in the amount of acquired ARGs and MGEs. Indeed, our results show that the number of reads of ARGs per ten millions of reads, which measures the relative abundance (respecting the total number of reads) of mobile ARGs and of plasmids among all genes present in the sample, is also much lower in treated wastewater than in RAW water. Beta-lactams and aminoglycosides were the antibiotics families recruiting a larger amount of different resistance genes. However the higher number of reads belonged to macrolide resistance genes, mainly *msrE* and *mphE*. Plasmids as those belonging to the repUS2 group, which have been recently found to be prevalent in gut microbiome^40^, as well as the IncP(6) and IncQ groups that include several multidrug resistance plasmids^41–45^ were highly abundant in RAW water and their presence was much lower after UV treatment. To note here that IncQ2 plasmids have been recently found in urban agriculture fields irrigated with wastewater in Africa cities^65^ and that IncP-6 plasmids have been involved in carbapenem-resistance circulation among several *Enterobacteriaceae* species from wastewater and a hospital^43^. The efficient removal of these elements may then contribute to palliate its dissemination. In parallel, a strong reduction in the amount of intrinsic ARGs, including those belonging to *Enterobacteriaceae* was also observed. The ARGs decline is even higher if the amount of DNA per ml, lower in the case of treated water (Table S8), is taken into consideration. Altogether, our results show that bacteria of human origin, which are important potential ARGs and MGEs carriers, are removed during WWT, at least in the studied WWTP. The reduction of these bacteria can be explained either because they are more sensitive that the other taxons enriched after treatment to the stressful conditions of a WWTP, or as the consequence of the competition promoted by strictly environmental micoorganisms that are abundant and more fitted in the wastewater ecosystem. These results highlight the importance of the indigenous microbiota on the removal of bacteria with an anthropogenic origin.

## Materials and Methods

### Sampling

Sampling was performed, as described^37^ in a WWTP located in Northern Portugal that combines activated sludge secondary treatment and UV disinfection. The plant processes an average of 35900 m^3^ daily, corresponding to 170000 inhabitant equivalents. The plant includes a bar screen and a homogenization chamber to remove large solids. Small solids and fats are removed using grit and grease removal chambers and a primary settling tank was used for removing settable solids. Organic residues, N and P were removed by an activated sludge biological treatment that includes recirculation between aerobic and anoxic tanks. UV disinfection was performed using an open channel UV system (Trojan, UV3000HO), with 38 × 8 150 W lamps per channel and a contact time of 11.44 s, which corresponds to a dose of 29.7 mJ/cm^2^.

The characteristics of the raw and final effluent are provided in Supplementary material, Table S8. As described before^37^, three grab wastewater samples were collected in sterile flasks from raw wastewater after the first settling tank, while treated water was sampled at the end of the WWT process. The samples were transported in refrigerated containers and processed within 12 h after sampling.

### DNA extraction, and sequencing

Each DNA extract was prepared from a pool of 12 DNA extracts obtained each from 25 ml (RAW water) or 300 mL (treated water). So, in total DNA was obtained from either 300 ml of RAW water or from 3.6 l of treated water. In each case, the samples were filtered through 0.22 µm polycarbonate membranes (Whatmann) and stored at -80 °C until DNA was extracted using the PowerWater DNA isolation kit (MOBIO Laboratories Inc), following manufacturer’s instructions. The amount of DNA per ml in each sample is shown in Table S9. For total metagenomic analysis, total DNA was used for constructing six independent libraries with 500–600 bp of insert size length. The libraries were sequenced using a MiSeq platform (2 × 250) (Illumina) at the Parque Científico de Madrid Facility (Madrid, Spain). The numbers or reads ranged from 6066088 to 8172260 (Table S9). DNA sequences have been deposited at NCBI, Bioproject PRJNA532515. Accession numbers for raw samples: SAMN11401900, SAMN11413482, SAMN11413483; for treated samples: SAMN11413484, SAMN11413485, SAMN11413486.

### Analysis of shotgun metagenomic data

We used SqueezeMeta v1.0^66^ for the annotation of ARG and taxa of the raw metagenomic reads. This pipeline allows a full analysis of metagenomic data, from trimming to annotation. Reads with less than 100 bp and low quality score under a Phred value of 25 were discarded. The taxonomic classification was obtained by direct homology searches against GenBank NR database (release 223, December 2017) using Diamond (v0.9.13.114) with a maximum e-value threshold of 1e-03. The annotations were done using a last-common ancestor (LCA) algorithm. LCA first selects the hits having at least 80% of the bitscore of the best hit and overcoming the minimum identity threshold set for a particular taxonomic rank (55, 50, 46, 42 and 40% for family, order, class, phylum and superkingdom ranks, respectively). This means that in order to classify a sequence at the phylum taxonomic rank, for instance, hits for that sequence must be at last 42% identical. Then it looks for the common taxon for all hits at the desired taxonomic rank (although some flexibility is allowed. for instance admitting one outlier if the number of hits is high). In case that a common taxon is not found, the read is unassigned. For the annotation of antibiotic resistance genes, CARD release 3.0.4 (21/08/19), was used as the reference functional classification, and the reads were annotated using the best hit to this CARD database fulfilling 95% identity and 95% alignment coverage of the query. The results from this annotation were manually curated. Plasmid replicon types present in the samples were identified by performing a local alignment of all reads from each sample against the sequences deposited at the PlasmidFinder v1.3 database^67^, once downloaded and formatted as database in a local server. For comparative purposes, the relative abundance of replicons and of ARGs was normalized by the number of reads present in 10 million of total reads.

### Statistical analysis

Principal components analysis (PCA) was performed to explore the relationship between RAW and UV samples bacterial community structure, ARGs known to be present in mobile elements in bacterial pathogens and plasmid replicons using Canoco 5.01 software^68^. The separation of RWW and UV samples was tested using the ANOSIM and PERMANOVA tests, with the Bray-Curtis model, using PAST v3.1^69^. An ANOSIM R value close to 1.0 suggests dissimilarity between RAW and UV samples while an R value close to 0 suggests an even distribution of high and low ranks within and between RAW and UV samples. The higher PERMANOVA F-ratios indicate a more pronounced separation between RAW and UV samples. Statistically significant differences were calculated with *t-*test for paired samples assuming equal variances.

## Supplementary information

Supplementary information.
