# Supplementary material for: Metagenomic analysis of an urban resistome before and after wastewater treatment
Source: Sci Rep. 2020 May 18;10:8174. doi: 10.1038/s41598-020-65031-y (PMC7235214; doi:10.1038/s41598-020-65031-y)
Supplement: Supplementary file 1 — Supplementary information. [file 41598_2020_65031_MOESM1_ESM.docx]

**Supplementary information of the article**

**Metagenomic analysis of an urban resistome before and after wastewater treatment**

Felipe Lira. Ivone Vaz-Moreira, Javier Tamames, Célia M. Manaia, José Luis Martínez

**Table S1: Mobile resistance genes**

| **CARD ID** | **Number of reads por ten millions of total reads*** | | | | | | | **Name** | **Phenotype** |
| --- | --- | --- | --- | --- | --- | --- | --- | --- | --- |
|  | **Total** | **RAW1** | **RAW2** | **RAW3** | **UV1** | **UV2** | **UV3** |  |  |
| ARO:3003109 | 490.64 | 967.29 | 658.68 | 503.39 | 96.76 | 25.80 | 23.83 | *msrE* | Macrolides |
| ARO:3003741 | 210.50 | 393.44 | 312.17 | 213.20 | 16.13 | 8.60 | 11.92 | *mphE* | Macrolides |
| ARO:3000191 | 112.70 | 218.79 | 123.31 | 157.93 | 24.19 | 4.30 | 0.00 | *tetQ* | Tetracyclines |
| ARO:3000410 | 84.12 | 122.83 | 103.02 | 122.39 | 12.10 | 21.50 | 26.81 | *sul1* | Sulfonamides |
| ARO:3002660 | 52.73 | 80.61 | 62.43 | 90.81 | 4.03 | 8.60 | 0.00 | *APH(6)-Id* | Aminoglicosides |
| ARO:3000194 | 37.03 | 42.22 | 45.26 | 78.96 | 4.03 | 0.00 | 0.00 | *tetW* | Tetracyclines |
| ARO:3002613 | 35.02 | 63.33 | 31.22 | 63.17 | 0.00 | 0.00 | 5.96 | *aadA13* | Aminoglicosides |
| ARO:3000167 | 41.05 | 46.06 | 62.43 | 55.27 | 0.00 | 21.50 | 14.90 | *tet(C)* | Tetracyclines |
| ARO:3000316 | 29.78 | 34.55 | 43.70 | 51.33 | 8.06 | 0.00 | 0.00 | *mphA* | Macrolides |
| ARO:3000205 | 30.59 | 46.06 | 37.46 | 47.38 | 0.00 | 8.60 | 5.96 | *tetX* | Tetracyclines |
| ARO:3002620 | 35.42 | 57.58 | 43.70 | 47.38 | 0.00 | 17.20 | 5.96 | *aadA23* | Aminoglicosides |
| ARO:3003839 | 36.22 | 69.09 | 43.70 | 47.38 | 4.03 | 4.30 | 0.00 | *mrx* | Macrolides |
| ARO:3002639 | 57.56 | 92.12 | 101.45 | 45.40 | 16.13 | 8.60 | 2.98 | *APH(3'')-Ib* | Aminoglicosides |
| ARO:3002695 | 15.70 | 23.03 | 10.93 | 39.48 | 0.00 | 0.00 | 0.00 | *cmlA5* | Choramphenicol |
| ARO:3001502 | 15.70 | 21.11 | 15.61 | 33.56 | 0.00 | 4.30 | 0.00 | *OXA-256* | Beta-lactams |
| ARO:3000498 | 27.37 | 40.30 | 49.95 | 29.61 | 0.00 | 0.00 | 0.00 | *ErmF* | Macrolides |
| ARO:3001397 | 10.46 | 11.52 | 10.93 | 25.66 | 0.00 | 0.00 | 0.00 | *OXA-2* | Beta-lactams |
| ARO:3001800 | 15.29 | 24.95 | 14.05 | 25.66 | 12.10 | 0.00 | 0.00 | *OXA-226* | Beta-lactams |
| ARO:3000566 | 46.69 | 92.12 | 85.85 | 25.66 | 0.00 | 0.00 | 0.00 | *tet(39)* | Tetracyclines |
| ARO:3000375 | 10.87 | 15.35 | 12.49 | 21.71 | 0.00 | 0.00 | 0.00 | *ErmB* | Macrolides |
| ARO:3000361 | 18.92 | 15.35 | 35.90 | 21.71 | 8.06 | 0.00 | 8.94 | *EreA* | Macrolides |
| ARO:3000197 | 8.45 | 1.92 | 14.05 | 17.77 | 0.00 | 0.00 | 5.96 | *tet36* | Tetracyclines |
| ARO:3000616 | 14.89 | 24.95 | 23.41 | 17.77 | 0.00 | 0.00 | 0.00 | *mel* | Macrolides |
| ARO:3002611 | 16.10 | 13.43 | 32.78 | 17.77 | 4.03 | 4.30 | 2.98 | *aadA11* | Aminoglicosides |
| ARO:3004504 | 4.02 | 0.00 | 1.56 | 15.79 | 4.03 | 0.00 | 0.00 | *MCR-3.10* | Polymixin |
| ARO:3004503 | 6.44 | 11.52 | 3.12 | 15.79 | 0.00 | 0.00 | 0.00 | *MCR-3.6* | Polymixin |
| ARO:3002385 | 7.65 | 5.76 | 3.12 | 15.79 | 8.06 | 8.60 | 5.96 | *BEL-1* | Beta-lactams |
| ARO:3002622 | 10.46 | 7.68 | 18.73 | 15.79 | 4.03 | 4.30 | 0.00 | *aadA6/aadA10* | Aminoglicosides |
| ARO:3000190 | 13.68 | 24.95 | 18.73 | 15.79 | 4.03 | 0.00 | 0.00 | *tetO* | Tetracyclines |
| ARO:3002607 | 6.44 | 11.52 | 4.68 | 13.82 | 0.00 | 0.00 | 0.00 | *aadA7* | Aminoglicosides |
| ARO:3002547 | 7.24 | 5.76 | 12.49 | 13.82 | 0.00 | 0.00 | 0.00 | *AAC(6')-Ib-cr* | Aminoglicosides |
| ARO:3003923 | 8.45 | 19.19 | 6.24 | 13.82 | 0.00 | 0.00 | 0.00 | *oqxB* | Multidrug |
| ARO:3002676 | 8.85 | 24.95 | 3.12 | 13.82 | 0.00 | 0.00 | 0.00 | *catB3* | Choramphenicol |
| ARO:3002191 | 9.26 | 17.27 | 10.93 | 13.82 | 0.00 | 0.00 | 0.00 | *OXA-372* | Beta-lactams |
| ARO:3002617 | 10.46 | 11.52 | 20.29 | 13.82 | 0.00 | 0.00 | 0.00 | *aadA17* | Aminoglicosides |
| ARO:3003003 | 5.23 | 3.84 | 4.68 | 11.84 | 8.06 | 0.00 | 0.00 | *CfxA3* | Beta-lactams |
| ARO:3001781 | 6.84 | 9.60 | 9.37 | 11.84 | 0.00 | 0.00 | 0.00 | *OXA-47* | Beta-lactams |
| ARO:3003742 | 11.67 | 19.19 | 20.29 | 11.84 | 0.00 | 0.00 | 0.00 | *mphG* | Macrolides |
| ARO:3002605 | 14.09 | 17.27 | 31.22 | 11.84 | 0.00 | 0.00 | 0.00 | *aadA5* | Aminoglicosides |
| ARO:3003206 | 3.22 | 5.76 | 0.00 | 9.87 | 0.00 | 0.00 | 0.00 | *lsaE* | Lincosamides |
| ARO:3004139 | 3.62 | 1.92 | 4.68 | 9.87 | 0.00 | 0.00 | 0.00 | *MCR-3.1* | Polymixin |
| ARO:3004442 | 4.83 | 7.68 | 3.12 | 9.87 | 4.03 | 0.00 | 0.00 | *tet(W/N/W)* | Tetracyclines |
| ARO:3002826 | 7.24 | 13.43 | 7.80 | 9.87 | 0.00 | 0.00 | 2.98 | *EreA2* | Macrolides |
| ARO:3000196 | 8.85 | 23.03 | 7.80 | 9.87 | 0.00 | 0.00 | 0.00 | *tet32* | Tetracyclines |
| ARO:3003745 | 10.46 | 19.19 | 17.17 | 9.87 | 0.00 | 0.00 | 0.00 | *mefC* | Macrolides |
| ARO:3001793 | 11.67 | 9.60 | 29.66 | 9.87 | 0.00 | 0.00 | 0.00 | *OXA-320* | Beta-lactams |
| ARO:3002332 | 2.41 | 0.00 | 3.12 | 7.90 | 0.00 | 0.00 | 0.00 | *GES-3* | Beta-lactams |
| ARO:3003836 | 2.41 | 0.00 | 3.12 | 7.90 | 0.00 | 0.00 | 0.00 | *qacH* | Quinolones |
| ARO:3000556 | 3.22 | 5.76 | 1.56 | 7.90 | 0.00 | 0.00 | 0.00 | *tet(44)* | Tetracyclines |
| ARO:3001405 | 4.02 | 3.84 | 6.24 | 7.90 | 0.00 | 0.00 | 0.00 | *OXA-10* | Beta-lactams |
| ARO:3002372 | 5.23 | 3.84 | 10.93 | 7.90 | 0.00 | 0.00 | 0.00 | *VEB-3* | Beta-lactams |
| ARO:3001429 | 6.84 | 19.19 | 4.68 | 7.90 | 0.00 | 0.00 | 0.00 | *OXA-35* | Beta-lactams |
| ARO:3002601 | 7.65 | 7.68 | 17.17 | 7.90 | 0.00 | 0.00 | 0.00 | *aadA* | Aminoglicosides |
| ARO:3002594 | 1.61 | 1.92 | 0.00 | 5.92 | 0.00 | 0.00 | 0.00 | *AAC(6')-IIa* | Aminoglicosides |
| ARO:3000928 | 1.61 | 1.92 | 0.00 | 5.92 | 0.00 | 0.00 | 0.00 | *TEM-59* | Beta-lactams |
| ARO:3002858 | 2.01 | 3.84 | 0.00 | 5.92 | 0.00 | 0.00 | 0.00 | *dfrA12* | Trimethoprim |
| ARO:3002157 | 2.41 | 0.00 | 4.68 | 5.92 | 0.00 | 0.00 | 0.00 | *FOX-3* | Beta-lactams |
| ARO:3002186 | 2.41 | 1.92 | 3.12 | 5.92 | 0.00 | 0.00 | 0.00 | *MOX-3* | Beta-lactams |
| ARO:3002350 | 3.22 | 3.84 | 4.68 | 5.92 | 0.00 | 0.00 | 0.00 | *GES-21* | Beta-lactams |
| ARO:3001500 | 3.22 | 3.84 | 3.12 | 5.92 | 0.00 | 0.00 | 2.98 | *OXA-251* | Beta-lactams |
| ARO:3002837 | 3.62 | 0.00 | 9.37 | 5.92 | 0.00 | 0.00 | 0.00 | *lnuC* | Lincosamides |
| ARO:3004239 | 3.62 | 3.84 | 6.24 | 5.92 | 0.00 | 0.00 | 0.00 | *NPS-1* | Beta-lactams |
| ARO:3000522 | 3.62 | 5.76 | 4.68 | 5.92 | 0.00 | 0.00 | 0.00 | *ErmG* | Macrolides |
| ARO:3002546 | 4.43 | 9.60 | 3.12 | 5.92 | 0.00 | 4.30 | 0.00 | *AAC(6')-Ib* | Aminoglicosides |
| ARO:3002641 | 6.04 | 3.84 | 14.05 | 5.92 | 4.03 | 0.00 | 0.00 | *APH(3')-Ia* | Aminoglicosides |
| ARO:3000173 | 6.44 | 0.00 | 20.29 | 5.92 | 0.00 | 0.00 | 0.00 | *tet(E)* | Tetracyclines |
| ARO:3003107 | 6.44 | 7.68 | 10.93 | 5.92 | 8.06 | 0.00 | 0.00 | *mef(B)* | Macrolides |
| ARO:3000412 | 17.31 | 32.63 | 26.53 | 5.92 | 0.00 | 4.30 | 14.90 | *sul2* | Sulfonamides |
| ARO:3002604 | 0.80 | 0.00 | 0.00 | 3.95 | 0.00 | 0.00 | 0.00 | *aadA4* | Aminoglicosides |
| ARO:3002862 | 1.21 | 0.00 | 1.56 | 3.95 | 0.00 | 0.00 | 0.00 | *dfrA7* | Trimethoprim |
| ARO:3002628 | 1.61 | 1.92 | 1.56 | 3.95 | 0.00 | 0.00 | 0.00 | *aad(6)* | Aminoglicosides |
| ARO:3001396 | 1.61 | 1.92 | 1.56 | 3.95 | 0.00 | 0.00 | 0.00 | *OXA-1* | Beta-lactams |
| ARO:3001416 | 2.01 | 0.00 | 4.68 | 3.95 | 0.00 | 0.00 | 0.00 | *OXA-21* | Beta-lactams |
| ARO:3000904 | 2.01 | 1.92 | 3.12 | 3.95 | 0.00 | 0.00 | 0.00 | *TEM-34* | Beta-lactams |
| ARO:3001410 | 2.01 | 5.76 | 0.00 | 3.95 | 0.00 | 0.00 | 0.00 | *OXA-15* | Beta-lactams |
| ARO:3001439 | 2.41 | 3.84 | 3.12 | 3.95 | 0.00 | 0.00 | 0.00 | *OXA-101* | Beta-lactams |
| ARO:3001407 | 2.41 | 7.68 | 0.00 | 3.95 | 0.00 | 0.00 | 0.00 | *OXA-12* | Beta-lactams |
| ARO:3001797 | 2.82 | 1.92 | 6.24 | 3.95 | 0.00 | 0.00 | 0.00 | *OXA-46* | Beta-lactams |
| ARO:3002647 | 2.82 | 9.60 | 0.00 | 3.95 | 0.00 | 0.00 | 0.00 | *APH(3')-IIIa* | Aminoglicosides |
| ARO:3001399 | 3.22 | 1.92 | 7.80 | 3.95 | 0.00 | 0.00 | 0.00 | *OXA-4* | Beta-lactams |
| ARO:3000186 | 3.62 | 5.76 | 6.24 | 3.95 | 0.00 | 0.00 | 0.00 | *tetM* | Tetracyclines |
| ARO:3002343 | 4.43 | 3.84 | 10.93 | 3.95 | 0.00 | 0.00 | 0.00 | *GES-14* | Beta-lactams |
| ARO:3002794 | 8.85 | 21.11 | 14.05 | 3.95 | 0.00 | 0.00 | 0.00 | *QnrS5* | Quinolones |
| ARO:3004091 | 0.40 | 0.00 | 0.00 | 1.97 | 0.00 | 0.00 | 0.00 | *ANT(3'')-IIc* | Aminoglicosides |
| ARO:3002626 | 0.40 | 0.00 | 0.00 | 1.97 | 0.00 | 0.00 | 0.00 | *ANT(6)-Ia* | Aminoglicosides |
| ARO:3002630 | 0.40 | 0.00 | 0.00 | 1.97 | 0.00 | 0.00 | 0.00 | *ANT(9)-Ia* | Aminoglicosides |
| ARO:3001822 | 0.40 | 0.00 | 0.00 | 1.97 | 0.00 | 0.00 | 0.00 | *ACT-2* | Beta-lactams |
| ARO:3002036 | 0.40 | 0.00 | 0.00 | 1.97 | 0.00 | 0.00 | 0.00 | *CMY-25* | Beta-lactams |
| ARO:3002184 | 0.40 | 0.00 | 0.00 | 1.97 | 0.00 | 0.00 | 0.00 | *MOX-4* | Beta-lactams |
| ARO:3001415 | 0.40 | 0.00 | 0.00 | 1.97 | 0.00 | 0.00 | 0.00 | *OXA-20* | Beta-lactams |
| ARO:3001430 | 0.40 | 0.00 | 0.00 | 1.97 | 0.00 | 0.00 | 0.00 | *OXA-36* | Beta-lactams |
| ARO:3001431 | 0.40 | 0.00 | 0.00 | 1.97 | 0.00 | 0.00 | 0.00 | *OXA-37* | Beta-lactams |
| ARO:3001045 | 0.40 | 0.00 | 0.00 | 1.97 | 0.00 | 0.00 | 0.00 | *TEM-183* | Beta-lactams |
| ARO:3002378 | 0.40 | 0.00 | 0.00 | 1.97 | 0.00 | 0.00 | 0.00 | *VEB-9* | Beta-lactams |
| ARO:3002691 | 0.40 | 0.00 | 0.00 | 1.97 | 0.00 | 0.00 | 0.00 | *cmlA* | Choramphenicol |
| ARO:3002693 | 0.40 | 0.00 | 0.00 | 1.97 | 0.00 | 0.00 | 0.00 | *cmlA1* | Choramphenicol |
| ARO:3002705 | 0.40 | 0.00 | 0.00 | 1.97 | 0.00 | 0.00 | 0.00 | *floR* | Choramphenicol |
| ARO:3004332 | 0.80 | 0.00 | 1.56 | 1.97 | 0.00 | 0.00 | 0.00 | *MCR-5.1* | Pplymixin |
| ARO:3002155 | 0.80 | 1.92 | 0.00 | 1.97 | 0.00 | 0.00 | 0.00 | *FOX-1* | Beta-lactams |
| ARO:3001412 | 0.80 | 1.92 | 0.00 | 1.97 | 0.00 | 0.00 | 0.00 | *OXA-17* | Beta-lactams |
| ARO:3000567 | 0.80 | 1.92 | 0.00 | 1.97 | 0.00 | 0.00 | 0.00 | *tet(40)* | Tetracyclines |
| ARO:3003013 | 1.21 | 0.00 | 3.12 | 1.97 | 0.00 | 0.00 | 0.00 | *dfrA15* | Trimethoprim |
| ARO:3002346 | 1.21 | 1.92 | 1.56 | 1.97 | 0.00 | 0.00 | 0.00 | *GES-17* | Beta-lactams |
| ARO:3002606 | 1.61 | 5.76 | 0.00 | 1.97 | 0.00 | 0.00 | 0.00 | *aadA6* | Aminoglicosides |
| ARO:3001775 | 2.01 | 3.84 | 3.12 | 1.97 | 0.00 | 0.00 | 0.00 | *OXA-119* | Beta-lactams |
| ARO:3003096 | 2.82 | 5.76 | 4.68 | 1.97 | 0.00 | 0.00 | 0.00 | *CfxA5* | Beta-lactams |
| ARO:3002859 | 4.02 | 0.00 | 14.05 | 1.97 | 0.00 | 0.00 | 0.00 | *dfrA14* | Trimethoprim |
| ARO:3002986 | 4.83 | 5.76 | 10.93 | 1.97 | 4.03 | 0.00 | 0.00 | *bacA* | Bacitracin |
| ARO:3002183 | 4.83 | 9.60 | 9.37 | 1.97 | 0.00 | 0.00 | 0.00 | *MOX-2* | Beta-lactams |
| ARO:3003197 | 5.23 | 3.84 | 12.49 | 1.97 | 0.00 | 4.30 | 2.98 | *aadA25* | Aminoglicosides |
| ARO:3003905 | 5.23 | 9.60 | 10.93 | 1.97 | 0.00 | 0.00 | 0.00 | *ANT(4')-Ib* | Aminoglicosides |
| ARO:3002602 | 6.04 | 13.43 | 10.93 | 1.97 | 0.00 | 0.00 | 0.00 | *aadA2* | Aminoglicosides |
| ARO:3002599 | 6.04 | 13.43 | 7.80 | 1.97 | 0.00 | 8.60 | 0.00 | *AAC(6')-30/AAC(6')-Ib'* | Aminoglicosides |
| ARO:3002531 | 0.40 | 0.00 | 1.56 | 0.00 | 0.00 | 0.00 | 0.00 | *AAC(3)-Ic* | Aminoglicosides |
| ARO:3002612 | 0.40 | 0.00 | 1.56 | 0.00 | 0.00 | 0.00 | 0.00 | *aadA12* | Aminoglicosides |
| ARO:3002240 | 0.40 | 0.00 | 1.56 | 0.00 | 0.00 | 0.00 | 0.00 | *CARB-1* | Beta-lactams |
| ARO:3002242 | 0.40 | 0.00 | 1.56 | 0.00 | 0.00 | 0.00 | 0.00 | *CARB-3* | Beta-lactams |
| ARO:3002053 | 0.40 | 0.00 | 1.56 | 0.00 | 0.00 | 0.00 | 0.00 | *CMY-42* | Beta-lactams |
| ARO:3002162 | 0.40 | 0.00 | 1.56 | 0.00 | 0.00 | 0.00 | 0.00 | *FOX-10* | Beta-lactams |
| ARO:3002158 | 0.40 | 0.00 | 1.56 | 0.00 | 0.00 | 0.00 | 0.00 | *FOX-4* | Beta-lactams |
| ARO:3002341 | 0.40 | 0.00 | 1.56 | 0.00 | 0.00 | 0.00 | 0.00 | *GES-12* | Beta-lactams |
| ARO:3002344 | 0.40 | 0.00 | 1.56 | 0.00 | 0.00 | 0.00 | 0.00 | *GES-15* | Beta-lactams |
| ARO:3002353 | 0.40 | 0.00 | 1.56 | 0.00 | 0.00 | 0.00 | 0.00 | *GES-24* | Beta-lactams |
| ARO:3001810 | 0.40 | 0.00 | 1.56 | 0.00 | 0.00 | 0.00 | 0.00 | *OXA-53* | Beta-lactams |
| ARO:3001404 | 0.40 | 0.00 | 1.56 | 0.00 | 0.00 | 0.00 | 0.00 | *OXA-9* | Beta-lactams |
| ARO:3001015 | 0.40 | 0.00 | 1.56 | 0.00 | 0.00 | 0.00 | 0.00 | *TEM-148* | Beta-lactams |
| ARO:3001392 | 0.40 | 0.00 | 1.56 | 0.00 | 0.00 | 0.00 | 0.00 | *TEM-215* | Beta-lactams |
| ARO:3002680 | 0.40 | 0.00 | 1.56 | 0.00 | 0.00 | 0.00 | 0.00 | *catB8* | Chloramphanicol |
| ARO:3004510 | 0.40 | 0.00 | 1.56 | 0.00 | 0.00 | 0.00 | 0.00 | *MCR-3.7* | Polymixin |
| ARO:3002528 | 0.40 | 1.92 | 0.00 | 0.00 | 0.00 | 0.00 | 0.00 | *AAC(3)-Ia* | Aminoglicosides |
| ARO:3002066 | 0.40 | 1.92 | 0.00 | 0.00 | 0.00 | 0.00 | 0.00 | *CMY-55* | Beta-lactams |
| ARO:3001426 | 0.40 | 1.92 | 0.00 | 0.00 | 0.00 | 0.00 | 0.00 | *OXA-32* | Beta-lactams |
| ARO:3001133 | 0.40 | 1.92 | 0.00 | 0.00 | 0.00 | 0.00 | 0.00 | *SHV-79* | Beta-lactams |
| ARO:3002694 | 0.40 | 1.92 | 0.00 | 0.00 | 0.00 | 0.00 | 0.00 | *cmlA4* | Chloramphanicol |
| ARO:3004454 | 0.40 | 1.92 | 0.00 | 0.00 | 0.00 | 0.00 | 0.00 | *CmR* | Choramphenicol |
| ARO:3002703 | 0.40 | 1.92 | 0.00 | 0.00 | 0.00 | 0.00 | 0.00 | *cmx* | Choramphenicol |
| ARO:3002798 | 0.40 | 1.92 | 0.00 | 0.00 | 0.00 | 0.00 | 0.00 | *QnrS9* | Quinolones |
| ARO:3002801 | 0.40 | 1.92 | 0.00 | 0.00 | 0.00 | 0.00 | 0.00 | *QnrVC4* | Quinolones |
| ARO:3002600 | 0.40 | 0.00 | 0.00 | 0.00 | 0.00 | 4.30 | 0.00 | *AAC(3)-Ib/AAC(6')-Ib''* | Aminoglicosides |
| ARO:3002629 | 0.80 | 0.00 | 3.12 | 0.00 | 0.00 | 0.00 | 0.00 | *ANT(6)-Ib* | Aminoglicosides |
| ARO:3001788 | 0.80 | 0.00 | 3.12 | 0.00 | 0.00 | 0.00 | 0.00 | *OXA-204* | Beta-lactams |
| ARO:3002683 | 0.80 | 0.00 | 3.12 | 0.00 | 0.00 | 0.00 | 0.00 | *catI* | Choramphenicol |
| ARO:3002879 | 0.80 | 0.00 | 3.12 | 0.00 | 0.00 | 0.00 | 0.00 | *linG* | Lincosamide |
| ARO:3003071 | 0.80 | 0.00 | 3.12 | 0.00 | 0.00 | 0.00 | 0.00 | *mphF* | Macrolides |
| ARO:3004361 | 0.80 | 0.00 | 3.12 | 0.00 | 0.00 | 0.00 | 0.00 | *sul4* | Sulfonamides |
| ARO:3002687 | 0.80 | 0.00 | 3.12 | 0.00 | 0.00 | 0.00 | 0.00 | *catQ* | Tetracyclines |
| ARO:3000174 | 0.80 | 0.00 | 1.56 | 0.00 | 4.03 | 0.00 | 0.00 | *tet(G)* | Tetracyclines |
| ARO:3000230 | 0.80 | 3.84 | 0.00 | 0.00 | 0.00 | 0.00 | 0.00 | *ANT(2'')-Ia* | Aminoglicosides |
| ARO:3002156 | 0.80 | 3.84 | 0.00 | 0.00 | 0.00 | 0.00 | 0.00 | *FOX-2* | Beta-lactams |
| ARO:3002376 | 0.80 | 3.84 | 0.00 | 0.00 | 0.00 | 0.00 | 0.00 | *VEB-7* | Beta-lactams |
| ARO:3002853 | 0.80 | 3.84 | 0.00 | 0.00 | 0.00 | 0.00 | 0.00 | *arr-8* | Rifamycin |
| ARO:3002616 | 0.80 | 1.92 | 0.00 | 0.00 | 0.00 | 4.30 | 0.00 | *aadA16* | Aminoglicosides |
| ARO:3001400 | 0.80 | 0.00 | 0.00 | 0.00 | 0.00 | 0.00 | 5.96 | *OXA-5* | Beta-lactams |
| ARO:3001662 | 1.21 | 3.84 | 1.56 | 0.00 | 0.00 | 0.00 | 0.00 | *OXA-164* | Beta-lactams |
| ARO:3002997 | 1.21 | 5.76 | 0.00 | 0.00 | 0.00 | 0.00 | 0.00 | *LCR-1* | Beta-lactams |
| ARO:3001406 | 1.61 | 0.00 | 6.24 | 0.00 | 0.00 | 0.00 | 0.00 | *OXA-11* | Beta-lactams |
| ARO:3001811 | 1.61 | 1.92 | 3.12 | 0.00 | 0.00 | 0.00 | 2.98 | *OXA-129* | Beta-lactams |
| ARO:3002596 | 2.01 | 0.00 | 7.80 | 0.00 | 0.00 | 0.00 | 0.00 | *AAC(6')-IIc* | Aminoglicosides |
| ARO:3000988 | 2.01 | 3.84 | 4.68 | 0.00 | 0.00 | 0.00 | 0.00 | *TEM-126* | Beta-lactams |
| ARO:3002848 | 2.01 | 5.76 | 3.12 | 0.00 | 0.00 | 0.00 | 0.00 | *arr-3* | Rifamycin |
| ARO:3001411 | 2.41 | 5.76 | 4.68 | 0.00 | 0.00 | 0.00 | 0.00 | *OXA-16* | Beta-lactams |
| ARO:3001483 | 4.83 | 11.52 | 6.24 | 0.00 | 4.03 | 4.30 | 0.00 | *OXA-205* | Beta-lactams |
| ARO:3001425 | 5.23 | 1.92 | 18.73 | 0.00 | 0.00 | 0.00 | 0.00 | *OXA-31* | Beta-lactams |
| **Total reads** | **2180.30** | **3596.62** | **3017.11** | **2783.44** | **383.01** | **210.72** | **166.84** |  |  |

* The number of reads corresponding to each ARG was normalized taken into consideration the number of reads of each sample and expressed as the number of ARG reads per ten millions of total reads.

**Table S2 Plasmid replicons**

| **Plasmid replicon types** |  | **Number of reads por ten millions of total reads*** | | | | | |
| --- | --- | --- | --- | --- | --- | --- | --- |
|  |  | **RAW1** | **RAW2** | **RAW3** | **RAW4** | **RAW5** | **RAW6** |
| repUS2 | 11551 | 1983 | 1311 | 8164 | 76 | 17 | 0 |
| IncP(6) | 7316 | 731 | 887 | 5652 | 31 | 0 | 15 |
| IncQ2 | 7004 | 764 | 699 | 5495 | 31 | 0 | 15 |
| ColRNAI | 511 | 114 | 38 | 314 | 31 | 0 | 15 |
| IncQ1 | 3369 | 683 | 300 | 2355 | 15 | 17 | 0 |
| IncP(Beta) | 586 | 0 | 38 | 471 | 15 | 17 | 46 |
| p0111 | 172 | 0 | 0 | 157 | 15 | 0 | 0 |
| IncR | 81 | 16 | 50 | 0 | 15 | 0 | 0 |
| Col(IRGK) | 75 | 33 | 25 | 0 | 0 | 17 | 0 |
| pXuzhou21 | 17 | 0 | 0 | 0 | 0 | 17 | 0 |
| ColKP3 | 2802 | 341 | 262 | 2198 | 0 | 0 | 0 |
| IncU | 1213 | 146 | 125 | 942 | 0 | 0 | 0 |
| IncFIB(K) | 1045 | 65 | 38 | 942 | 0 | 0 | 0 |
| Col(Ye4449) | 695 | 49 | 175 | 471 | 0 | 0 | 0 |
| repUS18 | 566 | 33 | 63 | 471 | 0 | 0 | 0 |
| IncA/C2 | 500 | 16 | 12 | 471 | 0 | 0 | 0 |
| Col(MGD2) | 355 | 16 | 25 | 314 | 0 | 0 | 0 |
| IncFII | 339 | 0 | 25 | 314 | 0 | 0 | 0 |
| IncFIB(pECLA) | 330 | 16 | 0 | 314 | 0 | 0 | 0 |
| Col(IMGS31) | 339 | 0 | 25 | 314 | 0 | 0 | 0 |
| repUS6 | 260 | 65 | 38 | 157 | 0 | 0 | 0 |
| pENTAS02 | 182 | 0 | 25 | 157 | 0 | 0 | 0 |
| IncHI1B(CIT) | 218 | 49 | 12 | 157 | 0 | 0 | 0 |
| IncFIB(AP001918) | 186 | 16 | 12 | 157 | 0 | 0 | 0 |
| Col(pWES) | 169 | 0 | 12 | 157 | 0 | 0 | 0 |
| IncFII(pECLA) | 169 | 0 | 12 | 157 | 0 | 0 | 0 |
| IncFII(pRSB107) | 169 | 0 | 12 | 157 | 0 | 0 | 0 |
| IncN | 222 | 65 | 0 | 157 | 0 | 0 | 0 |
| IncHI1A(CIT) | 190 | 33 | 0 | 157 | 0 | 0 | 0 |
| TrfA | 173 | 16 | 0 | 157 | 0 | 0 | 0 |
| Col(BS512) | 157 | 0 | 0 | 157 | 0 | 0 | 0 |
| IncB/O/K/Z | 157 | 0 | 0 | 157 | 0 | 0 | 0 |
| IncFIB(pB171) | 157 | 0 | 0 | 157 | 0 | 0 | 0 |
| IncFIB(pQil) | 157 | 0 | 0 | 157 | 0 | 0 | 0 |
| IncFII(pCRY) | 157 | 0 | 0 | 157 | 0 | 0 | 0 |
| IncFII(pENTA) | 157 | 0 | 0 | 157 | 0 | 0 | 0 |
| IncFII(pSE11) | 157 | 0 | 0 | 157 | 0 | 0 | 0 |
| IncX4 | 157 | 0 | 0 | 157 | 0 | 0 | 0 |
| repA | 52 | 16 | 35 | 0 | 0 | 0 | 0 |
| ColpVC | 58 | 33 | 25 | 0 | 0 | 0 | 0 |
| IncFIC(FII) | 25 | 0 | 25 | 0 | 0 | 0 | 0 |
| IncFIA(HI1) | 45 | 33 | 12 | 0 | 0 | 0 | 0 |
| Col156 | 29 | 16 | 12 | 0 | 0 | 0 | 0 |
| ColE10 | 29 | 16 | 12 | 0 | 0 | 0 | 0 |
| Col8282 | 12 | 0 | 12 | 0 | 0 | 0 | 0 |
| IncFIB(pHCM2) | 12 | 0 | 12 | 0 | 0 | 0 | 0 |
| IncHI2 | 12 | 0 | 12 | 0 | 0 | 0 | 0 |
| IncHI2A | 12 | 0 | 12 | 0 | 0 | 0 | 0 |
| IncL/M(pMU407) | 12 | 0 | 12 | 0 | 0 | 0 | 0 |
| IncY | 12 | 0 | 12 | 0 | 0 | 0 | 0 |
| rep11 | 12 | 0 | 12 | 0 | 0 | 0 | 0 |
| rep18 | 12 | 0 | 12 | 0 | 0 | 0 | 0 |
| Col(MG828) | 81 | 81 | 0 | 0 | 0 | 0 | 0 |
| IncFII(S) | 33 | 33 | 0 | 0 | 0 | 0 | 0 |
| IncFII(Yp) | 33 | 33 | 0 | 0 | 0 | 0 | 0 |
| Col3M | 16 | 16 | 0 | 0 | 0 | 0 | 0 |
| IncFII(SARC14) | 16 | 16 | 0 | 0 | 0 | 0 | 0 |
| IncI1 | 16 | 16 | 0 | 0 | 0 | 0 | 0 |
| IncN2 | 16 | 16 | 0 | 0 | 0 | 0 | 0 |
| rep21 | 16 | 16 | 0 | 0 | 0 | 0 | 0 |
| **Total** | **42621** | **5591** | **4442** | **32184** | **228** | **85** | **91** |

* The number of reads corresponding to each replicon was normalized taken into consideration the number of reads of each sample and expressed as the number of replicon reads per ten millions of total reads.

**Table S3: Intrinsic resistance genes**

| **CARD ID** | **Total** | **Number of reads por ten millions of total reads*** | | | | | | | **Organism** | **Phenotype** |
| --- | --- | --- | --- | --- | --- | --- | --- | --- | --- | --- |
|  |  | **RAW1** | **RAW2** | **RAW3** | **UV1** | **UV2** | **UV3** | **Name** |  |  |
| ARO:3001631 | 0.80 | 1.92 | 1.56 | 0.00 | 0.00 | 0.00 | 0.00 | *OXA-96* | *Acinetobacter baumannii* | Beta-lactams |
| ARO:3001420 | 1.21 | 5.76 | 0.00 | 0.00 | 0.00 | 0.00 | 0.00 | *OXA-25* | *Acinetobacter baumannii* | Beta-lactams |
| ARO:3001521 | 7.65 | 11.52 | 10.93 | 11.84 | 0.00 | 0.00 | 0.00 | *OXA-333* | *Acinetobacter johnsonii* | Beta-lactams |
| ARO:3001710 | 9.66 | 19.19 | 15.61 | 7.90 | 0.00 | 0.00 | 0.00 | *OXA-211* | *Acinetobacter johnsonii* | Beta-lactams |
| ARO:3001522 | 2.01 | 3.84 | 1.56 | 3.95 | 0.00 | 0.00 | 0.00 | *OXA-334* | *Acinetobacter johnsonii* | Beta-lactams |
| ARO:3001711 | 3.22 | 11.52 | 0.00 | 3.95 | 0.00 | 0.00 | 0.00 | *OXA-212* | *Acinetobacter johnsonii* | Beta-lactams |
| ARO:3001504 | 2.01 | 7.68 | 0.00 | 1.97 | 0.00 | 0.00 | 0.00 | *OXA-309* | *Acinetobacter johnsonii* | Beta-lactams |
| ARO:3002185 | 13.68 | 19.19 | 7.80 | 37.51 | 0.00 | 0.00 | 0.00 | *MOX-6* | *Aeromonas caviae* | Beta-lactams |
| ARO:3002188 | 5.63 | 5.76 | 4.68 | 15.79 | 0.00 | 0.00 | 0.00 | *MOX-5* | *Aeromonas caviae* | Beta-lactams |
| ARO:3002189 | 1.61 | 1.92 | 0.00 | 5.92 | 0.00 | 0.00 | 0.00 | *MOX-7* | *Aeromonas caviae* | Beta-lactams |
| ARO:3002190 | 1.61 | 1.92 | 1.56 | 3.95 | 0.00 | 0.00 | 0.00 | *MOX-8* | *Aeromonas caviae* | Beta-lactams |
| ARO:3002481 | 2.82 | 0.00 | 9.37 | 1.97 | 0.00 | 0.00 | 0.00 | *AER-1* | *Aeromonas hydrophila* | Beta-lactams |
| ARO:3003103 | 0.40 | 0.00 | 0.00 | 1.97 | 0.00 | 0.00 | 0.00 | *cphA7* | *Aeromonas hydrophilia* | Beta-lactams |
| ARO:3003094 | 0.40 | 1.92 | 0.00 | 0.00 | 0.00 | 0.00 | 0.00 | *imiH* | *Aeromonas hydrophilia* | Beta-lactams |
| ARO:3003104 | 0.40 | 0.00 | 1.56 | 0.00 | 0.00 | 0.00 | 0.00 | *cphA8* | *Aeromonas sobria* | Beta-lactams |
| ARO:3003100 | 2.41 | 0.00 | 4.68 | 5.92 | 0.00 | 0.00 | 0.00 | *cphA4* | *Aeromonas veronii* | Beta-lactams |
| ARO:3003093 | 1.21 | 5.76 | 0.00 | 0.00 | 0.00 | 0.00 | 0.00 | *cphA3* | *Aeromonas veronii* | Beta-lactams |
| ARO:3003005 | 2.41 | 1.92 | 3.12 | 5.92 | 0.00 | 0.00 | 0.00 | *CfxA4* | *Bacteroides* | Beta-lactams |
| ARO:3001777 | 0.80 | 0.00 | 3.12 | 0.00 | 0.00 | 0.00 | 0.00 | *OXA-347* | *Bacteroides* | Beta-lactams |
| ARO:3002999 | 4.02 | 5.76 | 1.56 | 3.95 | 8.06 | 8.60 | 0.00 | *CblA-1* | *Bacteroides uniformis* | Beta-lactams |
| ARO:3002083 | 0.40 | 0.00 | 0.00 | 1.97 | 0.00 | 0.00 | 0.00 | *CMY-70* | *Citrobacter (Enterobacteriaceae)* | Beta-lactams |
| ARO:3002106 | 0.40 | 0.00 | 0.00 | 0.00 | 0.00 | 4.30 | 0.00 | *CMY-93* | *Citrobacter freundii (Enterobacteriaceae)* | Beta-lactams |
| ARO:3002092 | 0.80 | 0.00 | 3.12 | 0.00 | 0.00 | 0.00 | 0.00 | *CMY-79* | *Citrobacter freundii (Enterobacteriaceae)* | Beta-lactams |
| ARO:3000842 | 0.80 | 3.84 | 0.00 | 0.00 | 0.00 | 0.00 | 0.00 | *EBR-1* | *Empedobacter brevis* | Beta-lactams |
| ARO:3001855 | 0.80 | 0.00 | 3.12 | 0.00 | 0.00 | 0.00 | 0.00 | *ACT-35* | *Enterobacter cloacae (Enterobacteriaceae)* | Beta-lactams |
| ARO:3003173 | 0.40 | 1.92 | 0.00 | 0.00 | 0.00 | 0.00 | 0.00 | *MIR-17* | *Enterobacter cloacae (Enterobacteriaceae)* | Beta-lactams |
| ARO:3000237 | 5.63 | 3.84 | 4.68 | 13.82 | 0.00 | 0.00 | 5.96 | *TolC* | *Enterobacteriaceae* | Multidrug |
| ARO:3000491 | 16.10 | 24.95 | 24.97 | 13.82 | 12.10 | 0.00 | 2.98 | *acrD* | *Enterobacteriaceae* | Multidrug |
| ARO:3000816 | 1.61 | 0.00 | 1.56 | 0.00 | 8.06 | 0.00 | 2.98 | *mtrA* | *Enterobacteriaceae* | Multidrug |
| ARO:3000254 | 2.41 | 3.84 | 1.56 | 0.00 | 8.06 | 4.30 | 0.00 | *emrY* | *Enterobacteriaceae* | Multidrug |
| ARO:3003550 | 4.02 | 7.68 | 4.68 | 3.95 | 0.00 | 4.30 | 0.00 | *mdtP* | *Enterobacteriaceae* | Multidrug |
| ARO:3000216 | 20.12 | 19.19 | 24.97 | 39.48 | 16.13 | 0.00 | 0.00 | *acrB* | *Enterobacteriaceae* | Multidrug |
| ARO:3000793 | 9.26 | 13.43 | 7.80 | 15.79 | 12.10 | 0.00 | 0.00 | *mdtB* | *Enterobacteriaceae* | Multidrug |
| ARO:3000074 | 9.66 | 15.35 | 6.24 | 19.74 | 8.06 | 0.00 | 0.00 | *emrB* | *Enterobacteriaceae* | Multidrug |
| ARO:3000796 | 8.85 | 15.35 | 12.49 | 7.90 | 8.06 | 0.00 | 0.00 | *mdtF* | *Enterobacteriaceae* | Multidrug |
| ARO:3003549 | 5.23 | 11.52 | 4.68 | 3.95 | 8.06 | 0.00 | 0.00 | *mdtO* | *Enterobacteriaceae* | Multidrug |
| ARO:3004043 | 3.62 | 0.00 | 7.80 | 5.92 | 4.03 | 0.00 | 0.00 | *acrA* | *Enterobacteriaceae* | Multidrug |
| ARO:3004290 | 1.61 | 1.92 | 3.12 | 0.00 | 4.03 | 0.00 | 0.00 | *ampC* | *Enterobacteriaceae* | Beta-lactams |
| ARO:3000502 | 7.65 | 15.35 | 6.24 | 13.82 | 0.00 | 0.00 | 0.00 | *AcrF* | *Enterobacteriaceae* | Multidrug |
| ARO:3003952 | 7.24 | 17.27 | 3.12 | 13.82 | 0.00 | 0.00 | 0.00 | *YojI* | *Enterobacteriaceae* | Microcin J25 |
| ARO:3000794 | 6.44 | 15.35 | 3.12 | 11.84 | 0.00 | 0.00 | 0.00 | *mdtC* | *Enterobacteriaceae* | Multidrug |
| ARO:3001329 | 4.02 | 5.76 | 3.12 | 9.87 | 0.00 | 0.00 | 0.00 | *mdtG* | *Enterobacteriaceae* | Multidrug |
| ARO:3000795 | 5.63 | 9.60 | 7.80 | 7.90 | 0.00 | 0.00 | 0.00 | *mdtE* | *Enterobacteriaceae* | Multidrug |
| ARO:3000499 | 4.02 | 3.84 | 7.80 | 5.92 | 0.00 | 0.00 | 0.00 | *AcrE* | *Enterobacteriaceae* | Multidrug |
| ARO:3004042 | 2.82 | 0.00 | 6.24 | 5.92 | 0.00 | 0.00 | 0.00 | *acrA* | *Enterobacteriaceae* | Multidrug |
| ARO:3003950 | 5.23 | 13.43 | 4.68 | 5.92 | 0.00 | 0.00 | 0.00 | *msbA* | *Enterobacteriaceae* | Multidrug |
| ARO:3000206 | 2.82 | 1.92 | 6.24 | 3.95 | 0.00 | 0.00 | 0.00 | *emrK* | *Enterobacteriaceae* | Multidrug |
| ARO:3000027 | 2.41 | 3.84 | 3.12 | 3.95 | 0.00 | 0.00 | 0.00 | *emrA* | *Enterobacteriaceae* | Multidrug |
| ARO:3000781 | 2.82 | 7.68 | 1.56 | 3.95 | 0.00 | 0.00 | 0.00 | *adeJ* | *Enterobacteriaceae* | Multidrug |
| ARO:3001328 | 1.21 | 1.92 | 0.00 | 3.95 | 0.00 | 0.00 | 0.00 | *mdfA* | *Enterobacteriaceae* | Multidrug |
| ARO:3003548 | 3.22 | 1.92 | 9.37 | 1.97 | 0.00 | 0.00 | 0.00 | *mdtN* | *Enterobacteriaceae* | Multidrug |
| ARO:3001216 | 2.01 | 1.92 | 6.24 | 0.00 | 0.00 | 0.00 | 0.00 | *mdtH* | *Enterobacteriaceae* | Multidrug |
| ARO:3000792 | 1.61 | 0.00 | 6.24 | 0.00 | 0.00 | 0.00 | 0.00 | *mdtA* | *Enterobacteriaceae* | Multidrug |
| ARO:3001214 | 1.61 | 3.84 | 3.12 | 0.00 | 0.00 | 0.00 | 0.00 | *mdtM* | *Enterobacteriaceae* | Multidrug |
| ARO:3004041 | 0.80 | 1.92 | 1.56 | 0.00 | 0.00 | 0.00 | 0.00 | *acrA* | *Enterobacteriaceae* | Multidrug |
| ARO:3002127 | 0.80 | 3.84 | 0.00 | 0.00 | 0.00 | 0.00 | 0.00 | *CMY-116* | *Enterobacteriaceae* | Beta-lactams |
| ARO:3003949 | 0.80 | 3.84 | 0.00 | 0.00 | 0.00 | 0.00 | 0.00 | *efrB* | *Enterococcus* | Multidrug |
| ARO:3002882 | 1.21 | 1.92 | 3.12 | 0.00 | 0.00 | 0.00 | 0.00 | *lmrD* | *Lactococcus lactis* | Beta-lactams |
| ARO:3004292 | 2.01 | 0.00 | 6.24 | 1.97 | 0.00 | 0.00 | 0.00 | *ampC* | *Laribacter hongkongensis* | Beta-lactams |
| ARO:3003002 | 58.76 | 71.01 | 98.33 | 80.94 | 20.16 | 0.00 | 0.00 | *CfxA2* | *Prevotella intermedia* | Beta-lactams |
| ARO:3003097 | 13.68 | 36.47 | 12.49 | 11.84 | 0.00 | 4.30 | 0.00 | *CfxA6* | *Prevotella spp.* | Beta-lactams |
| ARO:3000804 | 2.01 | 3.84 | 3.12 | 1.97 | 0.00 | 0.00 | 0.00 | *MexF* | *Pseudomonas* | Multidrug |
| ARO:3000378 | 1.21 | 1.92 | 1.56 | 1.97 | 0.00 | 0.00 | 0.00 | *MexB* | *Pseudomonas* | Multidrug |
| ARO:3003693 | 1.21 | 1.92 | 1.56 | 1.97 | 0.00 | 0.00 | 0.00 | *MexK* | *Pseudomonas* | Multidrug |
| ARO:3004074 | 0.80 | 0.00 | 1.56 | 1.97 | 0.00 | 0.00 | 0.00 | *MuxB* | *Pseudomonas* | Multidrug |
| ARO:3000805 | 0.80 | 0.00 | 1.56 | 1.97 | 0.00 | 0.00 | 0.00 | *OprN* | *Pseudomonas* | Multidrug |
| ARO:3000801 | 0.40 | 0.00 | 0.00 | 1.97 | 0.00 | 0.00 | 0.00 | *MexD* | *Pseudomonas* | Multidrug |
| ARO:3003031 | 0.40 | 0.00 | 0.00 | 1.97 | 0.00 | 0.00 | 0.00 | *MexW* | *Pseudomonas* | Multidrug |
| ARO:3000809 | 0.40 | 0.00 | 1.56 | 0.00 | 0.00 | 0.00 | 0.00 | *OpmD* | *Pseudomonas* | Multidrug |
| ARO:3004075 | 0.40 | 1.92 | 0.00 | 0.00 | 0.00 | 0.00 | 0.00 | *MuxC* | *Pseudomonas* | Multidrug |
| ARO:3003563 | 0.40 | 1.92 | 0.00 | 0.00 | 0.00 | 0.00 | 0.00 | *RCP-1* | *Rhodobacter capsulatus* | Beta-lactams |
| ARO:3003056 | 1.21 | 3.84 | 0.00 | 1.97 | 0.00 | 0.00 | 0.00 | *smeE* | *Stenotrophomonas maltophilia* | Multidrug |
| ARO:3003053 | 0.40 | 0.00 | 1.56 | 0.00 | 0.00 | 0.00 | 0.00 | *smeC* | *Stenotrophomonas maltophilia* | Multidrug |
| **Total** | **304,28** | **466,37** | **390,21** | **442,19** | **116,92** | **25,80** | **11,92** |  |  |  |

* The number of reads corresponding to each ARG was normalized taken into consideration the number of reads of each sample and expressed as the number of ARG reads per ten millions of total reads.

**Table S4 Number of reads for each bacterial phylum**

| **Phylum** | **Total** | **RAW1** | **RAW2** | **RAW3** | **UV1** | **UV2** | **UV3** |
| --- | --- | --- | --- | --- | --- | --- | --- |
| k_Bacteria;p_Proteobacteria; | 14029617 | 3430805 | 4238872 | 3315236 | 889573 | 804373 | 1350758 |
| k_Bacteria;n_FCB group;n_Bacteroidetes/Chlorobi group;p_Bacteroidetes; | 3743329 | 1049685 | 1242519 | 877546 | 190563 | 225196 | 157820 |
| k_Bacteria;n_Terrabacteria group;p_Firmicutes; | 1482356 | 407024 | 468959 | 473556 | 49629 | 49612 | 33576 |
| k_Bacteria;n_Terrabacteria group;p_Actinobacteria; | 834400 | 64103 | 55244 | 70103 | 196899 | 136109 | 311942 |
| k_Bacteria;n_Terrabacteria group;p_Chloroflexi; | 400955 | 9944 | 4099 | 21685 | 43074 | 33927 | 288226 |
| k_Bacteria;n_unclassified Bacteria;n_Bacteria candidate phyla;p_Candidatus Saccharibacteria; | 179884 | 8857 | 11514 | 10192 | 57841 | 52335 | 39145 |
| k_Bacteria;n_PVC group;p_Verrucomicrobia; | 137238 | 11409 | 26208 | 21344 | 23271 | 17999 | 37007 |
| k_Bacteria;n_PVC group;p_Planctomycetes; | 90246 | 3307 | 2535 | 3230 | 27326 | 16307 | 37541 |
| k_Bacteria;p_Acidobacteria; | 81057 | 4101 | 7097 | 5116 | 15336 | 9054 | 40353 |
| k_Bacteria;n_unclassified Bacteria;n_Bacteria candidate phyla;n_Patescibacteria group;n_Parcubacteria group;p_Candidatus Moranbacteria; | 73619 | 1109 | 1771 | 1008 | 17727 | 28858 | 23146 |
| k_Bacteria;p_Nitrospirae; | 64027 | 1553 | 752 | 929 | 20162 | 13332 | 27299 |
| k_Bacteria;p_Fusobacteria; | 59045 | 13292 | 27045 | 17285 | 570 | 568 | 285 |
| k_Bacteria;n_unclassified Bacteria;n_Bacteria candidate phyla;n_Patescibacteria group;n_Parcubacteria group;p_Candidatus Nomurabacteria; | 49808 | 424 | 956 | 578 | 11798 | 18368 | 17684 |
| k_Bacteria;n_unclassified Bacteria;n_Bacteria candidate phyla;p_Candidatus Peregrinibacteria; | 46718 | 272 | 1077 | 816 | 8067 | 19650 | 16836 |
| k_Bacteria;n_Terrabacteria group;n_Cyanobacteria/Melainabacteria group;p_Candidatus Melainabacteria; | 43838 | 4119 | 5104 | 4222 | 10283 | 9795 | 10315 |
| k_Bacteria;n_unclassified Bacteria;n_Bacteria candidate phyla;n_Patescibacteria group;n_Microgenomates group;p_Candidatus Roizmanbacteria; | 39485 | 557 | 797 | 379 | 13446 | 16284 | 8022 |
| k_Bacteria;n_unclassified Bacteria;n_Bacteria candidate phyla;p_Candidatus Sumerlaeota; | 38416 | 990 | 1132 | 804 | 17759 | 10056 | 7675 |
| k_Bacteria;p_Spirochaetes; | 33724 | 2704 | 4535 | 3708 | 7742 | 4523 | 10512 |
| k_Bacteria;p_Elusimicrobia; | 30583 | 842 | 4511 | 1935 | 8735 | 6643 | 7917 |
| k_Bacteria;n_Terrabacteria group;n_Cyanobacteria/Melainabacteria group;p_Cyanobacteria; | 29916 | 1399 | 1830 | 1788 | 7831 | 6135 | 10933 |
| k_Bacteria;n_unclassified Bacteria;n_Bacteria candidate phyla;n_Patescibacteria group;n_Parcubacteria group;p_Candidatus Parcubacteria; | 25298 | 378 | 664 | 511 | 9383 | 8195 | 6167 |
| k_Bacteria;n_unclassified Bacteria;n_Bacteria candidate phyla;n_Patescibacteria group;n_Parcubacteria group;p_Candidatus Magasanikbacteria; | 24778 | 241 | 604 | 148 | 6599 | 12535 | 4651 |
| k_Bacteria;n_PVC group;p_Candidatus Omnitrophica; | 24179 | 488 | 1032 | 1483 | 7601 | 7050 | 6525 |
| k_Bacteria;n_unclassified Bacteria;n_Bacteria candidate phyla;n_Patescibacteria group;n_Microgenomates group;p_Candidatus Levybacteria; | 23431 | 355 | 585 | 287 | 8149 | 9738 | 4317 |
| k_Bacteria;n_PVC group;p_Chlamydiae; | 22370 | 842 | 960 | 863 | 8370 | 5025 | 6310 |
| k_Bacteria;n_unclassified Bacteria;n_Bacteria candidate phyla;n_Patescibacteria group;n_Parcubacteria group;p_Candidatus Uhrbacteria; | 21794 | 638 | 1356 | 609 | 5664 | 6967 | 6560 |
| k_Bacteria;n_PVC group;p_Lentisphaerae; | 20637 | 2656 | 5990 | 6722 | 2448 | 1524 | 1297 |
| k_Bacteria;p_Synergistetes; | 19330 | 2976 | 10950 | 3762 | 640 | 643 | 359 |
| k_Bacteria;n_unclassified Bacteria;n_Bacteria candidate phyla;n_Patescibacteria group;n_Parcubacteria group;p_Candidatus Kaiserbacteria; | 19125 | 132 | 331 | 322 | 4254 | 6107 | 7979 |
| k_Bacteria;n_unclassified Bacteria;n_Bacteria candidate phyla;n_Patescibacteria group;n_Parcubacteria group;p_Candidatus Taylorbacteria; | 18512 | 71 | 87 | 53 | 9820 | 5584 | 2897 |
| k_Bacteria;n_unclassified Bacteria;n_Bacteria candidate phyla;n_Patescibacteria group;n_Microgenomates group;p_Candidatus Woesebacteria; | 17537 | 268 | 475 | 341 | 6083 | 6003 | 4367 |
| k_Bacteria;n_FCB group;p_Gemmatimonadetes; | 12661 | 405 | 121 | 243 | 2173 | 1249 | 8470 |
| k_Bacteria;n_unclassified Bacteria;n_Bacteria candidate phyla;n_Patescibacteria group;n_Parcubacteria group;p_Candidatus Staskawiczbacteria; | 10216 | 27 | 144 | 27 | 1243 | 8143 | 632 |
| k_Bacteria;n_unclassified Bacteria;n_Bacteria candidate phyla;n_Patescibacteria group;n_Microgenomates group;p_Candidatus Microgenomates; | 9760 | 120 | 262 | 82 | 2602 | 4796 | 1898 |
| k_Bacteria;n_unclassified Bacteria;n_Bacteria candidate phyla;p_Candidatus Doudnabacteria; | 8688 | 80 | 185 | 100 | 2537 | 3567 | 2219 |
| k_Bacteria;n_unclassified Bacteria;n_Bacteria candidate phyla;n_Patescibacteria group;p_Candidatus Gracilibacteria; | 8666 | 1572 | 1598 | 930 | 1321 | 1426 | 1819 |
| k_Bacteria;n_unclassified Bacteria;n_Bacteria candidate phyla;n_Patescibacteria group;n_Microgenomates group;p_Candidatus Gottesmanbacteria; | 7703 | 112 | 191 | 108 | 2373 | 2888 | 2031 |
| k_Bacteria;n_unclassified Bacteria;n_Bacteria candidate phyla;n_Patescibacteria group;n_Parcubacteria group;p_Candidatus Zambryskibacteria; | 6123 | 39 | 60 | 81 | 1458 | 2244 | 2241 |
| k_Bacteria;n_Terrabacteria group;p_Armatimonadetes; | 6035 | 424 | 297 | 266 | 2144 | 959 | 1945 |
| k_Bacteria;n_unclassified Bacteria;n_Bacteria candidate phyla;n_Patescibacteria group;n_Parcubacteria group;p_Candidatus Falkowbacteria; | 5683 | 116 | 206 | 147 | 1453 | 1753 | 2008 |
| k_Bacteria;n_FCB group;n_Bacteroidetes/Chlorobi group;p_Ignavibacteriae; | 5570 | 283 | 326 | 270 | 1325 | 822 | 2544 |
| k_Bacteria;n_unclassified Bacteria;n_Bacteria candidate phyla;n_Patescibacteria group;n_Microgenomates group;p_Candidatus Shapirobacteria; | 5148 | 145 | 138 | 133 | 1798 | 1699 | 1235 |
| k_Bacteria;n_unclassified Bacteria;n_Bacteria candidate phyla;n_Patescibacteria group;n_Microgenomates group;p_Candidatus Daviesbacteria; | 4876 | 54 | 112 | 48 | 1496 | 2097 | 1069 |
| k_Bacteria;n_Terrabacteria group;p_Deinococcus-Thermus; | 4782 | 294 | 283 | 189 | 1262 | 1422 | 1332 |
| k_Bacteria;n_FCB group;n_Bacteroidetes/Chlorobi group;p_Chlorobi; | 4691 | 352 | 238 | 273 | 1528 | 873 | 1427 |
| k_Bacteria;n_unclassified Bacteria;n_Bacteria candidate phyla;n_Patescibacteria group;n_Microgenomates group;p_Candidatus Pacebacteria; | 4427 | 59 | 160 | 94 | 1158 | 2129 | 827 |
| k_Bacteria;n_FCB group;p_Fibrobacteres; | 4266 | 1079 | 1412 | 1068 | 219 | 361 | 127 |
| k_Bacteria;n_unclassified Bacteria;n_Bacteria candidate phyla;n_Patescibacteria group;n_Microgenomates group;p_Candidatus Collierbacteria; | 4138 | 45 | 99 | 32 | 1324 | 1823 | 815 |
| k_Bacteria;p_Deferribacteres; | 4021 | 388 | 2260 | 1246 | 43 | 54 | 30 |
| k_Bacteria;n_unclassified Bacteria;n_Bacteria candidate phyla;n_Patescibacteria group;n_Microgenomates group;p_Candidatus Amesbacteria; | 3762 | 53 | 99 | 31 | 1097 | 1694 | 788 |
| k_Bacteria;n_Terrabacteria group;p_Tenericutes; | 3424 | 663 | 765 | 612 | 448 | 667 | 269 |
| k_Bacteria;n_FCB group;p_Candidatus Cloacimonetes; | 3256 | 584 | 1060 | 594 | 366 | 358 | 294 |
| k_Bacteria;n_unclassified Bacteria;n_Bacteria candidate phyla;p_Candidatus Rokubacteria; | 2992 | 167 | 156 | 145 | 663 | 472 | 1389 |
| k_Bacteria;n_unclassified Bacteria;n_Bacteria candidate phyla;n_Patescibacteria group;n_Parcubacteria group;p_Candidatus Adlerbacteria; | 2595 | 30 | 37 | 31 | 772 | 708 | 1017 |
| k_Bacteria;n_unclassified Bacteria;n_Bacteria candidate phyla;n_Patescibacteria group;n_Parcubacteria group;p_Candidatus Lloydbacteria; | 2352 | 30 | 38 | 61 | 757 | 587 | 879 |
| k_Bacteria;n_unclassified Bacteria;n_Bacteria candidate phyla;p_Candidatus Berkelbacteria; | 2185 | 48 | 65 | 25 | 822 | 753 | 472 |
| k_Bacteria;n_unclassified Bacteria;n_Bacteria candidate phyla;p_Candidatus Riflebacteria; | 2157 | 336 | 780 | 385 | 177 | 158 | 321 |
| k_Bacteria;n_unclassified Bacteria;n_Bacteria candidate phyla;n_Patescibacteria group;n_Parcubacteria group;p_Candidatus Vogelbacteria; | 2021 | 19 | 30 | 21 | 694 | 790 | 467 |
| k_Bacteria;n_unclassified Bacteria;n_Bacteria candidate phyla;p_Candidatus Schekmanbacteria; | 2014 | 18 | 64 | 33 | 649 | 782 | 468 |
| k_Bacteria;n_unclassified Bacteria;n_Bacteria candidate phyla;n_Patescibacteria group;n_Parcubacteria group;p_Candidatus Yanofskybacteria; | 1991 | 39 | 58 | 34 | 696 | 701 | 463 |
| k_Bacteria;n_unclassified Bacteria;n_Bacteria candidate phyla;n_Patescibacteria group;n_Parcubacteria group;p_Candidatus Wolfebacteria; | 1978 | 30 | 67 | 45 | 602 | 764 | 470 |
| k_Bacteria;n_unclassified Bacteria;n_Bacteria candidate phyla;n_Patescibacteria group;n_Parcubacteria group;p_Candidatus Kerfeldbacteria; | 1941 | 12 | 40 | 35 | 563 | 600 | 691 |
| k_Bacteria;p_Thermotogae; | 1894 | 211 | 278 | 214 | 379 | 613 | 199 |
| k_Bacteria;n_unclassified Bacteria;n_Bacteria candidate phyla;p_Candidatus Dadabacteria; | 1865 | 59 | 39 | 38 | 651 | 502 | 576 |
| k_Bacteria;n_unclassified Bacteria;n_Bacteria candidate phyla;n_Patescibacteria group;n_Parcubacteria group;p_Candidatus Giovannonibacteria; | 1683 | 27 | 34 | 21 | 595 | 389 | 617 |
| k_Bacteria;n_unclassified Bacteria;n_Bacteria candidate phyla;p_Candidatus Eisenbacteria; | 1636 | 112 | 18 | 15 | 540 | 232 | 719 |
| k_Bacteria;p_Aquificae; | 1447 | 274 | 371 | 332 | 158 | 166 | 146 |
| k_Bacteria;n_unclassified Bacteria;n_Bacteria candidate phyla;n_Patescibacteria group;n_Parcubacteria group;p_Candidatus Yonathbacteria; | 1409 | 22 | 45 | 22 | 335 | 535 | 450 |
| k_Bacteria;n_unclassified Bacteria;n_Bacteria candidate phyla;n_Patescibacteria group;n_Parcubacteria group;p_Candidatus Harrisonbacteria; | 1277 | 18 | 8 | 4 | 176 | 355 | 716 |
| k_Bacteria;n_unclassified Bacteria;n_Bacteria candidate phyla;n_Patescibacteria group;n_Parcubacteria group;p_Candidatus Campbellbacteria; | 1221 | 12 | 27 | 17 | 309 | 486 | 370 |
| k_Bacteria;n_unclassified Bacteria;n_Bacteria candidate phyla;n_Patescibacteria group;n_Parcubacteria group;p_Candidatus Buchananbacteria; | 1213 | 17 | 28 | 10 | 410 | 414 | 334 |
| k_Bacteria;n_FCB group;p_Candidatus Marinimicrobia; | 1094 | 175 | 234 | 166 | 202 | 172 | 145 |
| k_Bacteria;n_unclassified Bacteria;n_Bacteria candidate phyla;n_Patescibacteria group;n_Parcubacteria group;p_Candidatus Nealsonbacteria; | 1011 | 14 | 39 | 23 | 328 | 376 | 231 |
| k_Bacteria;n_FCB group;p_Candidatus Fermentibacteria; | 855 | 18 | 58 | 43 | 232 | 344 | 160 |
| k_Bacteria;n_unclassified Bacteria;n_Bacteria candidate phyla;n_Patescibacteria group;n_Parcubacteria group;p_Candidatus Sungbacteria; | 837 | 13 | 27 | 6 | 361 | 258 | 172 |
| k_Bacteria;n_unclassified Bacteria;n_Bacteria candidate phyla;n_Patescibacteria group;n_Microgenomates group;p_Candidatus Curtissbacteria; | 816 | 23 | 30 | 18 | 296 | 305 | 144 |
| k_Bacteria;n_unclassified Bacteria;n_Bacteria candidate phyla;p_Candidatus Atribacteria; | 805 | 19 | 40 | 24 | 269 | 270 | 183 |
| k_Bacteria;n_FCB group;p_Candidatus Hydrogenedentes; | 736 | 63 | 40 | 86 | 115 | 60 | 372 |
| k_Bacteria;n_Nitrospinae/Tectomicrobia group;p_Nitrospinae; | 715 | 18 | 28 | 12 | 394 | 177 | 86 |
| k_Bacteria;n_unclassified Bacteria;n_Bacteria candidate phyla;n_Patescibacteria group;n_Microgenomates group;p_Candidatus Woykebacteria; | 700 | 8 | 11 | 10 | 158 | 248 | 265 |
| k_Bacteria;n_unclassified Bacteria;n_Bacteria candidate phyla;n_Patescibacteria group;n_Parcubacteria group;p_Candidatus Jorgensenbacteria; | 662 | 6 | 12 | 5 | 252 | 239 | 148 |
| k_Bacteria;n_Terrabacteria group;n_Cyanobacteria/Melainabacteria group;p_Candidatus Margulisbacteria; | 657 | 21 | 38 | 15 | 215 | 186 | 182 |
| k_Bacteria;n_unclassified Bacteria;n_Bacteria candidate phyla;p_Candidatus Aminicenantes; | 640 | 142 | 76 | 104 | 104 | 69 | 145 |
| k_Bacteria;n_unclassified Bacteria;n_Bacteria candidate phyla;p_Candidatus Abawacabacteria; | 636 | 1 | 9 | 14 | 225 | 94 | 293 |
| k_Bacteria;n_unclassified Bacteria;n_Bacteria candidate phyla;n_Patescibacteria group;n_Parcubacteria group;p_Candidatus Liptonbacteria; | 549 | 10 | 14 | 14 | 159 | 152 | 200 |
| k_Bacteria;n_unclassified Bacteria;n_Bacteria candidate phyla;n_Patescibacteria group;n_Parcubacteria group;p_Candidatus Azambacteria; | 537 | 9 | 9 | 2 | 165 | 186 | 166 |
| k_Bacteria;n_FCB group;p_Candidatus Latescibacteria; | 522 | 19 | 68 | 73 | 123 | 84 | 155 |
| k_Bacteria;n_unclassified Bacteria;n_Bacteria candidate phyla;n_Patescibacteria group;n_Microgenomates group;p_Candidatus Beckwithbacteria; | 513 | 5 | 10 | 2 | 166 | 209 | 121 |
| k_Bacteria;n_unclassified Bacteria;n_Bacteria candidate phyla;p_Candidatus Handelsmanbacteria; | 504 | 12 | 12 | 8 | 195 | 121 | 156 |
| k_Bacteria;n_unclassified Bacteria;n_Bacteria candidate phyla;n_Patescibacteria group;n_Parcubacteria group;p_Candidatus Ryanbacteria; | 480 | 4 | 8 | 9 | 168 | 176 | 115 |
| k_Bacteria;n_unclassified Bacteria;n_Bacteria candidate phyla;n_Patescibacteria group;n_Parcubacteria group;p_Candidatus Komeilibacteria; | 474 | 9 | 53 | 28 | 154 | 113 | 117 |
| k_Bacteria;n_unclassified Bacteria;n_Bacteria candidate phyla;n_Patescibacteria group;n_Parcubacteria group;p_Candidatus Portnoybacteria; | 469 | 7 | 13 | 7 | 137 | 174 | 131 |
| k_Bacteria;p_Calditrichaeota; | 462 | 43 | 42 | 56 | 73 | 70 | 178 |
| k_Bacteria;n_FCB group;p_Candidatus Kryptonia; | 428 | 2 | 275 | 13 | 63 | 37 | 38 |
| k_Bacteria;n_unclassified Bacteria;n_Bacteria candidate phyla;n_Patescibacteria group;n_Parcubacteria group;p_Candidatus Wildermuthbacteria; | 403 | 7 | 9 | 13 | 132 | 155 | 87 |
| k_Bacteria;n_unclassified Bacteria;n_Bacteria candidate phyla;n_Patescibacteria group;n_Parcubacteria group;p_Candidatus Spechtbacteria; | 381 | 13 | 9 | 12 | 88 | 156 | 103 |
| k_Bacteria;n_unclassified Bacteria;n_Bacteria candidate phyla;n_Patescibacteria group;n_Parcubacteria group;p_Candidatus Andersenbacteria; | 371 | 8 | 11 | 10 | 103 | 155 | 84 |
| k_Bacteria;n_unclassified Bacteria;n_Bacteria candidate phyla;p_Candidatus Poribacteria; | 367 | 20 | 28 | 7 | 97 | 55 | 160 |
| k_Bacteria;n_Nitrospinae/Tectomicrobia group;p_Candidatus Tectomicrobia; | 319 | 7 | 8 | 10 | 66 | 48 | 180 |
| k_Bacteria;n_unclassified Bacteria;n_Bacteria candidate phyla;n_Patescibacteria group;n_Parcubacteria group;p_Candidatus Colwellbacteria; | 319 | 1 | 8 | 3 | 105 | 92 | 110 |
| k_Bacteria;n_Terrabacteria group;p_Candidatus Dormibacteraeota; | 299 | 7 | 3 | 7 | 67 | 89 | 126 |
| k_Bacteria;n_unclassified Bacteria;n_Bacteria candidate phyla;n_Patescibacteria group;n_Microgenomates group;p_Candidatus Cerribacteria; | 290 | 1 | 6 | 2 | 111 | 113 | 57 |
| k_Bacteria;n_unclassified Bacteria;n_Bacteria candidate phyla;n_Patescibacteria group;n_Parcubacteria group;p_Candidatus Kuenenbacteria; | 249 | 2 | 3 | 1 | 94 | 68 | 81 |
| k_Bacteria;n_unclassified Bacteria;n_Bacteria candidate phyla;n_Patescibacteria group;n_Parcubacteria group;p_Candidatus Niyogibacteria; | 242 | 0 | 5 | 1 | 83 | 85 | 68 |
| k_Bacteria;n_unclassified Bacteria;n_Bacteria candidate phyla;n_Patescibacteria group;n_Microgenomates group;p_Candidatus Blackburnbacteria; | 235 | 6 | 3 | 3 | 82 | 87 | 54 |
| k_Bacteria;n_unclassified Bacteria;n_Bacteria candidate phyla;p_Candidatus Wallbacteria; | 219 | 23 | 41 | 36 | 40 | 37 | 42 |
| k_Bacteria;p_Thermodesulfobacteria; | 214 | 14 | 30 | 18 | 40 | 75 | 37 |
| k_Bacteria;n_FCB group;n_Bacteroidetes/Chlorobi group;p_Candidatus Kapabacteria; | 211 | 17 | 21 | 11 | 64 | 43 | 55 |
| k_Bacteria;n_FCB group;n_Bacteroidetes/Chlorobi group;p_Balneolaeota; | 205 | 8 | 18 | 13 | 33 | 36 | 97 |
| k_Bacteria;n_FCB group;n_Bacteroidetes/Chlorobi group;p_Rhodothermaeota; | 205 | 11 | 9 | 4 | 73 | 43 | 65 |
| k_Bacteria;n_Caldiserica/Cryosericota group;p_Candidatus Cryosericota; | 199 | 19 | 21 | 37 | 25 | 27 | 70 |
| k_Bacteria;n_unclassified Bacteria;n_Bacteria candidate phyla;n_Patescibacteria group;n_Parcubacteria group;p_Candidatus Jacksonbacteria; | 193 | 0 | 4 | 2 | 39 | 75 | 73 |
| k_Bacteria;n_unclassified Bacteria;n_Bacteria candidate phyla;p_Candidatus Raymondbacteria; | 191 | 5 | 10 | 11 | 39 | 25 | 101 |
| k_Bacteria;n_unclassified Bacteria;n_Bacteria candidate phyla;n_Patescibacteria group;n_Parcubacteria group;p_Candidatus Terrybacteria; | 162 | 6 | 2 | 4 | 59 | 56 | 35 |
| k_Bacteria;n_unclassified Bacteria;n_Bacteria candidate phyla;p_Candidatus Desantisbacteria; | 159 | 16 | 8 | 11 | 41 | 43 | 40 |
| k_Bacteria;n_unclassified Bacteria;n_Bacteria candidate phyla;p_Candidatus Coatesbacteria; | 142 | 2 | 14 | 11 | 44 | 37 | 34 |
| k_Bacteria;n_unclassified Bacteria;n_Bacteria candidate phyla;p_Candidatus Firestonebacteria; | 136 | 2 | 15 | 7 | 27 | 62 | 23 |
| k_Bacteria;n_unclassified Bacteria;n_Bacteria candidate phyla;n_Patescibacteria group;n_Microgenomates group;p_Candidatus Chisholmbacteria; | 129 | 2 | 2 | 2 | 46 | 52 | 25 |
| k_Bacteria;n_unclassified Bacteria;n_Bacteria candidate phyla;p_Candidatus Goldbacteria; | 123 | 7 | 1 | 0 | 53 | 24 | 38 |
| k_Bacteria;n_unclassified Bacteria;n_Bacteria candidate phyla;n_Patescibacteria group;n_Parcubacteria group;p_Candidatus Tagabacteria; | 119 | 5 | 4 | 2 | 35 | 49 | 24 |
| k_Bacteria;n_PVC group;p_Candidatus Abyssubacteria; | 110 | 5 | 7 | 8 | 21 | 21 | 48 |
| k_Bacteria;n_unclassified Bacteria;n_Bacteria candidate phyla;p_Candidatus Lindowbacteria; | 100 | 4 | 5 | 14 | 21 | 12 | 44 |
| k_Bacteria;n_unclassified Bacteria;n_Bacteria candidate phyla;p_Candidatus Aerophobetes; | 97 | 0 | 1 | 5 | 30 | 30 | 31 |
| k_Bacteria;n_unclassified Bacteria;n_Bacteria candidate phyla;p_Candidatus Glassbacteria; | 96 | 19 | 11 | 18 | 20 | 7 | 21 |
| k_Bacteria;n_unclassified Bacteria;n_Bacteria candidate phyla;p_Candidatus Bipolaricaulota; | 88 | 7 | 11 | 10 | 14 | 12 | 34 |
| k_Bacteria;p_Chrysiogenetes; | 81 | 16 | 29 | 13 | 8 | 4 | 11 |
| k_Bacteria;n_unclassified Bacteria;n_Bacteria candidate phyla;p_candidate division NC10; | 77 | 8 | 16 | 8 | 9 | 14 | 22 |
| k_Bacteria;n_PVC group;p_Candidatus Aureabacteria; | 76 | 3 | 24 | 9 | 9 | 9 | 22 |
| k_Bacteria;n_PVC group;p_Kiritimatiellaeota; | 70 | 2 | 11 | 7 | 19 | 11 | 20 |
| k_Bacteria;n_Terrabacteria group;p_Abditibacteriota; | 66 | 2 | 4 | 11 | 19 | 6 | 24 |
| k_Bacteria;n_unclassified Bacteria;n_Bacteria candidate phyla;n_Patescibacteria group;n_Parcubacteria group;p_Candidatus Brennerbacteria; | 58 | 1 | 2 | 3 | 15 | 16 | 21 |
| k_Bacteria;n_unclassified Bacteria;n_Bacteria candidate phyla;p_Candidatus Fischerbacteria; | 58 | 1 | 7 | 6 | 9 | 10 | 25 |
| k_Bacteria;n_Caldiserica/Cryosericota group;p_Caldiserica; | 57 | 3 | 9 | 8 | 12 | 15 | 10 |
| k_Bacteria;n_unclassified Bacteria;n_Bacteria candidate phyla;p_Candidatus Delongbacteria; | 38 | 4 | 12 | 3 | 7 | 9 | 3 |
| k_Bacteria;n_unclassified Bacteria;n_Bacteria candidate phyla;p_Candidatus Fraserbacteria; | 37 | 0 | 0 | 1 | 17 | 11 | 8 |
| k_Bacteria;n_unclassified Bacteria;n_Bacteria candidate phyla;p_Candidatus Wirthbacteria; | 28 | 1 | 0 | 2 | 7 | 7 | 11 |
| k_Bacteria;n_unclassified Bacteria;n_Bacteria candidate phyla;p_Candidatus Hydrothermae; | 25 | 0 | 0 | 2 | 12 | 6 | 5 |
| k_Bacteria;n_unclassified Bacteria;n_Bacteria candidate phyla;p_Candidatus Edwardsbacteria; | 24 | 0 | 1 | 1 | 10 | 5 | 7 |
| k_Bacteria;p_Coprothermobacterota; | 21 | 4 | 8 | 2 | 3 | 1 | 3 |
| k_Bacteria;p_Dictyoglomi; | 21 | 1 | 2 | 2 | 6 | 3 | 7 |
| k_Bacteria;n_Terrabacteria group;p_Candidatus Eremiobacteraeota; | 16 | 2 | 1 | 1 | 4 | 2 | 6 |
| k_Bacteria;n_unclassified Bacteria;n_Bacteria candidate phyla;n_Patescibacteria group;n_Parcubacteria group;p_Candidatus Veblenbacteria; | 10 | 0 | 0 | 0 | 2 | 3 | 5 |
| **Total** | **21911457** | **5034849** | **6143390** | **4855733** | **1732473** | **1605647** | **2539365** |

**Table S5 Number of reads for each bacterial Class**

| **Class** | **Total** | **RAW1** | **RAW2** | **RAW3** | **UV1** | **UV2** | **UV3** |
| --- | --- | --- | --- | --- | --- | --- | --- |
| k_Bacteria;p_Proteobacteria;c_Gammaproteobacteria; | 3940536 | 1243296 | 1256075 | 1115087 | 114631 | 85405 | 126042 |
| k_Bacteria;p_Proteobacteria;n_delta/epsilon subdivisions;c_Epsilonproteobacteria; | 3345259 | 960603 | 1414548 | 933576 | 15951 | 14285 | 6296 |
| k_Bacteria;n_FCB group;n_Bacteroidetes/Chlorobi group;p_Bacteroidetes;c_Bacteroidia; | 2701648 | 878200 | 1023845 | 719552 | 38333 | 30969 | 10749 |
| k_Bacteria;p_Proteobacteria;c_Betaproteobacteria; | 3839734 | 872073 | 1044584 | 855265 | 247785 | 271382 | 548645 |
| k_Bacteria;n_Terrabacteria group;p_Firmicutes;c_Clostridia; | 833652 | 250669 | 267512 | 279382 | 15565 | 12820 | 7704 |
| k_Bacteria;n_FCB group;n_Bacteroidetes/Chlorobi group;p_Bacteroidetes;c_Flavobacteriia; | 412090 | 111859 | 125797 | 95737 | 16108 | 51154 | 11435 |
| k_Bacteria;p_Proteobacteria;c_Alphaproteobacteria; | 678131 | 74352 | 92219 | 83760 | 140549 | 115280 | 171971 |
| k_Bacteria;n_Terrabacteria group;p_Actinobacteria;c_Actinobacteria; | 720023 | 50292 | 42684 | 57207 | 165609 | 113144 | 291087 |
| k_Bacteria;p_Proteobacteria;n_delta/epsilon subdivisions;c_Deltaproteobacteria; | 355013 | 40850 | 91763 | 52975 | 45710 | 34480 | 89235 |
| k_Bacteria;n_Terrabacteria group;p_Firmicutes;c_Negativicutes; | 125670 | 35008 | 44056 | 41292 | 2295 | 2105 | 914 |
| k_Bacteria;n_Terrabacteria group;p_Firmicutes;c_Bacilli; | 152459 | 32845 | 46919 | 40845 | 10558 | 11630 | 9662 |
| k_Bacteria;p_Fusobacteria;c_Fusobacteriia; | 53711 | 11844 | 24953 | 15712 | 476 | 493 | 233 |
| k_Bacteria;n_Terrabacteria group;p_Actinobacteria;c_Coriobacteriia; | 31708 | 9854 | 9674 | 10359 | 904 | 582 | 335 |
| k_Bacteria;n_Terrabacteria group;p_Firmicutes;c_Erysipelotrichia; | 11867 | 4058 | 3521 | 3647 | 192 | 371 | 78 |
| k_Bacteria;n_PVC group;p_Verrucomicrobia;c_Verrucomicrobiae; | 19866 | 3528 | 4570 | 4468 | 2755 | 2006 | 2539 |
| k_Bacteria;n_FCB group;n_Bacteroidetes/Chlorobi group;p_Bacteroidetes;c_Chitinophagia; | 44154 | 2476 | 2503 | 2087 | 6286 | 21236 | 9566 |
| k_Bacteria;p_Synergistetes;c_Synergistia; | 12956 | 2053 | 7364 | 2602 | 354 | 397 | 186 |
| k_Bacteria;n_FCB group;n_Bacteroidetes/Chlorobi group;p_Bacteroidetes;c_Sphingobacteriia; | 69862 | 1985 | 2132 | 1179 | 41771 | 15992 | 6803 |
| k_Bacteria;n_PVC group;p_Verrucomicrobia;c_Opitutae; | 24554 | 1775 | 6230 | 4690 | 4072 | 2540 | 5247 |
| k_Bacteria;p_Proteobacteria;c_Oligoflexia; | 53746 | 1609 | 3106 | 2552 | 21503 | 12387 | 12589 |
| k_Bacteria;n_Terrabacteria group;p_Actinobacteria;c_Acidimicrobiia; | 17533 | 1517 | 325 | 608 | 5416 | 3000 | 6667 |
| k_Bacteria;n_PVC group;p_Chlamydiae;c_Chlamydiia; | 12225 | 1489 | 1767 | 1740 | 2378 | 2293 | 2558 |
| k_Bacteria;p_Spirochaetes;c_Spirochaetia; | 17982 | 1437 | 1952 | 1651 | 3873 | 2033 | 7036 |
| k_Bacteria;p_Acidobacteria;c_Holophagae; | 10539 | 1340 | 4614 | 2337 | 548 | 364 | 1336 |
| k_Bacteria;n_PVC group;p_Lentisphaerae;c_Lentisphaeria; | 8632 | 1298 | 2693 | 3265 | 818 | 373 | 185 |
| k_Bacteria;p_Nitrospirae;c_Nitrospira; | 42481 | 1240 | 282 | 595 | 11966 | 6969 | 21429 |
| k_Bacteria;n_FCB group;n_Bacteroidetes/Chlorobi group;p_Bacteroidetes;c_Cytophagia; | 15376 | 1165 | 1358 | 1185 | 3466 | 2842 | 5360 |
| k_Bacteria;n_Terrabacteria group;p_Chloroflexi;c_Ardenticatenia; | 104753 | 1045 | 340 | 7699 | 3583 | 3677 | 88409 |
| k_Bacteria;n_Terrabacteria group;p_Actinobacteria;c_Thermoleophilia; | 25480 | 953 | 385 | 1216 | 7086 | 3874 | 11966 |
| k_Bacteria;n_FCB group;p_Fibrobacteres;c_Fibrobacteria; | 3188 | 801 | 1098 | 792 | 163 | 252 | 82 |
| k_Bacteria;n_Terrabacteria group;p_Chloroflexi;c_Chloroflexia; | 10396 | 799 | 119 | 182 | 2960 | 1577 | 4759 |
| k_Bacteria;n_PVC group;p_Planctomycetes;c_Planctomycetia; | 19302 | 708 | 662 | 690 | 6578 | 4234 | 6430 |
| k_Bacteria;n_Terrabacteria group;p_Chloroflexi;c_Anaerolineae; | 25632 | 645 | 398 | 831 | 3928 | 3048 | 16782 |
| k_Bacteria;n_Terrabacteria group;p_Tenericutes;c_Mollicutes; | 2060 | 582 | 606 | 468 | 136 | 189 | 79 |
| k_Bacteria;p_Proteobacteria;c_Hydrogenophilalia; | 4055 | 492 | 727 | 624 | 341 | 546 | 1325 |
| k_Bacteria;n_Terrabacteria group;p_Firmicutes;c_Tissierellia; | 1851 | 387 | 742 | 520 | 77 | 74 | 51 |
| k_Bacteria;p_Deferribacteres;c_Deferribacteres; | 3986 | 383 | 2239 | 1248 | 37 | 51 | 28 |
| k_Bacteria;p_Elusimicrobia;c_Elusimicrobia; | 2855 | 303 | 1711 | 712 | 55 | 59 | 15 |
| k_Bacteria;p_Aquificae;c_Aquificae; | 1330 | 267 | 317 | 313 | 170 | 126 | 137 |
| k_Bacteria;n_Terrabacteria group;p_Chloroflexi;c_Dehalococcoidia; | 6032 | 250 | 279 | 331 | 1332 | 1304 | 2536 |
| k_Bacteria;p_Proteobacteria;c_Acidithiobacillia; | 2579 | 249 | 606 | 513 | 327 | 419 | 465 |
| k_Bacteria;n_Terrabacteria group;p_Deinococcus-Thermus;c_Deinococci; | 3223 | 245 | 211 | 155 | 819 | 900 | 893 |
| k_Bacteria;n_Terrabacteria group;p_Chloroflexi;c_Caldilineae; | 3005 | 225 | 43 | 163 | 360 | 170 | 2044 |
| k_Bacteria;p_Thermotogae;c_Thermotogae; | 1568 | 189 | 243 | 205 | 270 | 509 | 152 |
| k_Bacteria;p_Acidobacteria;c_Acidobacteriia; | 4642 | 130 | 190 | 186 | 1107 | 708 | 2321 |
| k_Bacteria;n_FCB group;n_Bacteroidetes/Chlorobi group;p_Ignavibacteriae;c_Ignavibacteria; | 3011 | 126 | 144 | 154 | 620 | 409 | 1558 |
| k_Bacteria;n_FCB group;n_Bacteroidetes/Chlorobi group;p_Chlorobi;c_Chlorobia; | 949 | 103 | 174 | 152 | 150 | 182 | 188 |
| k_Bacteria;n_FCB group;n_Bacteroidetes/Chlorobi group;p_Bacteroidetes;c_Saprospiria; | 2078 | 86 | 52 | 44 | 467 | 225 | 1204 |
| k_Bacteria;n_FCB group;p_Gemmatimonadetes;c_Gemmatimonadetes; | 1348 | 75 | 21 | 28 | 283 | 156 | 785 |
| k_Bacteria;n_PVC group;p_Verrucomicrobia;c_Spartobacteria; | 984 | 73 | 122 | 76 | 277 | 255 | 181 |
| k_Bacteria;n_unclassified Bacteria;n_Bacteria candidate phyla;p_Candidatus Peregrinibacteria;c_Candidatus Peribacteria; | 11385 | 69 | 306 | 191 | 2311 | 5157 | 3351 |
| k_Bacteria;p_Proteobacteria;c_Zetaproteobacteria; | 889 | 46 | 57 | 58 | 285 | 210 | 233 |
| k_Bacteria;n_Terrabacteria group;p_Chloroflexi;c_Ktedonobacteria; | 826 | 43 | 34 | 28 | 228 | 182 | 311 |
| k_Bacteria;n_PVC group;p_Planctomycetes;c_Phycisphaerae; | 931 | 36 | 41 | 59 | 238 | 136 | 421 |
| k_Bacteria;n_Terrabacteria group;p_Chloroflexi;c_Candidatus Thermofonsia; | 1044 | 31 | 24 | 23 | 255 | 229 | 482 |
| k_Bacteria;n_PVC group;p_Planctomycetes;c_Candidatus Brocadiae; | 607 | 30 | 36 | 48 | 149 | 120 | 224 |
| k_Bacteria;n_Terrabacteria group;p_Chloroflexi;c_Thermoflexia; | 515 | 29 | 12 | 15 | 75 | 47 | 337 |
| k_Bacteria;p_Acidobacteria;c_Blastocatellia; | 663 | 19 | 13 | 12 | 150 | 105 | 364 |
| k_Bacteria;n_Caldiserica/Cryosericota group;p_Candidatus Cryosericota;c_Candidatus Cryosericia; | 176 | 16 | 20 | 35 | 23 | 20 | 62 |
| k_Bacteria;n_Terrabacteria group;p_Armatimonadetes;c_Fimbriimonadia; | 199 | 13 | 3 | 4 | 82 | 25 | 72 |
| k_Bacteria;n_Terrabacteria group;p_Armatimonadetes;c_Armatimonadia; | 486 | 12 | 10 | 11 | 204 | 112 | 137 |
| k_Bacteria;n_FCB group;n_Bacteroidetes/Chlorobi group;p_Rhodothermaeota;c_Rhodothermia; | 133 | 10 | 7 | 2 | 57 | 21 | 36 |
| k_Bacteria;n_Terrabacteria group;n_Cyanobacteria/Melainabacteria group;p_Candidatus Margulisbacteria;c_Candidatus Termititenacia; | 158 | 10 | 21 | 8 | 46 | 51 | 22 |
| k_Bacteria;n_unclassified Bacteria;n_Bacteria candidate phyla;p_Candidatus Riflebacteria;c_Candidatus Ozemobacteria; | 88 | 10 | 8 | 13 | 21 | 18 | 18 |
| k_Bacteria;p_Calditrichaeota;c_Calditrichae; | 97 | 10 | 7 | 7 | 19 | 20 | 34 |
| k_Bacteria;p_Chrysiogenetes;c_Chrysiogenetes; | 63 | 9 | 24 | 10 | 7 | 4 | 9 |
| k_Bacteria;p_Thermodesulfobacteria;c_Thermodesulfobacteria; | 143 | 9 | 19 | 16 | 23 | 47 | 29 |
| k_Bacteria;n_Terrabacteria group;p_Actinobacteria;c_Nitriliruptoria; | 274 | 8 | 3 | 16 | 84 | 56 | 107 |
| k_Bacteria;p_Elusimicrobia;c_Endomicrobia; | 99 | 8 | 43 | 31 | 6 | 6 | 5 |
| k_Bacteria;n_Terrabacteria group;p_Chloroflexi;c_Thermomicrobia; | 325 | 7 | 11 | 9 | 58 | 129 | 111 |
| k_Bacteria;n_FCB group;n_Bacteroidetes/Chlorobi group;p_Balneolaeota;c_Balneolia; | 154 | 6 | 12 | 9 | 26 | 26 | 75 |
| k_Bacteria;n_FCB group;p_Fibrobacteres;c_Chitinivibrionia; | 29 | 6 | 12 | 6 | 2 | 1 | 2 |
| k_Bacteria;n_Terrabacteria group;p_Firmicutes;c_Limnochordia; | 18 | 6 | 0 | 1 | 0 | 0 | 11 |
| k_Bacteria;n_unclassified Bacteria;n_Bacteria candidate phyla;n_Candidatus Dependentiae;c_Candidatus Babeliae; | 205 | 6 | 3 | 2 | 113 | 56 | 25 |
| k_Bacteria;n_Terrabacteria group;p_Actinobacteria;c_Rubrobacteria; | 295 | 5 | 24 | 11 | 76 | 89 | 90 |
| k_Bacteria;n_Terrabacteria group;p_Armatimonadetes;c_Chthonomonadetes; | 65 | 5 | 3 | 2 | 22 | 6 | 27 |
| k_Bacteria;n_PVC group;p_Verrucomicrobia;c_Methylacidiphilae; | 31 | 4 | 5 | 3 | 4 | 3 | 12 |
| k_Bacteria;n_Terrabacteria group;n_Cyanobacteria/Melainabacteria group;p_Cyanobacteria;c_Gloeobacteria; | 69 | 4 | 5 | 8 | 19 | 15 | 18 |
| k_Bacteria;p_Proteobacteria;c_Candidatus Muproteobacteria; | 76 | 4 | 8 | 14 | 8 | 14 | 28 |
| k_Bacteria;n_FCB group;p_Candidatus Fermentibacteria;c_Candidatus Fermentibacteria (class); | 21 | 3 | 5 | 1 | 9 | 2 | 1 |
| k_Bacteria;n_FCB group;p_Fibrobacteres;c_Chitinispirillia; | 51 | 3 | 6 | 0 | 18 | 11 | 13 |
| k_Bacteria;n_PVC group;p_Kiritimatiellaeota;c_Kiritimatiellae; | 59 | 3 | 8 | 5 | 18 | 9 | 16 |
| k_Bacteria;n_Terrabacteria group;n_Cyanobacteria/Melainabacteria group;p_Candidatus Margulisbacteria;c_Candidatus Riflemargulisbacteria; | 71 | 3 | 11 | 5 | 26 | 18 | 8 |
| k_Bacteria;n_unclassified Bacteria;n_Bacteria candidate phyla;p_Candidatus Sumerlaeota;c_Candidatus Sumerlaeia; | 38 | 3 | 3 | 2 | 13 | 3 | 14 |
| k_Bacteria;p_Dictyoglomi;c_Dictyoglomia; | 24 | 3 | 0 | 4 | 7 | 3 | 7 |
| k_Bacteria;n_Nitrospinae/Tectomicrobia group;p_Nitrospinae;c_Nitrospinia; | 48 | 2 | 4 | 2 | 14 | 14 | 12 |
| k_Bacteria;n_Terrabacteria group;p_Abditibacteriota;c_Abditibacteria; | 42 | 2 | 2 | 10 | 11 | 5 | 12 |
| k_Bacteria;p_Coprothermobacterota;c_Coprothermobacteria; | 15 | 2 | 7 | 1 | 2 | 0 | 3 |
| k_Bacteria;n_Caldiserica/Cryosericota group;p_Caldiserica;c_Caldisericia; | 20 | 1 | 2 | 3 | 3 | 5 | 6 |
| k_Bacteria;p_Acidobacteria;c_Vicinamibacteria; | 146 | 1 | 9 | 3 | 35 | 19 | 79 |
| k_Bacteria;n_Terrabacteria group;n_Cyanobacteria/Melainabacteria group;p_Candidatus Margulisbacteria;c_Candidatus Marinamargulisbacteria; | 276 | 0 | 0 | 3 | 94 | 73 | 106 |
| k_Bacteria;p_Acidobacteria;c_Thermoanaerobaculia; | 12 | 0 | 1 | 0 | 0 | 0 | 11 |
| k_Bacteria;p_Proteobacteria;c_Candidatus Lambdaproteobacteria; | 30 | 0 | 3 | 2 | 5 | 12 | 8 |
| **Total** | **17806610** | **4608328** | **5539307** | **4351350** | **955844** | **846555** | **1505226** |

**Table S6 Number of reads for each bacterial Order**

| **Order** | **Total** | **RAW1** | **RAW2** | **RAW3** | **UV1** | **UV2** | **UV3** |
| --- | --- | --- | --- | --- | --- | --- | --- |
| k_Bacteria;p_Proteobacteria;n_delta/epsilon subdivisions;c_Epsilonproteobacteria;o_Campylobacterales; | 3289459 | 946069 | 1390594 | 918081 | 15243 | 13605 | 5867 |
| k_Bacteria;n_FCB group;n_Bacteroidetes/Chlorobi group;p_Bacteroidetes;c_Bacteroidia;o_Bacteroidales; | 2602078 | 855341 | 979941 | 693137 | 36044 | 28458 | 9157 |
| k_Bacteria;p_Proteobacteria;c_Betaproteobacteria;o_Burkholderiales; | 2250963 | 566405 | 631727 | 516337 | 136411 | 148037 | 252046 |
| k_Bacteria;p_Proteobacteria;c_Gammaproteobacteria;o_Aeromonadales; | 1413862 | 451921 | 466197 | 471609 | 12525 | 7322 | 4288 |
| k_Bacteria;p_Proteobacteria;c_Gammaproteobacteria;o_Pseudomonadales; | 1342232 | 475173 | 453153 | 362125 | 19138 | 16022 | 16621 |
| k_Bacteria;n_Terrabacteria group;p_Firmicutes;c_Clostridia;o_Clostridiales; | 775498 | 236679 | 249728 | 260874 | 12686 | 9916 | 5615 |
| k_Bacteria;p_Proteobacteria;c_Gammaproteobacteria;o_Enterobacterales; | 437271 | 144808 | 145105 | 116981 | 14501 | 8695 | 7181 |
| k_Bacteria;p_Proteobacteria;c_Betaproteobacteria;o_Neisseriales; | 424382 | 123591 | 141575 | 117222 | 7599 | 21289 | 13106 |
| k_Bacteria;n_FCB group;n_Bacteroidetes/Chlorobi group;p_Bacteroidetes;c_Flavobacteriia;o_Flavobacteriales; | 362054 | 102056 | 117565 | 86422 | 13013 | 33917 | 9081 |
| k_Bacteria;n_Terrabacteria group;p_Actinobacteria;c_Actinobacteria;o_Corynebacteriales; | 304166 | 13345 | 7455 | 18221 | 64225 | 30827 | 170093 |
| k_Bacteria;p_Proteobacteria;c_Betaproteobacteria;o_Rhodocyclales; | 208977 | 46697 | 56263 | 53392 | 11203 | 14543 | 26879 |
| k_Bacteria;p_Proteobacteria;c_Alphaproteobacteria;o_Rhizobiales; | 162154 | 13863 | 15588 | 14650 | 37519 | 28846 | 51688 |
| k_Bacteria;p_Proteobacteria;c_Gammaproteobacteria;o_Xanthomonadales; | 115312 | 25842 | 25855 | 23879 | 10369 | 9618 | 19749 |
| k_Bacteria;n_Terrabacteria group;p_Firmicutes;c_Bacilli;o_Lactobacillales; | 103325 | 26209 | 36394 | 32046 | 2559 | 3351 | 2766 |
| k_Bacteria;n_Terrabacteria group;p_Chloroflexi;c_Ardenticatenia;o_Ardenticatenales; | 101012 | 976 | 303 | 7494 | 3195 | 3309 | 85735 |
| k_Bacteria;p_Proteobacteria;n_delta/epsilon subdivisions;c_Deltaproteobacteria;o_Desulfovibrionales; | 99815 | 17218 | 51822 | 25280 | 1837 | 1891 | 1767 |
| k_Bacteria;p_Proteobacteria;c_Alphaproteobacteria;o_Rhodospirillales; | 98810 | 16643 | 26569 | 22953 | 11716 | 8943 | 11986 |
| k_Bacteria;p_Proteobacteria;n_delta/epsilon subdivisions;c_Deltaproteobacteria;o_Myxococcales; | 86942 | 1256 | 727 | 857 | 20761 | 12803 | 50538 |
| k_Bacteria;n_Terrabacteria group;p_Firmicutes;c_Negativicutes;o_Selenomonadales; | 62400 | 17486 | 22672 | 19889 | 979 | 999 | 375 |
| k_Bacteria;n_FCB group;n_Bacteroidetes/Chlorobi group;p_Bacteroidetes;c_Sphingobacteriia;o_Sphingobacteriales; | 61128 | 1731 | 1858 | 1012 | 36988 | 13830 | 5709 |
| k_Bacteria;p_Proteobacteria;c_Alphaproteobacteria;o_Rhodobacterales; | 57859 | 12194 | 11082 | 12635 | 6847 | 7372 | 7729 |
| k_Bacteria;p_Proteobacteria;c_Alphaproteobacteria;o_Sphingomonadales; | 57297 | 5839 | 6118 | 5430 | 8779 | 10526 | 20605 |
| k_Bacteria;p_Fusobacteria;c_Fusobacteriia;o_Fusobacteriales; | 47304 | 9979 | 22575 | 13758 | 372 | 431 | 189 |
| k_Bacteria;p_Proteobacteria;c_Gammaproteobacteria;o_Alteromonadales; | 46692 | 12846 | 14922 | 14317 | 1577 | 1639 | 1391 |
| k_Bacteria;n_Terrabacteria group;p_Actinobacteria;c_Actinobacteria;o_Micrococcales; | 45486 | 6522 | 5384 | 7020 | 7406 | 4268 | 14886 |
| k_Bacteria;n_Terrabacteria group;p_Actinobacteria;c_Actinobacteria;o_Bifidobacteriales; | 39686 | 12583 | 12407 | 12653 | 793 | 615 | 635 |
| k_Bacteria;n_FCB group;n_Bacteroidetes/Chlorobi group;p_Bacteroidetes;c_Chitinophagia;o_Chitinophagales; | 39140 | 2183 | 2181 | 1851 | 5142 | 19392 | 8391 |
| k_Bacteria;p_Proteobacteria;c_Betaproteobacteria;o_Nitrosomonadales; | 35892 | 2987 | 4562 | 3597 | 7201 | 6324 | 11221 |
| k_Bacteria;p_Proteobacteria;c_Oligoflexia;o_Bdellovibrionales; | 34590 | 536 | 764 | 639 | 15357 | 8333 | 8961 |
| k_Bacteria;n_Terrabacteria group;p_Firmicutes;c_Bacilli;o_Bacillales; | 28404 | 3795 | 5852 | 4721 | 4857 | 4913 | 4266 |
| k_Bacteria;p_Proteobacteria;c_Alphaproteobacteria;o_Caulobacterales; | 27292 | 5738 | 5942 | 4922 | 2786 | 1994 | 5910 |
| k_Bacteria;p_Proteobacteria;n_delta/epsilon subdivisions;c_Deltaproteobacteria;o_Desulfobacterales; | 24395 | 3738 | 12229 | 5628 | 932 | 929 | 939 |
| k_Bacteria;n_Terrabacteria group;p_Firmicutes;c_Negativicutes;o_Veillonellales; | 24247 | 6707 | 8532 | 8088 | 409 | 328 | 183 |
| k_Bacteria;n_Terrabacteria group;p_Actinobacteria;c_Thermoleophilia;o_Solirubrobacterales; | 22649 | 845 | 339 | 1085 | 6278 | 3429 | 10673 |
| k_Bacteria;p_Proteobacteria;c_Gammaproteobacteria;o_Vibrionales; | 22157 | 6335 | 8159 | 5951 | 673 | 492 | 547 |
| k_Bacteria;n_Terrabacteria group;p_Actinobacteria;c_Actinobacteria;o_Propionibacteriales; | 21767 | 3344 | 2973 | 2747 | 4374 | 2234 | 6095 |
| k_Bacteria;n_Terrabacteria group;p_Firmicutes;c_Negativicutes;o_Acidaminococcales; | 21447 | 6403 | 6758 | 7176 | 546 | 397 | 167 |
| k_Bacteria;n_Terrabacteria group;p_Actinobacteria;c_Coriobacteriia;o_Coriobacteriales; | 21229 | 6668 | 6445 | 7065 | 534 | 319 | 198 |
| k_Bacteria;p_Nitrospirae;c_Nitrospira;o_Nitrospirales; | 18612 | 570 | 170 | 277 | 5256 | 3129 | 9210 |
| k_Bacteria;n_PVC group;p_Verrucomicrobia;c_Verrucomicrobiae;o_Verrucomicrobiales; | 17432 | 3419 | 4285 | 4284 | 2036 | 1469 | 1939 |
| k_Bacteria;n_Terrabacteria group;p_Actinobacteria;c_Actinobacteria;o_Streptomycetales; | 16556 | 572 | 686 | 724 | 4916 | 4164 | 5494 |
| k_Bacteria;p_Proteobacteria;c_Gammaproteobacteria;o_Chromatiales; | 14110 | 4172 | 1811 | 5078 | 799 | 603 | 1647 |
| k_Bacteria;n_PVC group;p_Planctomycetes;c_Planctomycetia;o_Planctomycetales; | 13941 | 541 | 512 | 519 | 4658 | 3020 | 4691 |
| k_Bacteria;p_Proteobacteria;n_delta/epsilon subdivisions;c_Deltaproteobacteria;o_Desulfuromonadales; | 12521 | 1883 | 5672 | 3358 | 565 | 420 | 623 |
| k_Bacteria;n_FCB group;n_Bacteroidetes/Chlorobi group;p_Bacteroidetes;c_Cytophagia;o_Cytophagales; | 12474 | 1003 | 1143 | 1011 | 2744 | 2249 | 4324 |
| k_Bacteria;p_Synergistetes;c_Synergistia;o_Synergistales; | 11669 | 1862 | 6637 | 2353 | 320 | 335 | 162 |
| k_Bacteria;n_Terrabacteria group;p_Firmicutes;c_Erysipelotrichia;o_Erysipelotrichales; | 11461 | 3971 | 3436 | 3531 | 165 | 290 | 68 |
| k_Bacteria;p_Spirochaetes;c_Spirochaetia;o_Leptospirales; | 10854 | 545 | 417 | 429 | 2487 | 1275 | 5701 |
| k_Bacteria;n_PVC group;p_Verrucomicrobia;c_Opitutae;o_Opitutales; | 10313 | 618 | 2299 | 1730 | 2186 | 1212 | 2268 |
| k_Bacteria;p_Proteobacteria;n_delta/epsilon subdivisions;c_Deltaproteobacteria;o_Syntrophobacterales; | 9201 | 2136 | 1472 | 2003 | 1477 | 1140 | 973 |
| k_Bacteria;p_Acidobacteria;c_Holophagae;o_Holophagales; | 9138 | 1205 | 4118 | 2086 | 423 | 302 | 1004 |
| k_Bacteria;p_Proteobacteria;c_Gammaproteobacteria;o_Oceanospirillales; | 8452 | 1387 | 1996 | 1543 | 1232 | 990 | 1304 |
| k_Bacteria;n_PVC group;p_Lentisphaerae;c_Lentisphaeria;o_Victivallales; | 8153 | 1233 | 2550 | 3109 | 761 | 334 | 166 |
| k_Bacteria;n_Terrabacteria group;p_Chloroflexi;c_Chloroflexia;o_Chloroflexales; | 7493 | 688 | 66 | 133 | 1938 | 943 | 3725 |
| k_Bacteria;p_Proteobacteria;c_Gammaproteobacteria;o_Legionellales; | 7165 | 647 | 606 | 497 | 2484 | 1805 | 1126 |
| k_Bacteria;n_Terrabacteria group;p_Chloroflexi;c_Anaerolineae;o_Anaerolineales; | 7112 | 242 | 172 | 164 | 1668 | 1161 | 3705 |
| k_Bacteria;p_Proteobacteria;c_Gammaproteobacteria;o_Methylococcales; | 7082 | 635 | 974 | 837 | 1542 | 1136 | 1958 |
| k_Bacteria;p_Proteobacteria;c_Gammaproteobacteria;o_Pasteurellales; | 6910 | 1696 | 2238 | 1904 | 386 | 377 | 309 |
| k_Bacteria;n_Terrabacteria group;p_Actinobacteria;c_Coriobacteriia;o_Eggerthellales; | 6096 | 1914 | 1852 | 1912 | 219 | 159 | 40 |
| k_Bacteria;p_Proteobacteria;c_Alphaproteobacteria;o_Rickettsiales; | 5968 | 120 | 147 | 116 | 2333 | 1990 | 1262 |
| k_Bacteria;n_FCB group;n_Bacteroidetes/Chlorobi group;p_Bacteroidetes;c_Bacteroidia;o_Marinilabiliales; | 5814 | 777 | 2618 | 1759 | 242 | 157 | 261 |
| k_Bacteria;n_Terrabacteria group;n_Cyanobacteria/Melainabacteria group;p_Cyanobacteria;o_Nostocales; | 5762 | 198 | 241 | 253 | 1613 | 1174 | 2283 |
| k_Bacteria;n_Terrabacteria group;p_Actinobacteria;c_Acidimicrobiia;o_Acidimicrobiales; | 5613 | 880 | 78 | 297 | 1267 | 284 | 2807 |
| k_Bacteria;n_Terrabacteria group;p_Actinobacteria;c_Actinobacteria;o_Actinomycetales; | 4872 | 1109 | 1479 | 1232 | 359 | 220 | 473 |
| k_Bacteria;p_Proteobacteria;c_Gammaproteobacteria;o_Thiotrichales; | 4825 | 853 | 1804 | 951 | 415 | 397 | 405 |
| k_Bacteria;n_PVC group;p_Chlamydiae;c_Chlamydiia;o_Chlamydiales; | 4620 | 1137 | 1349 | 1314 | 225 | 260 | 335 |
| k_Bacteria;n_Terrabacteria group;p_Actinobacteria;c_Actinobacteria;o_Micromonosporales; | 4345 | 109 | 128 | 211 | 1219 | 1069 | 1609 |
| k_Bacteria;p_Spirochaetes;c_Spirochaetia;o_Spirochaetales; | 4094 | 541 | 1044 | 855 | 715 | 406 | 533 |
| k_Bacteria;n_Terrabacteria group;p_Actinobacteria;c_Actinobacteria;o_Pseudonocardiales; | 3825 | 105 | 122 | 130 | 1156 | 1041 | 1271 |
| k_Bacteria;p_Deferribacteres;c_Deferribacteres;o_Deferribacterales; | 3815 | 361 | 2152 | 1210 | 30 | 41 | 21 |
| k_Bacteria;n_PVC group;p_Chlamydiae;c_Chlamydiia;o_Parachlamydiales; | 3782 | 120 | 130 | 146 | 1137 | 1041 | 1208 |
| k_Bacteria;p_Proteobacteria;c_Hydrogenophilalia;o_Hydrogenophilales; | 3710 | 473 | 666 | 594 | 312 | 426 | 1239 |
| k_Bacteria;n_Terrabacteria group;p_Actinobacteria;c_Actinobacteria;o_Streptosporangiales; | 3222 | 79 | 80 | 108 | 1079 | 746 | 1130 |
| k_Bacteria;p_Elusimicrobia;c_Elusimicrobia;o_Elusimicrobiales; | 2855 | 303 | 1711 | 712 | 55 | 59 | 15 |
| k_Bacteria;p_Proteobacteria;c_Gammaproteobacteria;o_Cellvibrionales; | 2723 | 479 | 276 | 449 | 582 | 367 | 570 |
| k_Bacteria;n_FCB group;p_Fibrobacteres;c_Fibrobacteria;o_Fibrobacterales; | 2696 | 680 | 938 | 668 | 134 | 210 | 66 |
| k_Bacteria;p_Proteobacteria;c_Oligoflexia;o_Bacteriovoracales; | 2425 | 360 | 419 | 388 | 638 | 409 | 211 |
| k_Bacteria;p_Proteobacteria;c_Alphaproteobacteria;o_Holosporales; | 2220 | 103 | 78 | 93 | 842 | 813 | 291 |
| k_Bacteria;p_Proteobacteria;c_Acidithiobacillia;o_Acidithiobacillales; | 2169 | 230 | 568 | 481 | 244 | 295 | 351 |
| k_Bacteria;n_Terrabacteria group;n_Cyanobacteria/Melainabacteria group;p_Cyanobacteria;o_Synechococcales; | 1928 | 127 | 151 | 129 | 502 | 429 | 590 |
| k_Bacteria;n_Terrabacteria group;p_Chloroflexi;c_Caldilineae;o_Caldilineales; | 1837 | 164 | 27 | 99 | 241 | 86 | 1220 |
| k_Bacteria;p_Proteobacteria;c_Gammaproteobacteria;o_Nevskiales; | 1835 | 81 | 79 | 106 | 208 | 160 | 1201 |
| k_Bacteria;n_Terrabacteria group;n_Cyanobacteria/Melainabacteria group;p_Candidatus Melainabacteria;o_Candidatus Gastranaerophilales; | 1825 | 479 | 637 | 508 | 127 | 44 | 30 |
| k_Bacteria;n_FCB group;n_Bacteroidetes/Chlorobi group;p_Bacteroidetes;c_Saprospiria;o_Saprospirales; | 1708 | 64 | 40 | 30 | 378 | 178 | 1018 |
| k_Bacteria;p_Acidobacteria;c_Acidobacteriia;o_Acidobacteriales; | 1708 | 56 | 109 | 96 | 476 | 354 | 617 |
| k_Bacteria;p_Proteobacteria;c_Alphaproteobacteria;o_Pelagibacterales; | 1592 | 22 | 65 | 51 | 598 | 538 | 318 |
| k_Bacteria;n_Terrabacteria group;p_Actinobacteria;c_Actinobacteria;o_Frankiales; | 1537 | 43 | 44 | 55 | 441 | 395 | 559 |
| k_Bacteria;n_Terrabacteria group;p_Tenericutes;c_Mollicutes;o_Mycoplasmatales; | 1468 | 428 | 519 | 384 | 61 | 43 | 33 |
| k_Bacteria;n_Terrabacteria group;p_Firmicutes;c_Tissierellia;o_Tissierellales; | 1441 | 310 | 571 | 423 | 52 | 48 | 37 |
| k_Bacteria;n_Terrabacteria group;n_Cyanobacteria/Melainabacteria group;p_Cyanobacteria;n_Oscillatoriophycideae;o_Oscillatoriales; | 1382 | 77 | 113 | 91 | 424 | 282 | 395 |
| k_Bacteria;n_FCB group;p_Gemmatimonadetes;c_Gemmatimonadetes;o_Gemmatimonadales; | 1348 | 75 | 21 | 28 | 283 | 156 | 785 |
| k_Bacteria;n_FCB group;n_Bacteroidetes/Chlorobi group;p_Ignavibacteriae;c_Ignavibacteria;o_Ignavibacteriales; | 1340 | 65 | 43 | 78 | 241 | 135 | 778 |
| k_Bacteria;n_Terrabacteria group;p_Deinococcus-Thermus;c_Deinococci;o_Deinococcales; | 1339 | 162 | 114 | 64 | 333 | 315 | 351 |
| k_Bacteria;p_Acidobacteria;c_Acidobacteriia;o_Bryobacterales; | 1096 | 24 | 29 | 32 | 229 | 111 | 671 |
| k_Bacteria;n_Terrabacteria group;p_Firmicutes;c_Clostridia;o_Thermoanaerobacterales; | 1084 | 115 | 179 | 146 | 228 | 222 | 194 |
| k_Bacteria;n_Terrabacteria group;p_Actinobacteria;c_Actinobacteria;o_Geodermatophilales; | 960 | 46 | 34 | 53 | 258 | 202 | 367 |
| k_Bacteria;n_Terrabacteria group;p_Deinococcus-Thermus;c_Deinococci;o_Thermales; | 932 | 37 | 44 | 43 | 207 | 305 | 296 |
| k_Bacteria;n_PVC group;p_Verrucomicrobia;c_Opitutae;o_Puniceicoccales; | 917 | 209 | 195 | 336 | 86 | 56 | 35 |
| k_Bacteria;n_Terrabacteria group;p_Chloroflexi;c_Chloroflexia;o_Herpetosiphonales; | 879 | 7 | 18 | 16 | 379 | 234 | 225 |
| k_Bacteria;n_FCB group;n_Bacteroidetes/Chlorobi group;p_Chlorobi;c_Chlorobia;o_Chlorobiales; | 741 | 90 | 150 | 132 | 105 | 117 | 147 |
| k_Bacteria;n_Terrabacteria group;n_Cyanobacteria/Melainabacteria group;p_Cyanobacteria;n_Oscillatoriophycideae;o_Chroococcales; | 682 | 45 | 73 | 78 | 118 | 120 | 248 |
| k_Bacteria;p_Proteobacteria;c_Oligoflexia;o_Silvanigrellales; | 622 | 6 | 3 | 8 | 228 | 174 | 203 |
| k_Bacteria;n_Terrabacteria group;p_Actinobacteria;c_Actinobacteria;o_Nakamurellales; | 599 | 195 | 162 | 114 | 57 | 37 | 34 |
| k_Bacteria;p_Aquificae;c_Aquificae;o_Desulfurobacteriales; | 582 | 167 | 177 | 176 | 28 | 15 | 19 |
| k_Bacteria;n_Terrabacteria group;p_Chloroflexi;c_Ktedonobacteria;o_Ktedonobacterales; | 562 | 26 | 30 | 22 | 163 | 125 | 196 |
| k_Bacteria;n_Terrabacteria group;p_Actinobacteria;c_Actinobacteria;o_Candidatus Nanopelagicales; | 553 | 5 | 6 | 1 | 108 | 390 | 43 |
| k_Bacteria;p_Thermotogae;c_Thermotogae;o_Thermotogales; | 516 | 28 | 43 | 34 | 118 | 243 | 50 |
| k_Bacteria;n_Terrabacteria group;p_Firmicutes;c_Clostridia;o_Halanaerobiales; | 513 | 113 | 124 | 122 | 53 | 60 | 41 |
| k_Bacteria;n_PVC group;p_Planctomycetes;c_Candidatus Brocadiae;o_Candidatus Brocadiales; | 499 | 24 | 31 | 38 | 117 | 90 | 199 |
| k_Bacteria;n_Terrabacteria group;p_Chloroflexi;c_Dehalococcoidia;o_Dehalococcoidales; | 484 | 74 | 103 | 129 | 60 | 51 | 67 |
| k_Bacteria;p_Proteobacteria;c_Gammaproteobacteria;o_Orbales; | 439 | 92 | 138 | 117 | 43 | 29 | 20 |
| k_Bacteria;p_Proteobacteria;c_Gammaproteobacteria;o_Cardiobacteriales; | 429 | 83 | 131 | 99 | 38 | 20 | 58 |
| k_Bacteria;p_Proteobacteria;c_Betaproteobacteria;o_Ferrovales; | 419 | 67 | 134 | 82 | 29 | 44 | 63 |
| k_Bacteria;n_Terrabacteria group;p_Chloroflexi;c_Thermoflexia;o_Thermoflexales; | 393 | 19 | 9 | 14 | 60 | 41 | 250 |
| k_Bacteria;p_Spirochaetes;c_Spirochaetia;o_Brachyspirales; | 393 | 115 | 121 | 102 | 18 | 19 | 18 |
| k_Bacteria;p_Aquificae;c_Aquificae;o_Aquificales; | 372 | 57 | 69 | 70 | 72 | 46 | 58 |
| k_Bacteria;n_PVC group;p_Planctomycetes;c_Phycisphaerae;o_Phycisphaerales; | 368 | 14 | 8 | 14 | 105 | 55 | 172 |
| k_Bacteria;n_PVC group;p_Verrucomicrobia;c_Spartobacteria;o_Chthoniobacterales; | 350 | 20 | 35 | 26 | 88 | 72 | 109 |
| k_Bacteria;n_Terrabacteria group;p_Armatimonadetes;c_Armatimonadia;o_Capsulimonadales; | 325 | 12 | 9 | 4 | 125 | 79 | 96 |
| k_Bacteria;n_Terrabacteria group;p_Actinobacteria;c_Actinobacteria;o_Jiangellales; | 278 | 7 | 7 | 6 | 89 | 48 | 121 |
| k_Bacteria;n_Terrabacteria group;p_Actinobacteria;c_Actinobacteria;o_Kineosporiales; | 251 | 12 | 9 | 10 | 76 | 40 | 104 |
| k_Bacteria;n_Terrabacteria group;p_Tenericutes;c_Mollicutes;o_Acholeplasmatales; | 242 | 122 | 35 | 39 | 18 | 17 | 11 |
| k_Bacteria;p_Proteobacteria;c_Oligoflexia;o_Oligoflexales; | 235 | 1 | 8 | 4 | 75 | 36 | 111 |
| k_Bacteria;p_Proteobacteria;c_Alphaproteobacteria;o_Magnetococcales; | 197 | 18 | 29 | 24 | 40 | 27 | 59 |
| k_Bacteria;p_Proteobacteria;c_Gammaproteobacteria;o_Acidiferrobacterales; | 187 | 14 | 13 | 12 | 53 | 45 | 50 |
| k_Bacteria;p_Proteobacteria;c_Alphaproteobacteria;o_Sneathiellales; | 168 | 1 | 5 | 8 | 67 | 54 | 33 |
| k_Bacteria;n_Terrabacteria group;p_Chloroflexi;c_Thermomicrobia;n_Sphaerobacteridae;o_Sphaerobacterales; | 167 | 2 | 4 | 7 | 39 | 54 | 61 |
| k_Bacteria;p_Proteobacteria;n_delta/epsilon subdivisions;c_Deltaproteobacteria;o_Desulfarculales; | 166 | 17 | 39 | 37 | 25 | 15 | 33 |
| k_Bacteria;n_Caldiserica/Cryosericota group;p_Candidatus Cryosericota;c_Candidatus Cryosericia;o_Candidatus Cryosericales; | 158 | 13 | 18 | 34 | 20 | 17 | 56 |
| k_Bacteria;n_Terrabacteria group;p_Actinobacteria;c_Rubrobacteria;o_Gaiellales; | 153 | 3 | 12 | 5 | 34 | 47 | 52 |
| k_Bacteria;p_Proteobacteria;n_delta/epsilon subdivisions;c_Deltaproteobacteria;o_Desulfurellales; | 150 | 14 | 82 | 40 | 5 | 6 | 3 |
| k_Bacteria;n_Terrabacteria group;p_Armatimonadetes;c_Fimbriimonadia;o_Fimbriimonadales; | 145 | 10 | 3 | 2 | 59 | 17 | 54 |
| k_Bacteria;n_FCB group;n_Bacteroidetes/Chlorobi group;p_Bacteroidetes;o_Bacteroidetes Order II. Incertae sedis; | 143 | 5 | 13 | 8 | 38 | 27 | 52 |
| k_Bacteria;p_Proteobacteria;n_delta/epsilon subdivisions;c_Epsilonproteobacteria;o_Nautiliales; | 139 | 29 | 46 | 22 | 14 | 10 | 18 |
| k_Bacteria;n_unclassified Bacteria;n_Bacteria candidate phyla;n_Candidatus Dependentiae;c_Candidatus Babeliae;o_Candidatus Babeliales; | 137 | 5 | 3 | 2 | 82 | 31 | 14 |
| k_Bacteria;p_Thermotogae;c_Thermotogae;o_Petrotogales; | 132 | 18 | 26 | 25 | 21 | 20 | 22 |
| k_Bacteria;p_Proteobacteria;c_Gammaproteobacteria;o_Salinisphaerales; | 127 | 5 | 17 | 8 | 35 | 20 | 42 |
| k_Bacteria;p_Proteobacteria;c_Alphaproteobacteria;o_Micropepsales; | 118 | 7 | 2 | 2 | 51 | 12 | 44 |
| k_Bacteria;n_Terrabacteria group;n_Cyanobacteria/Melainabacteria group;p_Candidatus Margulisbacteria;c_Candidatus Termititenacia;o_Candidatus Termititenacales; | 117 | 8 | 19 | 7 | 36 | 35 | 12 |
| k_Bacteria;n_FCB group;n_Bacteroidetes/Chlorobi group;p_Balneolaeota;c_Balneolia;o_Balneolales; | 113 | 5 | 9 | 6 | 21 | 23 | 49 |
| k_Bacteria;n_FCB group;n_Bacteroidetes/Chlorobi group;p_Rhodothermaeota;c_Rhodothermia;o_Rhodothermales; | 104 | 7 | 6 | 2 | 49 | 12 | 28 |
| k_Bacteria;p_Proteobacteria;n_delta/epsilon subdivisions;c_Deltaproteobacteria;o_Bradymonadales; | 95 | 18 | 2 | 4 | 21 | 19 | 31 |
| k_Bacteria;n_Terrabacteria group;p_Tenericutes;c_Mollicutes;o_Entomoplasmatales; | 90 | 2 | 7 | 2 | 26 | 41 | 12 |
| k_Bacteria;p_Calditrichaeota;c_Calditrichae;o_Calditrichales; | 87 | 10 | 6 | 5 | 19 | 16 | 31 |
| k_Bacteria;p_Elusimicrobia;c_Endomicrobia;o_Endomicrobiales; | 86 | 7 | 37 | 27 | 4 | 6 | 5 |
| k_Bacteria;p_Acidobacteria;c_Blastocatellia;o_Blastocatellales; | 83 | 1 | 2 | 0 | 14 | 9 | 57 |
| k_Bacteria;n_Terrabacteria group;p_Actinobacteria;c_Actinobacteria;o_Glycomycetales; | 81 | 1 | 2 | 4 | 26 | 17 | 31 |
| k_Bacteria;p_Thermotogae;c_Thermotogae;o_Kosmotogales; | 79 | 19 | 16 | 12 | 10 | 10 | 12 |
| k_Bacteria;p_Proteobacteria;c_Alphaproteobacteria;o_Kordiimonadales; | 77 | 2 | 3 | 4 | 26 | 28 | 14 |
| k_Bacteria;n_Terrabacteria group;n_Cyanobacteria/Melainabacteria group;p_Cyanobacteria;o_Pleurocapsales; | 76 | 6 | 0 | 4 | 21 | 23 | 22 |
| k_Bacteria;n_Terrabacteria group;p_Actinobacteria;c_Nitriliruptoria;o_Euzebyales; | 72 | 4 | 1 | 4 | 21 | 17 | 25 |
| k_Bacteria;n_Terrabacteria group;p_Actinobacteria;c_Actinobacteria;o_Actinopolysporales; | 64 | 0 | 2 | 1 | 23 | 25 | 13 |
| k_Bacteria;p_Thermodesulfobacteria;c_Thermodesulfobacteria;o_Thermodesulfobacteriales; | 62 | 6 | 12 | 8 | 7 | 10 | 19 |
| k_Bacteria;n_Terrabacteria group;p_Actinobacteria;c_Nitriliruptoria;o_Egibacterales; | 60 | 1 | 0 | 5 | 10 | 9 | 35 |
| k_Bacteria;n_unclassified Bacteria;n_Bacteria candidate phyla;p_Candidatus Peregrinibacteria;c_Candidatus Peribacteria;o_Candidatus Peribacterales; | 60 | 2 | 3 | 1 | 10 | 22 | 22 |
| k_Bacteria;p_Proteobacteria;c_Alphaproteobacteria;o_Kiloniellales; | 53 | 3 | 8 | 3 | 14 | 13 | 12 |
| k_Bacteria;n_Terrabacteria group;p_Actinobacteria;c_Rubrobacteria;o_Rubrobacterales; | 52 | 2 | 3 | 3 | 19 | 10 | 15 |
| k_Bacteria;p_Proteobacteria;c_Gammaproteobacteria;o_Immundisolibacterales; | 52 | 7 | 1 | 5 | 8 | 6 | 25 |
| k_Bacteria;n_Terrabacteria group;p_Actinobacteria;c_Actinobacteria;o_Catenulisporales; | 50 | 2 | 3 | 2 | 27 | 10 | 6 |
| k_Bacteria;n_Terrabacteria group;p_Armatimonadetes;c_Chthonomonadetes;o_Chthonomonadales; | 49 | 4 | 3 | 2 | 13 | 3 | 24 |
| k_Bacteria;n_Terrabacteria group;n_Cyanobacteria/Melainabacteria group;p_Cyanobacteria;c_Gloeobacteria;o_Gloeobacterales; | 47 | 4 | 4 | 6 | 12 | 7 | 14 |
| k_Bacteria;p_Chrysiogenetes;c_Chrysiogenetes;o_Chrysiogenales; | 45 | 7 | 19 | 9 | 3 | 2 | 5 |
| k_Bacteria;p_Proteobacteria;c_Alphaproteobacteria;o_Emcibacterales; | 45 | 5 | 9 | 0 | 15 | 8 | 8 |
| k_Bacteria;n_Terrabacteria group;p_Tenericutes;c_Mollicutes;o_Anaeroplasmatales; | 44 | 4 | 2 | 3 | 1 | 34 | 0 |
| k_Bacteria;n_Terrabacteria group;p_Actinobacteria;c_Thermoleophilia;o_Thermoleophilales; | 41 | 2 | 0 | 3 | 14 | 3 | 19 |
| k_Bacteria;n_FCB group;p_Fibrobacteres;c_Chitinispirillia;o_Chitinispirillales; | 40 | 3 | 4 | 0 | 16 | 6 | 11 |
| k_Bacteria;n_PVC group;p_Kiritimatiellaeota;c_Kiritimatiellae;o_Kiritimatiellales; | 40 | 3 | 6 | 3 | 9 | 8 | 11 |
| k_Bacteria;n_Nitrospinae/Tectomicrobia group;p_Nitrospinae;c_Nitrospinia;o_Nitrospinales; | 39 | 2 | 4 | 2 | 13 | 9 | 9 |
| k_Bacteria;n_Terrabacteria group;p_Actinobacteria;c_Nitriliruptoria;o_Nitriliruptorales; | 38 | 1 | 0 | 6 | 17 | 3 | 11 |
| k_Bacteria;p_Proteobacteria;c_Alphaproteobacteria;o_Parvularculales; | 35 | 3 | 2 | 1 | 8 | 8 | 13 |
| k_Bacteria;p_Proteobacteria;c_Zetaproteobacteria;o_Mariprofundales; | 33 | 2 | 1 | 2 | 13 | 4 | 11 |
| k_Bacteria;n_Terrabacteria group;n_Cyanobacteria/Melainabacteria group;p_Cyanobacteria;o_Chroococcidiopsidales; | 31 | 1 | 3 | 0 | 14 | 7 | 6 |
| k_Bacteria;n_Terrabacteria group;p_Abditibacteriota;c_Abditibacteria;o_Abditibacteriales; | 30 | 2 | 1 | 5 | 8 | 5 | 9 |
| k_Bacteria;n_unclassified Bacteria;n_Bacteria candidate phyla;p_Candidatus Sumerlaeota;c_Candidatus Sumerlaeia;o_Candidatus Sumerlaeales; | 30 | 1 | 2 | 2 | 10 | 3 | 12 |
| k_Bacteria;n_FCB group;p_Fibrobacteres;c_Chitinivibrionia;o_Chitinivibrionales; | 27 | 6 | 11 | 5 | 2 | 1 | 2 |
| k_Bacteria;n_PVC group;p_Planctomycetes;c_Phycisphaerae;o_Sedimentisphaerales; | 26 | 1 | 3 | 6 | 3 | 4 | 9 |
| k_Bacteria;n_PVC group;p_Lentisphaerae;c_Lentisphaeria;o_Lentisphaerales; | 23 | 0 | 1 | 3 | 5 | 7 | 7 |
| k_Bacteria;n_PVC group;p_Verrucomicrobia;c_Methylacidiphilae;o_Methylacidiphilales; | 22 | 3 | 3 | 2 | 3 | 2 | 9 |
| k_Bacteria;p_Dictyoglomi;c_Dictyoglomia;o_Dictyoglomales; | 22 | 3 | 0 | 3 | 6 | 3 | 7 |
| k_Bacteria;n_Terrabacteria group;p_Chloroflexi;c_Ktedonobacteria;o_Thermogemmatisporales; | 20 | 0 | 0 | 1 | 5 | 4 | 10 |
| k_Bacteria;n_Caldiserica/Cryosericota group;p_Caldiserica;c_Caldisericia;o_Caldisericales; | 18 | 1 | 2 | 3 | 2 | 5 | 5 |
| k_Bacteria;n_FCB group;p_Candidatus Fermentibacteria;c_Candidatus Fermentibacteria (class);o_Candidatus Fermentibacterales; | 17 | 3 | 4 | 1 | 6 | 2 | 1 |
| k_Bacteria;n_Terrabacteria group;n_Cyanobacteria/Melainabacteria group;p_Cyanobacteria;o_Spirulinales; | 17 | 0 | 3 | 2 | 0 | 3 | 9 |
| k_Bacteria;p_Coprothermobacterota;c_Coprothermobacteria;o_Coprothermobacterales; | 15 | 2 | 7 | 1 | 2 | 0 | 3 |
| k_Bacteria;n_Terrabacteria group;p_Actinobacteria;c_Nitriliruptoria;o_Egicoccales; | 14 | 2 | 0 | 1 | 0 | 3 | 8 |
| k_Bacteria;p_Proteobacteria;c_Alphaproteobacteria;o_Rhodothalassiales; | 14 | 0 | 2 | 2 | 5 | 1 | 4 |
| k_Bacteria;n_Terrabacteria group;p_Firmicutes;c_Limnochordia;o_Limnochordales; | 13 | 6 | 0 | 1 | 0 | 0 | 6 |
| k_Bacteria;n_unclassified Bacteria;o_Haloplasmatales; | 13 | 0 | 6 | 5 | 1 | 1 | 0 |
| k_Bacteria;n_Terrabacteria group;p_Firmicutes;c_Clostridia;o_Candidatus Comantemales; | 11 | 0 | 5 | 3 | 0 | 3 | 0 |
| k_Bacteria;p_Proteobacteria;c_Alphaproteobacteria;o_Minwuiales; | 11 | 0 | 1 | 1 | 4 | 2 | 3 |
| k_Bacteria;p_Proteobacteria;c_Gammaproteobacteria;o_Arenicellales; | 11 | 3 | 3 | 2 | 1 | 0 | 2 |
| k_Bacteria;p_Thermotogae;c_Thermotogae;o_Mesoaciditogales; | 11 | 3 | 0 | 5 | 2 | 0 | 1 |
| k_Bacteria;n_Terrabacteria group;p_Firmicutes;c_Clostridia;o_Natranaerobiales; | 10 | 5 | 1 | 2 | 0 | 0 | 2 |
| k_Bacteria;p_Acidobacteria;c_Thermoanaerobaculia;o_Thermoanaerobaculales; | 10 | 0 | 1 | 0 | 0 | 0 | 9 |
| k_Bacteria;n_Terrabacteria group;p_Chloroflexi;c_Thermomicrobia;o_Thermomicrobiales; | 9 | 2 | 0 | 0 | 1 | 1 | 5 |
| k_Bacteria;n_Terrabacteria group;n_Cyanobacteria/Melainabacteria group;p_Cyanobacteria;o_Gloeoemargaritales; | 6 | 0 | 0 | 0 | 1 | 3 | 2 |
| k_Bacteria;n_Terrabacteria group;p_Actinobacteria;c_Actinobacteria;n_Candidatus Actinomarinidae;o_Candidatus Actinomarinales; | 3 | 0 | 3 | 0 | 0 | 0 | 0 |
| k_Bacteria;n_Terrabacteria group;p_Actinobacteria;c_Actinobacteria;o_Acidothermales; | 3 | 2 | 0 | 0 | 0 | 0 | 1 |
| k_Bacteria;p_Spirochaetes;c_Spirochaetia;o_Brevinematales; | 2 | 1 | 0 | 0 | 0 | 1 | 0 |
| **Total** | **15269311** | **4236853** | **5022672** | **3935840** | **605186** | **535254** | **933506** |

**Table S7 Number of reads for each bacterial Family**

| **Family** | **Total** | **RAW1** | **RAW2** | **RAW3** | **UV1** | **UV2** | **UV3** |
| --- | --- | --- | --- | --- | --- | --- | --- |
| "k_Bacteria;p_Proteobacteria;n_delta/epsilon subdivisions;c_Epsilonproteobacteria;o_Campylobacterales;f_Campylobacteraceae;" | 3213062 | 926267 | 1357439 | 896752 | 14456 | 12814 | 5334 |
| "k_Bacteria;p_Proteobacteria;c_Betaproteobacteria;o_Burkholderiales;f_Comamonadaceae;" | 1598179 | 458976 | 501146 | 406894 | 59104 | 67386 | 104673 |
| "k_Bacteria;p_Proteobacteria;c_Gammaproteobacteria;o_Aeromonadales;f_Aeromonadaceae;" | 1379444 | 440137 | 454853 | 461229 | 12063 | 7050 | 4112 |
| "k_Bacteria;n_FCB group;n_Bacteroidetes/Chlorobi group;p_Bacteroidetes;c_Bacteroidia;o_Bacteroidales;f_Bacteroidaceae;" | 1211544 | 402835 | 471106 | 305824 | 16246 | 12114 | 3419 |
| "k_Bacteria;p_Proteobacteria;c_Gammaproteobacteria;o_Pseudomonadales;f_Moraxellaceae;" | 873447 | 343640 | 298205 | 213516 | 7248 | 5782 | 5056 |
| "k_Bacteria;n_FCB group;n_Bacteroidetes/Chlorobi group;p_Bacteroidetes;c_Bacteroidia;o_Bacteroidales;f_Prevotellaceae;" | 482839 | 209250 | 130282 | 130061 | 6099 | 5691 | 1456 |
| "k_Bacteria;p_Proteobacteria;c_Gammaproteobacteria;o_Pseudomonadales;f_Pseudomonadaceae;" | 423323 | 120141 | 141989 | 136848 | 8665 | 7154 | 8526 |
| "k_Bacteria;p_Proteobacteria;c_Gammaproteobacteria;o_Enterobacterales;f_Enterobacteriaceae;" | 360405 | 121656 | 119363 | 98404 | 10802 | 5609 | 4571 |
| "k_Bacteria;n_FCB group;n_Bacteroidetes/Chlorobi group;p_Bacteroidetes;c_Flavobacteriia;o_Flavobacteriales;f_Flavobacteriaceae;" | 342521 | 98559 | 113227 | 83051 | 10517 | 30466 | 6701 |
| "k_Bacteria;n_FCB group;n_Bacteroidetes/Chlorobi group;p_Bacteroidetes;c_Bacteroidia;o_Bacteroidales;f_Rikenellaceae;" | 204192 | 52525 | 71877 | 73334 | 3576 | 1951 | 929 |
| "k_Bacteria;n_Terrabacteria group;p_Actinobacteria;c_Actinobacteria;o_Corynebacteriales;f_Gordoniaceae;" | 198193 | 7905 | 2133 | 13778 | 26941 | 9025 | 138411 |
| "k_Bacteria;p_Proteobacteria;c_Betaproteobacteria;o_Neisseriales;f_Neisseriaceae;" | 188970 | 63079 | 54751 | 45803 | 4065 | 12961 | 8311 |
| "k_Bacteria;n_Terrabacteria group;p_Firmicutes;c_Clostridia;o_Clostridiales;f_Ruminococcaceae;" | 179558 | 59051 | 56046 | 59655 | 2718 | 1210 | 878 |
| "k_Bacteria;p_Proteobacteria;c_Betaproteobacteria;o_Neisseriales;f_Chromobacteriaceae;" | 160145 | 36851 | 65218 | 48837 | 2056 | 4824 | 2359 |
| "k_Bacteria;n_FCB group;n_Bacteroidetes/Chlorobi group;p_Bacteroidetes;c_Bacteroidia;o_Bacteroidales;f_Tannerellaceae;" | 120595 | 34390 | 53166 | 29731 | 1689 | 1272 | 347 |
| "k_Bacteria;n_Terrabacteria group;p_Firmicutes;c_Clostridia;o_Clostridiales;f_Lachnospiraceae;" | 112921 | 39180 | 33266 | 37885 | 1350 | 866 | 374 |
| "k_Bacteria;n_Terrabacteria group;p_Chloroflexi;c_Ardenticatenia;o_Ardenticatenales;f_Ardenticatenaceae;" | 96315 | 879 | 262 | 7195 | 2805 | 2965 | 82209 |
| "k_Bacteria;p_Proteobacteria;c_Gammaproteobacteria;o_Xanthomonadales;f_Xanthomonadaceae;" | 83868 | 21516 | 21224 | 19894 | 6423 | 6122 | 8689 |
| "k_Bacteria;n_Terrabacteria group;p_Firmicutes;c_Clostridia;o_Clostridiales;f_Clostridiaceae;" | 75649 | 23638 | 25198 | 23143 | 1634 | 1298 | 738 |
| "k_Bacteria;p_Proteobacteria;n_delta/epsilon subdivisions;c_Deltaproteobacteria;o_Desulfovibrionales;f_Desulfovibrionaceae;" | 75318 | 13942 | 38344 | 19120 | 1381 | 1409 | 1122 |
| "k_Bacteria;n_Terrabacteria group;p_Actinobacteria;c_Actinobacteria;o_Corynebacteriales;f_Mycobacteriaceae;" | 61031 | 2818 | 3358 | 2142 | 23165 | 14290 | 15258 |
| "k_Bacteria;p_Proteobacteria;c_Betaproteobacteria;o_Rhodocyclales;f_Zoogloeaceae;" | 60674 | 15496 | 16141 | 20179 | 1541 | 1599 | 5718 |
| "k_Bacteria;n_Terrabacteria group;p_Firmicutes;c_Bacilli;o_Lactobacillales;f_Streptococcaceae;" | 57401 | 13406 | 21346 | 21520 | 414 | 413 | 302 |
| "k_Bacteria;n_Terrabacteria group;p_Firmicutes;c_Clostridia;o_Clostridiales;f_Eubacteriaceae;" | 55438 | 17956 | 17720 | 18626 | 608 | 339 | 189 |
| "k_Bacteria;n_Terrabacteria group;p_Firmicutes;c_Clostridia;o_Clostridiales;f_Peptostreptococcaceae;" | 54425 | 9453 | 22304 | 21133 | 527 | 752 | 256 |
| "k_Bacteria;p_Proteobacteria;c_Betaproteobacteria;o_Rhodocyclales;f_Rhodocyclaceae;" | 52261 | 7964 | 14609 | 9920 | 4437 | 9112 | 6219 |
| "k_Bacteria;p_Proteobacteria;c_Alphaproteobacteria;o_Rhodospirillales;f_Rhodospirillaceae;" | 51510 | 9189 | 14514 | 13021 | 5751 | 4211 | 4824 |
| "k_Bacteria;p_Proteobacteria;c_Betaproteobacteria;o_Burkholderiales;f_Burkholderiaceae;" | 51456 | 6527 | 10166 | 7895 | 7432 | 7758 | 11678 |
| "k_Bacteria;p_Proteobacteria;c_Alphaproteobacteria;o_Rhodobacterales;f_Rhodobacteraceae;" | 45684 | 10695 | 9623 | 11048 | 4410 | 4943 | 4965 |
| "k_Bacteria;n_FCB group;n_Bacteroidetes/Chlorobi group;p_Bacteroidetes;c_Sphingobacteriia;o_Sphingobacteriales;f_Sphingobacteriaceae;" | 44910 | 1295 | 1462 | 790 | 27735 | 10034 | 3594 |
| "k_Bacteria;p_Proteobacteria;c_Alphaproteobacteria;o_Sphingomonadales;f_Sphingomonadaceae;" | 40995 | 4212 | 4593 | 3843 | 5597 | 7956 | 14794 |
| "k_Bacteria;n_Terrabacteria group;p_Actinobacteria;c_Actinobacteria;o_Bifidobacteriales;f_Bifidobacteriaceae;" | 39252 | 12506 | 12296 | 12555 | 738 | 553 | 604 |
| "k_Bacteria;n_Terrabacteria group;p_Firmicutes;c_Negativicutes;o_Selenomonadales;f_Selenomonadaceae;" | 36036 | 11376 | 11745 | 11514 | 643 | 566 | 192 |
| "k_Bacteria;p_Proteobacteria;c_Gammaproteobacteria;o_Alteromonadales;f_Shewanellaceae;" | 34673 | 10373 | 11598 | 11698 | 664 | 169 | 171 |
| "k_Bacteria;n_FCB group;n_Bacteroidetes/Chlorobi group;p_Bacteroidetes;c_Chitinophagia;o_Chitinophagales;f_Chitinophagaceae;" | 34237 | 1837 | 1818 | 1540 | 4142 | 17618 | 7282 |
| "k_Bacteria;p_Proteobacteria;c_Alphaproteobacteria;o_Rhizobiales;f_Bradyrhizobiaceae;" | 32313 | 1743 | 1305 | 1670 | 6939 | 5868 | 14788 |
| "k_Bacteria;p_Proteobacteria;c_Betaproteobacteria;o_Burkholderiales;f_Sutterellaceae;" | 28238 | 9165 | 8252 | 10141 | 447 | 141 | 92 |
| "k_Bacteria;p_Fusobacteria;c_Fusobacteriia;o_Fusobacteriales;f_Leptotrichiaceae;" | 26352 | 4378 | 14413 | 7100 | 137 | 235 | 89 |
| "k_Bacteria;p_Proteobacteria;c_Alphaproteobacteria;o_Caulobacterales;f_Caulobacteraceae;" | 23928 | 5307 | 5471 | 4507 | 2237 | 1543 | 4863 |
| "k_Bacteria;n_Terrabacteria group;p_Firmicutes;c_Negativicutes;o_Veillonellales;f_Veillonellaceae;" | 22925 | 6429 | 8041 | 7618 | 373 | 296 | 168 |
| "k_Bacteria;n_Terrabacteria group;p_Firmicutes;c_Negativicutes;o_Acidaminococcales;f_Acidaminococcaceae;" | 20687 | 6220 | 6535 | 6863 | 528 | 378 | 163 |
| "k_Bacteria;p_Proteobacteria;c_Betaproteobacteria;o_Burkholderiales;f_Oxalobacteraceae;" | 20564 | 2093 | 3596 | 2408 | 3254 | 5768 | 3445 |
| "k_Bacteria;n_FCB group;n_Bacteroidetes/Chlorobi group;p_Bacteroidetes;c_Bacteroidia;o_Bacteroidales;f_Odoribacteraceae;" | 19543 | 6082 | 4880 | 7488 | 634 | 352 | 107 |
| "k_Bacteria;p_Proteobacteria;c_Gammaproteobacteria;o_Vibrionales;f_Vibrionaceae;" | 19430 | 5626 | 7229 | 5287 | 502 | 368 | 418 |
| "k_Bacteria;n_Terrabacteria group;p_Actinobacteria;c_Coriobacteriia;o_Coriobacteriales;f_Coriobacteriaceae;" | 18923 | 6001 | 5763 | 6269 | 467 | 274 | 149 |
| "k_Bacteria;p_Proteobacteria;c_Betaproteobacteria;o_Burkholderiales;f_Alcaligenaceae;" | 18533 | 3238 | 4111 | 3586 | 2176 | 2201 | 3221 |
| "k_Bacteria;p_Proteobacteria;c_Betaproteobacteria;o_Rhodocyclales;f_Azonexaceae;" | 18204 | 5973 | 4860 | 4426 | 784 | 381 | 1780 |
| "k_Bacteria;p_Proteobacteria;n_delta/epsilon subdivisions;c_Epsilonproteobacteria;o_Campylobacterales;f_Helicobacteraceae;" | 17450 | 4196 | 7242 | 5054 | 355 | 320 | 283 |
| "k_Bacteria;p_Nitrospirae;c_Nitrospira;o_Nitrospirales;f_Nitrospiraceae;" | 17258 | 532 | 143 | 244 | 4847 | 2870 | 8622 |
| "k_Bacteria;n_FCB group;n_Bacteroidetes/Chlorobi group;p_Bacteroidetes;c_Bacteroidia;o_Bacteroidales;f_Dysgonamonadaceae;" | 16860 | 3941 | 8790 | 3721 | 189 | 150 | 69 |
| "k_Bacteria;p_Proteobacteria;c_Alphaproteobacteria;o_Rhodospirillales;f_Acetobacteraceae;" | 16019 | 3792 | 3952 | 4133 | 1318 | 957 | 1867 |
| "k_Bacteria;n_Terrabacteria group;p_Firmicutes;c_Bacilli;o_Lactobacillales;f_Lactobacillaceae;" | 15885 | 4581 | 5960 | 4532 | 289 | 253 | 270 |
| "k_Bacteria;n_Terrabacteria group;p_Firmicutes;c_Negativicutes;o_Selenomonadales;f_Sporomusaceae;" | 15022 | 3201 | 6775 | 4488 | 196 | 252 | 110 |
| "k_Bacteria;p_Proteobacteria;n_delta/epsilon subdivisions;c_Deltaproteobacteria;o_Desulfobacterales;f_Desulfobulbaceae;" | 14343 | 2214 | 7593 | 3725 | 274 | 252 | 285 |
| "k_Bacteria;n_Terrabacteria group;p_Actinobacteria;c_Actinobacteria;o_Micrococcales;f_Intrasporangiaceae;" | 14085 | 1043 | 340 | 1351 | 2258 | 694 | 8399 |
| "k_Bacteria;p_Proteobacteria;n_delta/epsilon subdivisions;c_Deltaproteobacteria;o_Myxococcales;n_Cystobacterineae;f_Myxococcaceae;" | 13977 | 140 | 102 | 128 | 3159 | 1750 | 8698 |
| "k_Bacteria;p_Proteobacteria;c_Gammaproteobacteria;n_unclassified Gammaproteobacteria;f_Candidatus Competibacteraceae;" | 13953 | 1247 | 933 | 1217 | 2530 | 1421 | 6605 |
| "k_Bacteria;p_Proteobacteria;c_Gammaproteobacteria;o_Aeromonadales;f_Succinivibrionaceae;" | 13726 | 5157 | 3684 | 4510 | 237 | 105 | 33 |
| "k_Bacteria;n_Terrabacteria group;p_Actinobacteria;c_Actinobacteria;o_Propionibacteriales;f_Nocardioidaceae;" | 13673 | 1668 | 1009 | 1021 | 3482 | 1673 | 4820 |
| "k_Bacteria;p_Proteobacteria;c_Oligoflexia;o_Bdellovibrionales;f_Bdellovibrionaceae;" | 13541 | 291 | 258 | 254 | 6316 | 2869 | 3553 |
| "k_Bacteria;n_FCB group;n_Bacteroidetes/Chlorobi group;p_Bacteroidetes;c_Bacteroidia;o_Bacteroidales;f_Porphyromonadaceae;" | 13229 | 2637 | 7186 | 2957 | 176 | 152 | 121 |
| "k_Bacteria;p_Fusobacteria;c_Fusobacteriia;o_Fusobacteriales;f_Fusobacteriaceae;" | 13075 | 3562 | 4952 | 4212 | 161 | 120 | 68 |
| "k_Bacteria;p_Proteobacteria;c_Alphaproteobacteria;o_Rhizobiales;f_Phyllobacteriaceae;" | 12449 | 766 | 782 | 852 | 3313 | 2652 | 4084 |
| "k_Bacteria;n_Terrabacteria group;p_Firmicutes;c_Clostridia;o_Clostridiales;f_Oscillospiraceae;" | 12138 | 3421 | 3829 | 4609 | 139 | 83 | 57 |
| "k_Bacteria;p_Proteobacteria;c_Betaproteobacteria;o_Nitrosomonadales;f_Nitrosomonadaceae;" | 12041 | 1103 | 957 | 912 | 3154 | 2175 | 3740 |
| "k_Bacteria;n_Terrabacteria group;p_Actinobacteria;c_Actinobacteria;o_Streptomycetales;f_Streptomycetaceae;" | 11892 | 441 | 518 | 552 | 3456 | 2932 | 3993 |
| "k_Bacteria;p_Proteobacteria;c_Alphaproteobacteria;o_Rhizobiales;f_Rhizobiaceae;" | 11638 | 1925 | 1966 | 2071 | 2233 | 1686 | 1757 |
| "k_Bacteria;n_PVC group;p_Verrucomicrobia;c_Verrucomicrobiae;o_Verrucomicrobiales;f_Akkermansiaceae;" | 11406 | 3041 | 3755 | 3862 | 359 | 278 | 111 |
| "k_Bacteria;p_Proteobacteria;c_Gammaproteobacteria;o_Enterobacterales;f_Yersiniaceae;" | 11404 | 4701 | 3592 | 2330 | 371 | 234 | 176 |
| "k_Bacteria;n_Terrabacteria group;p_Firmicutes;c_Erysipelotrichia;o_Erysipelotrichales;f_Erysipelotrichaceae;" | 11070 | 3880 | 3345 | 3397 | 138 | 250 | 60 |
| "k_Bacteria;n_Terrabacteria group;p_Actinobacteria;c_Actinobacteria;o_Micrococcales;f_Microbacteriaceae;" | 10953 | 2260 | 2197 | 2593 | 1258 | 1090 | 1555 |
| "k_Bacteria;p_Proteobacteria;c_Gammaproteobacteria;o_Chromatiales;f_Chromatiaceae;" | 10795 | 3690 | 1207 | 4505 | 339 | 230 | 824 |
| "k_Bacteria;p_Proteobacteria;c_Betaproteobacteria;o_Nitrosomonadales;f_Sterolibacteriaceae;" | 10222 | 738 | 1513 | 1019 | 1516 | 1687 | 3749 |
| "k_Bacteria;p_Proteobacteria;n_delta/epsilon subdivisions;c_Deltaproteobacteria;o_Desulfovibrionales;f_Desulfomicrobiaceae;" | 10159 | 1128 | 6183 | 2617 | 61 | 105 | 65 |
| "k_Bacteria;p_Synergistetes;c_Synergistia;o_Synergistales;f_Synergistaceae;" | 9607 | 1519 | 5523 | 1944 | 237 | 277 | 107 |
| "k_Bacteria;n_Terrabacteria group;p_Firmicutes;c_Bacilli;o_Lactobacillales;f_Enterococcaceae;" | 9560 | 2414 | 2986 | 2332 | 426 | 802 | 600 |
| "k_Bacteria;n_Terrabacteria group;p_Firmicutes;c_Bacilli;o_Lactobacillales;f_Carnobacteriaceae;" | 9484 | 2702 | 2231 | 1201 | 914 | 1309 | 1127 |
| "k_Bacteria;p_Spirochaetes;c_Spirochaetia;o_Leptospirales;f_Leptospiraceae;" | 9364 | 460 | 351 | 390 | 2054 | 1066 | 5043 |
| "k_Bacteria;n_PVC group;p_Verrucomicrobia;c_Opitutae;o_Opitutales;f_Opitutaceae;" | 8501 | 499 | 1769 | 1349 | 1910 | 1059 | 1915 |
| "k_Bacteria;p_Proteobacteria;c_Gammaproteobacteria;o_Xanthomonadales;f_Rhodanobacteraceae;" | 8330 | 1625 | 1969 | 1520 | 830 | 604 | 1782 |
| "k_Bacteria;p_Proteobacteria;n_delta/epsilon subdivisions;c_Deltaproteobacteria;o_Myxococcales;n_Sorangiineae;f_Polyangiaceae;" | 8226 | 143 | 98 | 107 | 1562 | 977 | 5339 |
| "k_Bacteria;p_Proteobacteria;c_Alphaproteobacteria;o_Rhizobiales;f_Hyphomicrobiaceae;" | 8102 | 406 | 412 | 424 | 1731 | 1300 | 3829 |
| "k_Bacteria;p_Acidobacteria;c_Holophagae;o_Holophagales;f_Holophagaceae;" | 7777 | 1026 | 3481 | 1784 | 362 | 257 | 867 |
| "k_Bacteria;n_Terrabacteria group;p_Firmicutes;c_Bacilli;o_Bacillales;f_Bacillaceae;" | 7732 | 1163 | 1937 | 1539 | 1132 | 1103 | 858 |
| "k_Bacteria;p_Proteobacteria;c_Gammaproteobacteria;o_Pasteurellales;f_Pasteurellaceae;" | 6063 | 1531 | 1997 | 1652 | 330 | 306 | 247 |
| "k_Bacteria;p_Proteobacteria;n_delta/epsilon subdivisions;c_Deltaproteobacteria;o_Desulfobacterales;f_Desulfobacteraceae;" | 5990 | 951 | 2815 | 1044 | 399 | 422 | 359 |
| "k_Bacteria;n_Terrabacteria group;p_Actinobacteria;c_Coriobacteriia;o_Eggerthellales;f_Eggerthellaceae;" | 5729 | 1809 | 1750 | 1794 | 196 | 148 | 32 |
| "k_Bacteria;p_Proteobacteria;c_Gammaproteobacteria;o_Enterobacterales;f_Morganellaceae;" | 5523 | 1666 | 1839 | 1347 | 310 | 203 | 158 |
| "k_Bacteria;p_Proteobacteria;n_delta/epsilon subdivisions;c_Deltaproteobacteria;o_Desulfuromonadales;f_Desulfuromonadaceae;" | 5209 | 683 | 2683 | 1587 | 106 | 63 | 87 |
| "k_Bacteria;p_Proteobacteria;c_Gammaproteobacteria;o_Legionellales;f_Legionellaceae;" | 5175 | 541 | 477 | 397 | 1690 | 1240 | 830 |
| "k_Bacteria;p_Proteobacteria;n_delta/epsilon subdivisions;c_Deltaproteobacteria;o_Myxococcales;n_Cystobacterineae;f_Archangiaceae;" | 5111 | 39 | 44 | 26 | 1477 | 859 | 2666 |
| "k_Bacteria;n_Terrabacteria group;p_Actinobacteria;c_Actinobacteria;o_Propionibacteriales;f_Propionibacteriaceae;" | 5042 | 1257 | 1586 | 1353 | 244 | 211 | 391 |
| "k_Bacteria;p_Proteobacteria;c_Gammaproteobacteria;o_Methylococcales;f_Methylococcaceae;" | 4954 | 465 | 711 | 602 | 1048 | 802 | 1326 |
| "k_Bacteria;n_FCB group;n_Bacteroidetes/Chlorobi group;p_Bacteroidetes;c_Cytophagia;o_Cytophagales;f_Cytophagaceae;" | 4841 | 385 | 501 | 591 | 1019 | 863 | 1482 |
| "k_Bacteria;n_Terrabacteria group;p_Actinobacteria;c_Acidimicrobiia;o_Acidimicrobiales;f_Microthrixaceae;" | 4815 | 836 | 67 | 203 | 1139 | 218 | 2352 |
| "k_Bacteria;n_Terrabacteria group;p_Chloroflexi;c_Chloroflexia;o_Chloroflexales;n_Roseiflexineae;f_Roseiflexaceae;" | 4792 | 443 | 37 | 64 | 1298 | 613 | 2337 |
| "k_Bacteria;n_PVC group;p_Planctomycetes;c_Planctomycetia;o_Planctomycetales;f_Planctomycetaceae;" | 4763 | 206 | 174 | 234 | 1492 | 1024 | 1633 |
| "k_Bacteria;n_Terrabacteria group;p_Firmicutes;c_Bacilli;o_Bacillales;f_Paenibacillaceae;" | 4746 | 597 | 885 | 702 | 825 | 939 | 798 |
| "k_Bacteria;n_Terrabacteria group;p_Actinobacteria;c_Actinobacteria;o_Corynebacteriales;f_Nocardiaceae;" | 4693 | 292 | 325 | 278 | 1240 | 860 | 1698 |
| "k_Bacteria;n_Terrabacteria group;p_Chloroflexi;c_Anaerolineae;o_Anaerolineales;f_Anaerolineaceae;" | 4421 | 201 | 146 | 132 | 1011 | 693 | 2238 |
| "k_Bacteria;p_Proteobacteria;c_Gammaproteobacteria;o_Alteromonadales;f_Alteromonadaceae;" | 4314 | 660 | 864 | 674 | 376 | 1043 | 697 |
| "k_Bacteria;n_FCB group;n_Bacteroidetes/Chlorobi group;p_Bacteroidetes;c_Bacteroidia;o_Bacteroidales;f_Williamwhitmaniaceae;" | 4257 | 424 | 2563 | 1209 | 38 | 17 | 6 |
| "k_Bacteria;p_Proteobacteria;c_Gammaproteobacteria;o_Enterobacterales;f_Pectobacteriaceae;" | 4172 | 984 | 1885 | 1108 | 76 | 52 | 67 |
| "k_Bacteria;n_Terrabacteria group;p_Actinobacteria;c_Actinobacteria;o_Actinomycetales;f_Actinomycetaceae;" | 4160 | 985 | 1342 | 1114 | 237 | 164 | 318 |
| "k_Bacteria;p_Proteobacteria;c_Alphaproteobacteria;o_Rhizobiales;f_Methylobacteriaceae;" | 4095 | 482 | 696 | 450 | 966 | 610 | 891 |
| "k_Bacteria;p_Proteobacteria;n_delta/epsilon subdivisions;c_Deltaproteobacteria;o_Syntrophobacterales;f_Syntrophaceae;" | 4048 | 840 | 530 | 787 | 801 | 608 | 482 |
| "k_Bacteria;p_Proteobacteria;c_Alphaproteobacteria;o_Rhizobiales;f_Methylocystaceae;" | 4046 | 647 | 1760 | 967 | 292 | 135 | 245 |
| "k_Bacteria;n_PVC group;p_Chlamydiae;c_Chlamydiia;o_Chlamydiales;f_Chlamydiaceae;" | 3942 | 1018 | 1182 | 1167 | 156 | 148 | 271 |
| "k_Bacteria;p_Proteobacteria;n_delta/epsilon subdivisions;c_Deltaproteobacteria;o_Desulfuromonadales;f_Geobacteraceae;" | 3937 | 744 | 1657 | 947 | 210 | 149 | 230 |
| "k_Bacteria;p_Deferribacteres;c_Deferribacteres;o_Deferribacterales;f_Deferribacteraceae;" | 3676 | 347 | 2079 | 1171 | 27 | 35 | 17 |
| "k_Bacteria;n_PVC group;p_Verrucomicrobia;c_Verrucomicrobiae;o_Verrucomicrobiales;f_Verrucomicrobiaceae;" | 3529 | 214 | 286 | 233 | 1027 | 725 | 1044 |
| "k_Bacteria;n_Terrabacteria group;p_Firmicutes;c_Bacilli;o_Lactobacillales;f_Leuconostocaceae;" | 3424 | 1485 | 1449 | 423 | 32 | 24 | 11 |
| "k_Bacteria;p_Spirochaetes;c_Spirochaetia;o_Spirochaetales;f_Spirochaetaceae;" | 3326 | 440 | 832 | 694 | 586 | 322 | 452 |
| "k_Bacteria;n_Terrabacteria group;p_Firmicutes;c_Clostridia;o_Clostridiales;f_Peptococcaceae;" | 3289 | 451 | 909 | 752 | 426 | 438 | 313 |
| "k_Bacteria;n_PVC group;p_Planctomycetes;c_Planctomycetia;o_Planctomycetales;f_Gemmataceae;" | 3240 | 111 | 87 | 70 | 1273 | 706 | 993 |
| "k_Bacteria;p_Proteobacteria;n_delta/epsilon subdivisions;c_Deltaproteobacteria;o_Syntrophobacterales;f_Syntrophorhabdaceae;" | 3203 | 924 | 656 | 803 | 370 | 254 | 196 |
| "k_Bacteria;n_Terrabacteria group;p_Actinobacteria;c_Actinobacteria;o_Micromonosporales;f_Micromonosporaceae;" | 3161 | 79 | 95 | 158 | 894 | 756 | 1179 |
| "k_Bacteria;p_Proteobacteria;c_Gammaproteobacteria;o_Enterobacterales;f_Erwiniaceae;" | 2998 | 1011 | 963 | 736 | 133 | 95 | 60 |
| "k_Bacteria;p_Proteobacteria;c_Gammaproteobacteria;o_Oceanospirillales;f_Oceanospirillaceae;" | 2960 | 602 | 1055 | 774 | 139 | 170 | 220 |
| "k_Bacteria;p_Proteobacteria;c_Alphaproteobacteria;o_Rickettsiales;f_Rickettsiaceae;" | 2782 | 47 | 45 | 42 | 1041 | 960 | 647 |
| "k_Bacteria;n_FCB group;n_Bacteroidetes/Chlorobi group;p_Bacteroidetes;c_Bacteroidia;o_Bacteroidales;f_Paludibacteraceae;" | 2734 | 460 | 1476 | 665 | 62 | 43 | 28 |
| "k_Bacteria;p_Proteobacteria;n_delta/epsilon subdivisions;c_Deltaproteobacteria;o_Myxococcales;n_Nannocystineae;f_Kofleriaceae;" | 2711 | 79 | 9 | 13 | 830 | 185 | 1595 |
| "k_Bacteria;n_Terrabacteria group;p_Actinobacteria;c_Actinobacteria;o_Pseudonocardiales;f_Pseudonocardiaceae;" | 2601 | 75 | 83 | 90 | 768 | 714 | 871 |
| "k_Bacteria;n_Terrabacteria group;p_Actinobacteria;c_Actinobacteria;o_Micrococcales;f_Micrococcaceae;" | 2574 | 278 | 306 | 291 | 695 | 440 | 564 |
| "k_Bacteria;n_Terrabacteria group;n_Cyanobacteria/Melainabacteria group;p_Cyanobacteria;o_Nostocales;f_Aphanizomenonaceae;" | 2478 | 35 | 44 | 78 | 695 | 500 | 1126 |
| "k_Bacteria;p_Proteobacteria;c_Gammaproteobacteria;o_Oceanospirillales;f_Halomonadaceae;" | 2414 | 376 | 487 | 401 | 450 | 314 | 386 |
| "k_Bacteria;p_Elusimicrobia;c_Elusimicrobia;o_Elusimicrobiales;f_Elusimicrobiaceae;" | 2373 | 252 | 1398 | 613 | 44 | 53 | 13 |
| "k_Bacteria;p_Proteobacteria;c_Betaproteobacteria;o_Nitrosomonadales;f_Methylophilaceae;" | 2309 | 332 | 596 | 574 | 280 | 271 | 256 |
| "k_Bacteria;n_FCB group;p_Fibrobacteres;c_Fibrobacteria;o_Fibrobacterales;f_Fibrobacteraceae;" | 2220 | 561 | 777 | 561 | 102 | 172 | 47 |
| "k_Bacteria;n_FCB group;n_Bacteroidetes/Chlorobi group;p_Bacteroidetes;c_Bacteroidia;o_Bacteroidales;f_Muribaculaceae;" | 2113 | 792 | 577 | 641 | 52 | 26 | 25 |
| "k_Bacteria;n_Terrabacteria group;p_Actinobacteria;c_Actinobacteria;o_Micrococcales;f_Sanguibacteraceae;" | 2008 | 811 | 549 | 604 | 16 | 7 | 21 |
| "k_Bacteria;n_FCB group;n_Bacteroidetes/Chlorobi group;p_Bacteroidetes;c_Bacteroidia;o_Bacteroidales;f_Barnesiellaceae;" | 1990 | 488 | 652 | 781 | 32 | 33 | 4 |
| "k_Bacteria;p_Proteobacteria;n_delta/epsilon subdivisions;c_Deltaproteobacteria;o_Myxococcales;n_Sorangiineae;f_Sandaracinaceae;" | 1982 | 16 | 9 | 21 | 448 | 342 | 1146 |
| "k_Bacteria;p_Proteobacteria;n_delta/epsilon subdivisions;c_Deltaproteobacteria;o_Myxococcales;n_Sorangiineae;f_Labilitrichaceae;" | 1937 | 15 | 18 | 36 | 414 | 362 | 1092 |
| "k_Bacteria;n_FCB group;n_Bacteroidetes/Chlorobi group;p_Bacteroidetes;c_Bacteroidia;o_Marinilabiliales;f_Marinilabiliaceae;" | 1826 | 268 | 843 | 614 | 38 | 31 | 32 |
| "k_Bacteria;n_Terrabacteria group;p_Firmicutes;c_Bacilli;o_Bacillales;f_Staphylococcaceae;" | 1805 | 235 | 328 | 263 | 364 | 342 | 273 |
| "k_Bacteria;p_Proteobacteria;c_Alphaproteobacteria;o_Sphingomonadales;f_Erythrobacteraceae;" | 1641 | 280 | 295 | 253 | 202 | 221 | 390 |
| "k_Bacteria;n_PVC group;p_Chlamydiae;c_Chlamydiia;o_Parachlamydiales;f_Parachlamydiaceae;" | 1639 | 45 | 63 | 70 | 382 | 484 | 595 |
| "k_Bacteria;n_Terrabacteria group;p_Chloroflexi;c_Caldilineae;o_Caldilineales;f_Caldilineaceae;" | 1508 | 136 | 22 | 83 | 199 | 72 | 996 |
| "k_Bacteria;p_Proteobacteria;c_Gammaproteobacteria;o_Nevskiales;f_Sinobacteraceae;" | 1501 | 76 | 66 | 94 | 150 | 113 | 1002 |
| "k_Bacteria;p_Proteobacteria;c_Acidithiobacillia;o_Acidithiobacillales;f_Acidithiobacillaceae;" | 1455 | 212 | 536 | 445 | 52 | 85 | 125 |
| "k_Bacteria;n_FCB group;n_Bacteroidetes/Chlorobi group;p_Bacteroidetes;c_Cytophagia;o_Cytophagales;f_Hymenobacteraceae;" | 1454 | 224 | 100 | 75 | 299 | 286 | 470 |
| "k_Bacteria;n_PVC group;p_Lentisphaerae;c_Lentisphaeria;o_Victivallales;f_Victivallaceae;" | 1450 | 224 | 441 | 625 | 78 | 61 | 21 |
| "k_Bacteria;p_Proteobacteria;c_Alphaproteobacteria;o_Rhizobiales;f_Xanthobacteraceae;" | 1429 | 184 | 210 | 193 | 270 | 219 | 353 |
| "k_Bacteria;p_Proteobacteria;n_delta/epsilon subdivisions;c_Deltaproteobacteria;o_Myxococcales;n_Nannocystineae;f_Nannocystaceae;" | 1427 | 72 | 8 | 4 | 613 | 375 | 355 |
| "k_Bacteria;n_Terrabacteria group;p_Tenericutes;c_Mollicutes;o_Mycoplasmatales;f_Mycoplasmataceae;" | 1411 | 415 | 501 | 373 | 56 | 36 | 30 |
| "k_Bacteria;n_Terrabacteria group;p_Actinobacteria;c_Actinobacteria;o_Corynebacteriales;f_Tsukamurellaceae;" | 1384 | 115 | 67 | 102 | 522 | 164 | 414 |
| "k_Bacteria;n_Terrabacteria group;p_Actinobacteria;c_Actinobacteria;o_Corynebacteriales;f_Corynebacteriaceae;" | 1346 | 278 | 268 | 207 | 249 | 165 | 179 |
| "k_Bacteria;n_Terrabacteria group;p_Actinobacteria;c_Coriobacteriia;o_Coriobacteriales;f_Atopobiaceae;" | 1328 | 383 | 394 | 469 | 36 | 23 | 23 |
| "k_Bacteria;n_Terrabacteria group;p_Firmicutes;c_Clostridia;o_Clostridiales;n_Clostridiales incertae sedis;f_Clostridiales Family XIII. Incertae Sedis;" | 1317 | 346 | 505 | 399 | 34 | 24 | 9 |
| "k_Bacteria;p_Proteobacteria;c_Alphaproteobacteria;o_Rhizobiales;f_Brucellaceae;" | 1300 | 322 | 272 | 260 | 160 | 136 | 150 |
| "k_Bacteria;p_Proteobacteria;c_Alphaproteobacteria;o_Pelagibacterales;f_Pelagibacteraceae;" | 1272 | 20 | 57 | 38 | 488 | 425 | 244 |
| "k_Bacteria;n_FCB group;n_Bacteroidetes/Chlorobi group;p_Bacteroidetes;c_Bacteroidia;o_Marinilabiliales;f_Prolixibacteraceae;" | 1261 | 177 | 562 | 304 | 83 | 48 | 87 |
| "k_Bacteria;p_Proteobacteria;c_Oligoflexia;o_Bacteriovoracales;f_Bacteriovoracaceae;" | 1191 | 232 | 143 | 158 | 365 | 196 | 97 |
| "k_Bacteria;p_Proteobacteria;c_Gammaproteobacteria;o_Enterobacterales;f_Hafniaceae;" | 1167 | 338 | 461 | 274 | 42 | 38 | 14 |
| "k_Bacteria;p_Proteobacteria;c_Gammaproteobacteria;o_Cellvibrionales;f_Cellvibrionaceae;" | 1161 | 353 | 159 | 301 | 160 | 114 | 74 |
| "k_Bacteria;n_FCB group;p_Gemmatimonadetes;c_Gemmatimonadetes;o_Gemmatimonadales;f_Gemmatimonadaceae;" | 1150 | 66 | 19 | 22 | 249 | 135 | 659 |
| "k_Bacteria;p_Proteobacteria;c_Betaproteobacteria;o_Nitrosomonadales;f_Gallionellaceae;" | 1136 | 117 | 220 | 160 | 126 | 166 | 347 |
| "k_Bacteria;p_Proteobacteria;c_Gammaproteobacteria;o_Alteromonadales;f_Pseudoalteromonadaceae;" | 1124 | 263 | 349 | 240 | 93 | 71 | 108 |
| "k_Bacteria;n_Terrabacteria group;p_Deinococcus-Thermus;c_Deinococci;o_Deinococcales;f_Deinococcaceae;" | 1033 | 144 | 92 | 51 | 242 | 234 | 270 |
| "k_Bacteria;n_Terrabacteria group;p_Actinobacteria;c_Actinobacteria;o_Streptosporangiales;f_Streptosporangiaceae;" | 1027 | 24 | 27 | 33 | 345 | 240 | 358 |
| "k_Bacteria;n_FCB group;n_Bacteroidetes/Chlorobi group;p_Bacteroidetes;c_Cytophagia;o_Cytophagales;f_Cyclobacteriaceae;" | 1001 | 104 | 147 | 90 | 190 | 193 | 277 |
| "k_Bacteria;n_Terrabacteria group;p_Actinobacteria;c_Actinobacteria;o_Frankiales;f_Frankiaceae;" | 955 | 28 | 35 | 33 | 261 | 267 | 331 |
| "k_Bacteria;p_Proteobacteria;c_Gammaproteobacteria;o_Thiotrichales;f_Thiotrichaceae;" | 939 | 212 | 213 | 161 | 80 | 95 | 178 |
| "k_Bacteria;p_Proteobacteria;c_Gammaproteobacteria;o_Chromatiales;f_Ectothiorhodospiraceae;" | 922 | 107 | 171 | 131 | 133 | 115 | 265 |
| "k_Bacteria;p_Proteobacteria;c_Alphaproteobacteria;o_Holosporales;f_Candidatus Paracaedibacteraceae;" | 884 | 61 | 43 | 71 | 371 | 187 | 151 |
| "k_Bacteria;p_Proteobacteria;c_Hydrogenophilalia;o_Hydrogenophilales;f_Hydrogenophilaceae;" | 877 | 253 | 282 | 263 | 29 | 16 | 34 |
| "k_Bacteria;p_Acidobacteria;c_Acidobacteriia;o_Acidobacteriales;f_Acidobacteriaceae;" | 873 | 38 | 71 | 64 | 255 | 139 | 306 |
| "k_Bacteria;n_PVC group;p_Verrucomicrobia;c_Opitutae;o_Puniceicoccales;f_Puniceicoccaceae;" | 870 | 205 | 182 | 330 | 74 | 49 | 30 |
| "k_Bacteria;n_Terrabacteria group;p_Firmicutes;c_Clostridia;o_Clostridiales;f_Hungateiclostridiaceae;" | 848 | 89 | 145 | 99 | 174 | 195 | 146 |
| "k_Bacteria;n_FCB group;n_Bacteroidetes/Chlorobi group;p_Bacteroidetes;c_Saprospiria;o_Saprospirales;f_Haliscomenobacteraceae;" | 819 | 17 | 16 | 3 | 163 | 55 | 565 |
| "k_Bacteria;n_Terrabacteria group;p_Actinobacteria;c_Actinobacteria;o_Geodermatophilales;f_Geodermatophilaceae;" | 736 | 37 | 27 | 43 | 203 | 150 | 276 |
| "k_Bacteria;p_Proteobacteria;c_Gammaproteobacteria;o_Oceanospirillales;f_Alcanivoracaceae;" | 721 | 40 | 64 | 57 | 223 | 140 | 197 |
| "k_Bacteria;n_Terrabacteria group;p_Actinobacteria;c_Actinobacteria;o_Streptosporangiales;f_Thermomonosporaceae;" | 688 | 18 | 15 | 17 | 224 | 165 | 249 |
| "k_Bacteria;p_Proteobacteria;c_Alphaproteobacteria;o_Holosporales;f_Caedimonadaceae;" | 683 | 19 | 11 | 3 | 198 | 408 | 44 |
| "k_Bacteria;p_Proteobacteria;c_Gammaproteobacteria;o_Thiotrichales;f_Francisellaceae;" | 662 | 123 | 288 | 124 | 44 | 55 | 28 |
| "k_Bacteria;n_Terrabacteria group;p_Deinococcus-Thermus;c_Deinococci;o_Thermales;f_Thermaceae;" | 652 | 26 | 38 | 35 | 141 | 208 | 204 |
| "k_Bacteria;p_Proteobacteria;c_Alphaproteobacteria;o_Rhodobacterales;f_Hyphomonadaceae;" | 650 | 41 | 59 | 61 | 154 | 139 | 196 |
| "k_Bacteria;n_PVC group;p_Planctomycetes;c_Planctomycetia;o_Planctomycetales;f_Isosphaeraceae;" | 627 | 23 | 33 | 37 | 164 | 120 | 250 |
| "k_Bacteria;n_Terrabacteria group;n_Cyanobacteria/Melainabacteria group;p_Cyanobacteria;o_Synechococcales;f_Synechococcaceae;" | 626 | 34 | 50 | 52 | 151 | 145 | 194 |
| "k_Bacteria;n_Terrabacteria group;p_Firmicutes;c_Bacilli;o_Bacillales;f_Listeriaceae;" | 623 | 108 | 210 | 166 | 49 | 49 | 41 |
| "k_Bacteria;p_Acidobacteria;c_Acidobacteriia;o_Bryobacterales;f_Solibacteraceae;" | 620 | 9 | 21 | 18 | 122 | 67 | 383 |
| "k_Bacteria;p_Proteobacteria;c_Gammaproteobacteria;o_Legionellales;f_Coxiellaceae;" | 611 | 35 | 60 | 40 | 213 | 200 | 63 |
| "k_Bacteria;n_Terrabacteria group;p_Firmicutes;c_Tissierellia;o_Tissierellales;f_Tissierellaceae;" | 609 | 155 | 275 | 144 | 16 | 14 | 5 |
| "k_Bacteria;n_Terrabacteria group;n_Cyanobacteria/Melainabacteria group;p_Cyanobacteria;o_Nostocales;f_Nostocaceae;" | 601 | 46 | 62 | 42 | 199 | 117 | 135 |
| "k_Bacteria;n_FCB group;n_Bacteroidetes/Chlorobi group;p_Bacteroidetes;c_Flavobacteriia;o_Flavobacteriales;f_Crocinitomicaceae;" | 589 | 42 | 37 | 46 | 141 | 192 | 131 |
| "k_Bacteria;p_Proteobacteria;c_Gammaproteobacteria;o_Alteromonadales;f_Psychromonadaceae;" | 576 | 151 | 196 | 165 | 43 | 12 | 9 |
| "k_Bacteria;n_PVC group;p_Verrucomicrobia;c_Verrucomicrobiae;o_Verrucomicrobiales;f_Verrucomicrobia subdivision 3;" | 574 | 33 | 42 | 40 | 151 | 56 | 252 |
| "k_Bacteria;n_Terrabacteria group;p_Actinobacteria;c_Actinobacteria;o_Nakamurellales;f_Nakamurellaceae;" | 570 | 192 | 161 | 111 | 52 | 27 | 27 |
| "k_Bacteria;n_Terrabacteria group;p_Actinobacteria;c_Actinobacteria;o_Micrococcales;f_Cellulomonadaceae;" | 556 | 72 | 89 | 110 | 104 | 68 | 113 |
| "k_Bacteria;p_Proteobacteria;n_delta/epsilon subdivisions;c_Deltaproteobacteria;o_Myxococcales;n_Cystobacterineae;f_Anaeromyxobacteraceae;" | 555 | 18 | 21 | 24 | 112 | 65 | 315 |
| "k_Bacteria;p_Aquificae;c_Aquificae;o_Desulfurobacteriales;f_Desulfurobacteriaceae;" | 536 | 153 | 164 | 167 | 27 | 11 | 14 |
| "k_Bacteria;p_Proteobacteria;c_Betaproteobacteria;o_Nitrosomonadales;f_Thiobacillaceae;" | 523 | 36 | 73 | 50 | 67 | 66 | 231 |
| "k_Bacteria;n_FCB group;n_Bacteroidetes/Chlorobi group;p_Chlorobi;c_Chlorobia;o_Chlorobiales;f_Chlorobiaceae;" | 522 | 70 | 110 | 100 | 74 | 73 | 95 |
| "k_Bacteria;n_Terrabacteria group;p_Chloroflexi;c_Chloroflexia;o_Herpetosiphonales;f_Herpetosiphonaceae;" | 521 | 6 | 11 | 8 | 239 | 131 | 126 |
| "k_Bacteria;n_Terrabacteria group;p_Firmicutes;c_Clostridia;o_Thermoanaerobacterales;f_Thermoanaerobacteraceae;" | 517 | 37 | 85 | 66 | 111 | 113 | 105 |
| "k_Bacteria;p_Proteobacteria;n_delta/epsilon subdivisions;c_Deltaproteobacteria;o_Desulfovibrionales;f_Desulfohalobiaceae;" | 499 | 81 | 125 | 51 | 49 | 25 | 168 |
| "k_Bacteria;p_Proteobacteria;c_Alphaproteobacteria;o_Rhizobiales;f_Aurantimonadaceae;" | 483 | 64 | 94 | 62 | 87 | 79 | 97 |
| "k_Bacteria;n_Terrabacteria group;p_Actinobacteria;c_Actinobacteria;o_Micrococcales;f_Brevibacteriaceae;" | 479 | 41 | 49 | 40 | 63 | 110 | 176 |
| "k_Bacteria;n_Terrabacteria group;p_Actinobacteria;c_Actinobacteria;o_Candidatus Nanopelagicales;f_Candidatus Nanopelagicaceae;" | 470 | 4 | 2 | 1 | 96 | 333 | 34 |
| "k_Bacteria;p_Proteobacteria;c_Gammaproteobacteria;o_Thiotrichales;f_Piscirickettsiaceae;" | 438 | 80 | 137 | 93 | 47 | 38 | 43 |
| "k_Bacteria;n_Terrabacteria group;p_Chloroflexi;c_Dehalococcoidia;o_Dehalococcoidales;f_Dehalococcoidaceae;" | 429 | 67 | 92 | 126 | 47 | 40 | 57 |
| "k_Bacteria;n_Terrabacteria group;p_Actinobacteria;c_Thermoleophilia;o_Solirubrobacterales;f_Conexibacteraceae;" | 428 | 20 | 16 | 18 | 138 | 82 | 154 |
| "k_Bacteria;p_Proteobacteria;c_Gammaproteobacteria;o_Enterobacterales;f_Budviciaceae;" | 428 | 165 | 163 | 68 | 14 | 11 | 7 |
| "k_Bacteria;n_PVC group;p_Planctomycetes;c_Candidatus Brocadiae;o_Candidatus Brocadiales;f_Candidatus Brocadiaceae;" | 427 | 19 | 27 | 31 | 92 | 77 | 181 |
| "k_Bacteria;p_Proteobacteria;c_Gammaproteobacteria;o_Cellvibrionales;f_Halieaceae;" | 418 | 39 | 34 | 35 | 107 | 72 | 131 |
| "k_Bacteria;n_Terrabacteria group;p_Actinobacteria;c_Actinobacteria;o_Micrococcales;f_Dermacoccaceae;" | 405 | 31 | 42 | 34 | 126 | 76 | 96 |
| "k_Bacteria;n_Terrabacteria group;p_Firmicutes;c_Bacilli;o_Bacillales;f_Planococcaceae;" | 391 | 134 | 92 | 62 | 42 | 32 | 29 |
| "k_Bacteria;p_Proteobacteria;c_Alphaproteobacteria;o_Rhizobiales;f_Chelatococcaceae;" | 384 | 34 | 48 | 44 | 75 | 53 | 130 |
| "k_Bacteria;p_Proteobacteria;c_Betaproteobacteria;o_Ferrovales;f_Ferrovaceae;" | 379 | 63 | 127 | 76 | 25 | 37 | 51 |
| "k_Bacteria;n_Terrabacteria group;n_Cyanobacteria/Melainabacteria group;p_Cyanobacteria;o_Synechococcales;f_Leptolyngbyaceae;" | 374 | 38 | 19 | 20 | 91 | 75 | 131 |
| "k_Bacteria;n_Terrabacteria group;p_Firmicutes;c_Clostridia;o_Clostridiales;f_Syntrophomonadaceae;" | 369 | 90 | 131 | 87 | 34 | 17 | 10 |
| "k_Bacteria;n_PVC group;p_Chlamydiae;c_Chlamydiia;o_Parachlamydiales;f_Criblamydiaceae;" | 363 | 18 | 9 | 12 | 181 | 70 | 73 |
| "k_Bacteria;p_Proteobacteria;c_Gammaproteobacteria;o_Orbales;f_Orbaceae;" | 363 | 75 | 118 | 92 | 35 | 25 | 18 |
| "k_Bacteria;p_Thermotogae;c_Thermotogae;o_Thermotogales;f_Thermotogaceae;" | 362 | 11 | 20 | 12 | 84 | 196 | 39 |
| "k_Bacteria;p_Proteobacteria;c_Alphaproteobacteria;o_Rickettsiales;f_Anaplasmataceae;" | 358 | 12 | 13 | 9 | 160 | 106 | 58 |
| "k_Bacteria;n_Terrabacteria group;p_Chloroflexi;c_Chloroflexia;o_Chloroflexales;n_Chloroflexineae;f_Oscillochloridaceae;" | 352 | 33 | 8 | 22 | 47 | 30 | 212 |
| "k_Bacteria;n_Terrabacteria group;p_Actinobacteria;c_Actinobacteria;o_Micrococcales;f_Dermatophilaceae;" | 344 | 115 | 82 | 76 | 28 | 19 | 24 |
| "k_Bacteria;p_Spirochaetes;c_Spirochaetia;o_Brachyspirales;f_Brachyspiraceae;" | 339 | 110 | 100 | 91 | 8 | 17 | 13 |
| "k_Bacteria;n_FCB group;n_Bacteroidetes/Chlorobi group;p_Bacteroidetes;c_Cytophagia;o_Cytophagales;f_Flammeovirgaceae;" | 336 | 34 | 69 | 41 | 55 | 54 | 83 |
| "k_Bacteria;p_Proteobacteria;c_Alphaproteobacteria;o_Rhizobiales;f_Bartonellaceae;" | 328 | 12 | 35 | 59 | 91 | 75 | 56 |
| "k_Bacteria;n_Terrabacteria group;p_Firmicutes;c_Bacilli;o_Bacillales;f_Thermoactinomycetaceae;" | 326 | 27 | 11 | 23 | 90 | 46 | 129 |
| "k_Bacteria;n_Terrabacteria group;p_Firmicutes;c_Bacilli;o_Lactobacillales;f_Aerococcaceae;" | 326 | 92 | 115 | 47 | 36 | 21 | 15 |
| "k_Bacteria;n_Terrabacteria group;n_Cyanobacteria/Melainabacteria group;p_Cyanobacteria;n_Oscillatoriophycideae;o_Oscillatoriales;f_Microcoleaceae;" | 322 | 14 | 26 | 19 | 150 | 63 | 50 |
| "k_Bacteria;p_Proteobacteria;c_Alphaproteobacteria;o_Rhizobiales;f_Beijerinckiaceae;" | 320 | 34 | 31 | 42 | 76 | 64 | 73 |
| "k_Bacteria;p_Proteobacteria;c_Gammaproteobacteria;o_Chromatiales;f_Halothiobacillaceae;" | 318 | 35 | 171 | 61 | 18 | 22 | 11 |
| "k_Bacteria;n_Terrabacteria group;p_Chloroflexi;c_Thermoflexia;o_Thermoflexales;f_Thermoflexaceae;" | 306 | 18 | 7 | 12 | 46 | 34 | 189 |
| "k_Bacteria;n_Terrabacteria group;n_Cyanobacteria/Melainabacteria group;p_Cyanobacteria;n_Oscillatoriophycideae;o_Chroococcales;f_Microcystaceae;" | 290 | 25 | 32 | 37 | 36 | 39 | 121 |
| "k_Bacteria;n_FCB group;n_Bacteroidetes/Chlorobi group;p_Bacteroidetes;c_Saprospiria;o_Saprospirales;f_Lewinellaceae;" | 289 | 9 | 11 | 11 | 83 | 36 | 139 |
| "k_Bacteria;p_Proteobacteria;n_delta/epsilon subdivisions;c_Epsilonproteobacteria;o_Campylobacterales;f_Hydrogenimonaceae;" | 288 | 77 | 138 | 57 | 8 | 5 | 3 |
| "k_Bacteria;n_Terrabacteria group;p_Actinobacteria;c_Thermoleophilia;o_Solirubrobacterales;f_Solirubrobacteraceae;" | 285 | 11 | 10 | 11 | 76 | 67 | 110 |
| "k_Bacteria;p_Proteobacteria;c_Gammaproteobacteria;o_Cardiobacteriales;f_Cardiobacteriaceae;" | 283 | 55 | 110 | 72 | 18 | 11 | 17 |
| "k_Bacteria;p_Proteobacteria;c_Oligoflexia;o_Bacteriovoracales;f_Halobacteriovoraceae;" | 283 | 29 | 82 | 55 | 43 | 46 | 28 |
| "k_Bacteria;n_Terrabacteria group;p_Firmicutes;c_Tissierellia;o_Tissierellales;f_Gottschalkiaceae;" | 275 | 60 | 95 | 110 | 4 | 4 | 2 |
| "k_Bacteria;p_Proteobacteria;c_Gammaproteobacteria;o_Thiotrichales;f_Fastidiosibacteraceae;" | 271 | 6 | 7 | 9 | 126 | 101 | 22 |
| "k_Bacteria;n_Terrabacteria group;p_Firmicutes;c_Tissierellia;o_Tissierellales;f_Peptoniphilaceae;" | 270 | 58 | 68 | 74 | 21 | 21 | 28 |
| "k_Bacteria;p_Proteobacteria;c_Gammaproteobacteria;o_Alteromonadales;f_Idiomarinaceae;" | 261 | 59 | 73 | 59 | 28 | 20 | 22 |
| "k_Bacteria;n_Terrabacteria group;p_Actinobacteria;c_Actinobacteria;o_Streptosporangiales;f_Nocardiopsaceae;" | 255 | 7 | 10 | 7 | 85 | 54 | 92 |
| "k_Bacteria;p_Proteobacteria;c_Gammaproteobacteria;o_Oceanospirillales;f_Endozoicomonadaceae;" | 245 | 19 | 29 | 21 | 52 | 57 | 67 |
| "k_Bacteria;n_Terrabacteria group;p_Actinobacteria;c_Actinobacteria;o_Micrococcales;f_Promicromonosporaceae;" | 242 | 23 | 25 | 26 | 78 | 49 | 41 |
| "k_Bacteria;n_Terrabacteria group;p_Firmicutes;c_Clostridia;o_Clostridiales;f_Christensenellaceae;" | 242 | 50 | 68 | 87 | 9 | 21 | 7 |
| "k_Bacteria;p_Proteobacteria;c_Gammaproteobacteria;o_Alteromonadales;f_Colwelliaceae;" | 237 | 54 | 58 | 56 | 23 | 18 | 28 |
| "k_Bacteria;n_Terrabacteria group;p_Armatimonadetes;c_Armatimonadia;o_Capsulimonadales;f_Capsulimonadaceae;" | 233 | 9 | 5 | 3 | 81 | 55 | 80 |
| "k_Bacteria;n_FCB group;n_Bacteroidetes/Chlorobi group;p_Bacteroidetes;c_Bacteroidia;o_Marinilabiliales;f_Marinifilaceae;" | 232 | 29 | 75 | 34 | 36 | 29 | 29 |
| "k_Bacteria;n_Terrabacteria group;p_Actinobacteria;c_Actinobacteria;o_Jiangellales;f_Jiangellaceae;" | 226 | 5 | 6 | 5 | 73 | 33 | 104 |
| "k_Bacteria;p_Acidobacteria;c_Acidobacteriia;o_Bryobacterales;f_Bryobacteraceae;" | 223 | 8 | 2 | 4 | 45 | 22 | 142 |
| "k_Bacteria;p_Proteobacteria;c_Alphaproteobacteria;o_Holosporales;f_Holosporaceae;" | 218 | 0 | 3 | 1 | 111 | 70 | 33 |
| "k_Bacteria;n_PVC group;p_Verrucomicrobia;c_Spartobacteria;o_Chthoniobacterales;f_Chthoniobacteraceae;" | 214 | 10 | 24 | 20 | 45 | 40 | 75 |
| "k_Bacteria;p_Proteobacteria;n_delta/epsilon subdivisions;c_Deltaproteobacteria;o_Syntrophobacterales;f_Syntrophobacteraceae;" | 214 | 36 | 29 | 41 | 25 | 28 | 55 |
| "k_Bacteria;n_Terrabacteria group;p_Actinobacteria;c_Acidimicrobiia;o_Acidimicrobiales;f_Ilumatobacteraceae;" | 212 | 13 | 2 | 17 | 27 | 11 | 142 |
| "k_Bacteria;n_Terrabacteria group;p_Actinobacteria;c_Actinobacteria;o_Kineosporiales;f_Kineosporiaceae;" | 205 | 8 | 9 | 10 | 59 | 33 | 86 |
| "k_Bacteria;n_Terrabacteria group;p_Tenericutes;c_Mollicutes;o_Acholeplasmatales;f_Acholeplasmataceae;" | 204 | 98 | 30 | 38 | 15 | 15 | 8 |
| "k_Bacteria;n_Terrabacteria group;p_Actinobacteria;c_Actinobacteria;o_Micrococcales;f_Dermabacteraceae;" | 200 | 41 | 39 | 44 | 37 | 11 | 28 |
| "k_Bacteria;p_Proteobacteria;c_Gammaproteobacteria;o_Chromatiales;f_Thioalkalispiraceae;" | 194 | 32 | 81 | 32 | 11 | 14 | 24 |
| "k_Bacteria;p_Aquificae;c_Aquificae;o_Aquificales;f_Aquificaceae;" | 192 | 24 | 30 | 37 | 46 | 24 | 31 |
| "k_Bacteria;p_Proteobacteria;c_Oligoflexia;o_Oligoflexales;f_Pseudobacteriovoracaceae;" | 190 | 1 | 8 | 4 | 65 | 31 | 81 |
| "k_Bacteria;n_Terrabacteria group;p_Firmicutes;c_Bacilli;o_Bacillales;f_Alicyclobacillaceae;" | 186 | 16 | 21 | 16 | 63 | 34 | 36 |
| "k_Bacteria;n_PVC group;p_Planctomycetes;c_Phycisphaerae;o_Phycisphaerales;f_Phycisphaeraceae;" | 184 | 5 | 5 | 4 | 59 | 35 | 76 |
| "k_Bacteria;n_FCB group;n_Bacteroidetes/Chlorobi group;p_Bacteroidetes;c_Saprospiria;o_Saprospirales;f_Saprospiraceae;" | 181 | 10 | 2 | 9 | 37 | 22 | 101 |
| "k_Bacteria;n_Terrabacteria group;p_Actinobacteria;c_Thermoleophilia;o_Solirubrobacterales;f_Patulibacteraceae;" | 179 | 9 | 42 | 5 | 30 | 30 | 63 |
| "k_Bacteria;n_Terrabacteria group;n_Cyanobacteria/Melainabacteria group;p_Cyanobacteria;o_Nostocales;f_Tolypothrichaceae;" | 176 | 14 | 6 | 6 | 45 | 24 | 81 |
| "k_Bacteria;n_Terrabacteria group;p_Firmicutes;c_Clostridia;o_Halanaerobiales;f_Halobacteroidaceae;" | 171 | 43 | 54 | 41 | 8 | 16 | 9 |
| "k_Bacteria;n_Terrabacteria group;p_Chloroflexi;c_Ktedonobacteria;o_Ktedonobacterales;f_Ktedonobacteraceae;" | 165 | 9 | 6 | 7 | 59 | 34 | 50 |
| "k_Bacteria;n_Terrabacteria group;n_Cyanobacteria/Melainabacteria group;p_Cyanobacteria;n_Oscillatoriophycideae;o_Oscillatoriales;f_Oscillatoriaceae;" | 164 | 19 | 24 | 17 | 29 | 26 | 49 |
| "k_Bacteria;n_Terrabacteria group;p_Actinobacteria;c_Acidimicrobiia;o_Acidimicrobiales;f_Acidimicrobiaceae;" | 160 | 2 | 0 | 56 | 18 | 16 | 68 |
| "k_Bacteria;n_Terrabacteria group;p_Actinobacteria;c_Actinobacteria;o_Micrococcales;f_Demequinaceae;" | 159 | 16 | 37 | 27 | 31 | 20 | 28 |
| "k_Bacteria;p_Proteobacteria;c_Gammaproteobacteria;o_Acidiferrobacterales;f_Acidiferrobacteraceae;" | 155 | 11 | 13 | 12 | 47 | 29 | 43 |
| "k_Bacteria;p_Proteobacteria;c_Gammaproteobacteria;o_Oceanospirillales;f_Hahellaceae;" | 155 | 26 | 31 | 15 | 45 | 27 | 11 |
| "k_Bacteria;n_Terrabacteria group;p_Firmicutes;c_Clostridia;o_Clostridiales;n_Clostridiales incertae sedis;f_Clostridiales Family XII. Incertae Sedis;" | 154 | 29 | 65 | 46 | 6 | 6 | 2 |
| "k_Bacteria;n_Terrabacteria group;p_Firmicutes;c_Clostridia;o_Halanaerobiales;f_Halanaerobiaceae;" | 154 | 28 | 37 | 38 | 14 | 17 | 20 |
| "k_Bacteria;n_Terrabacteria group;p_Actinobacteria;c_Actinobacteria;o_Micrococcales;f_Ruaniaceae;" | 151 | 48 | 49 | 36 | 4 | 4 | 10 |
| "k_Bacteria;p_Proteobacteria;n_delta/epsilon subdivisions;c_Deltaproteobacteria;o_Desulfarculales;f_Desulfarculaceae;" | 150 | 15 | 34 | 30 | 25 | 14 | 32 |
| "k_Bacteria;p_Proteobacteria;c_Alphaproteobacteria;o_Rhizobiales;f_Rhodobiaceae;" | 146 | 10 | 23 | 20 | 37 | 23 | 33 |
| "k_Bacteria;p_Proteobacteria;n_delta/epsilon subdivisions;c_Deltaproteobacteria;o_Desulfovibrionales;f_Desulfonatronaceae;" | 146 | 24 | 60 | 26 | 8 | 8 | 20 |
| "k_Bacteria;n_Terrabacteria group;n_Cyanobacteria/Melainabacteria group;p_Cyanobacteria;o_Nostocales;f_Rivulariaceae;" | 144 | 7 | 14 | 13 | 39 | 31 | 40 |
| "k_Bacteria;n_Caldiserica/Cryosericota group;p_Candidatus Cryosericota;c_Candidatus Cryosericia;o_Candidatus Cryosericales;f_Candidatus Cryosericaceae;" | 141 | 11 | 17 | 32 | 19 | 15 | 47 |
| "k_Bacteria;n_Terrabacteria group;p_Actinobacteria;c_Actinobacteria;o_Micrococcales;f_Beutenbergiaceae;" | 137 | 30 | 31 | 24 | 15 | 17 | 20 |
| "k_Bacteria;p_Proteobacteria;c_Gammaproteobacteria;o_Cellvibrionales;f_Spongiibacteraceae;" | 137 | 17 | 19 | 12 | 28 | 16 | 45 |
| "k_Bacteria;n_Terrabacteria group;p_Chloroflexi;c_Chloroflexia;o_Chloroflexales;n_Chloroflexineae;f_Chloroflexaceae;" | 134 | 3 | 1 | 5 | 24 | 22 | 79 |
| "k_Bacteria;n_Terrabacteria group;p_Actinobacteria;c_Actinobacteria;o_Corynebacteriales;f_Dietziaceae;" | 133 | 31 | 32 | 24 | 14 | 9 | 23 |
| "k_Bacteria;n_Terrabacteria group;p_Firmicutes;c_Clostridia;o_Thermoanaerobacterales;f_Thermoanaerobacterales Family III. Incertae Sedis;" | 126 | 24 | 24 | 23 | 24 | 19 | 12 |
| "k_Bacteria;n_Terrabacteria group;p_Chloroflexi;c_Thermomicrobia;n_Sphaerobacteridae;o_Sphaerobacterales;n_Sphaerobacterineae;f_Sphaerobacteraceae;" | 120 | 1 | 3 | 6 | 26 | 37 | 47 |
| "k_Bacteria;n_FCB group;n_Bacteroidetes/Chlorobi group;p_Bacteroidetes;o_Bacteroidetes Order II. Incertae sedis;f_Rhodothermaceae;" | 118 | 5 | 12 | 7 | 30 | 20 | 44 |
| "k_Bacteria;n_Terrabacteria group;n_Cyanobacteria/Melainabacteria group;p_Cyanobacteria;o_Synechococcales;f_Pseudanabaenaceae;" | 118 | 11 | 8 | 11 | 28 | 22 | 38 |
| "k_Bacteria;n_unclassified Bacteria;n_Bacteria candidate phyla;n_Candidatus Dependentiae;c_Candidatus Babeliae;o_Candidatus Babeliales;f_Candidatus Babeliaceae;" | 118 | 4 | 3 | 2 | 72 | 25 | 12 |
| "k_Bacteria;p_Aquificae;c_Aquificae;o_Aquificales;f_Hydrogenothermaceae;" | 118 | 17 | 30 | 20 | 18 | 15 | 18 |
| "k_Bacteria;p_Proteobacteria;c_Alphaproteobacteria;o_Magnetococcales;f_Magnetococcaceae;" | 113 | 8 | 15 | 19 | 19 | 20 | 32 |
| "k_Bacteria;p_Proteobacteria;c_Alphaproteobacteria;o_Sneathiellales;f_Sneathiellaceae;" | 113 | 1 | 4 | 7 | 42 | 33 | 26 |
| "k_Bacteria;p_Proteobacteria;n_delta/epsilon subdivisions;c_Epsilonproteobacteria;o_Nautiliales;f_Nautiliaceae;" | 113 | 19 | 39 | 18 | 14 | 8 | 15 |
| "k_Bacteria;p_Thermotogae;c_Thermotogae;o_Petrotogales;f_Petrotogaceae;" | 113 | 15 | 23 | 22 | 17 | 16 | 20 |
| "k_Bacteria;p_Proteobacteria;n_delta/epsilon subdivisions;c_Deltaproteobacteria;o_Desulfurellales;f_Desulfurellaceae;" | 111 | 10 | 61 | 28 | 5 | 4 | 3 |
| "k_Bacteria;n_Terrabacteria group;p_Armatimonadetes;c_Fimbriimonadia;o_Fimbriimonadales;f_Fimbriimonadaceae;" | 108 | 7 | 3 | 0 | 45 | 14 | 39 |
| "k_Bacteria;p_Proteobacteria;c_Alphaproteobacteria;o_Micropepsales;f_Micropepsaceae;" | 106 | 6 | 1 | 1 | 49 | 11 | 38 |
| "k_Bacteria;p_Proteobacteria;c_Gammaproteobacteria;o_Salinisphaerales;f_Salinisphaeraceae;" | 103 | 4 | 13 | 7 | 28 | 15 | 36 |
| "k_Bacteria;p_Proteobacteria;c_Gammaproteobacteria;o_Oceanospirillales;f_Oleiphilaceae;" | 100 | 9 | 11 | 16 | 20 | 25 | 19 |
| "k_Bacteria;n_Terrabacteria group;n_Cyanobacteria/Melainabacteria group;p_Cyanobacteria;o_Nostocales;f_Hapalosiphonaceae;" | 97 | 11 | 11 | 9 | 19 | 13 | 34 |
| "k_Bacteria;n_Terrabacteria group;n_Cyanobacteria/Melainabacteria group;p_Candidatus Margulisbacteria;c_Candidatus Termititenacia;o_Candidatus Termititenacales;f_Candidatus Termititenacaceae;" | 94 | 5 | 17 | 4 | 30 | 30 | 8 |
| "k_Bacteria;n_Terrabacteria group;p_Firmicutes;c_Clostridia;o_Clostridiales;f_Defluviitaleaceae;" | 92 | 7 | 22 | 24 | 10 | 19 | 10 |
| "k_Bacteria;p_Proteobacteria;c_Gammaproteobacteria;o_Alteromonadales;f_Moritellaceae;" | 87 | 12 | 18 | 13 | 5 | 6 | 33 |
| "k_Bacteria;n_FCB group;n_Bacteroidetes/Chlorobi group;p_Bacteroidetes;c_Bacteroidia;o_Bacteroidales;f_Lentimicrobiaceae;" | 86 | 10 | 15 | 21 | 10 | 5 | 25 |
| "k_Bacteria;n_Terrabacteria group;p_Firmicutes;c_Clostridia;o_Clostridiales;f_Heliobacteriaceae;" | 86 | 18 | 19 | 20 | 10 | 11 | 8 |
| "k_Bacteria;n_FCB group;n_Bacteroidetes/Chlorobi group;p_Balneolaeota;c_Balneolia;o_Balneolales;f_Balneolaceae;" | 83 | 3 | 7 | 4 | 18 | 18 | 33 |
| "k_Bacteria;p_Acidobacteria;c_Vicinamibacteria;f_Vicinamibacteraceae;" | 83 | 1 | 3 | 3 | 24 | 6 | 46 |
| "k_Bacteria;n_Terrabacteria group;p_Firmicutes;c_Clostridia;o_Clostridiales;n_Clostridiales incertae sedis;f_Clostridiales Family XVII. Incertae Sedis;" | 80 | 3 | 4 | 5 | 22 | 20 | 26 |
| "k_Bacteria;n_Terrabacteria group;p_Actinobacteria;c_Rubrobacteria;o_Gaiellales;f_Gaiellaceae;" | 79 | 0 | 6 | 2 | 12 | 28 | 31 |
| "k_Bacteria;p_Proteobacteria;c_Gammaproteobacteria;o_Cellvibrionales;f_Microbulbiferaceae;" | 78 | 5 | 11 | 7 | 15 | 16 | 24 |
| "k_Bacteria;p_Elusimicrobia;c_Endomicrobia;o_Endomicrobiales;f_Endomicrobiaceae;" | 77 | 6 | 33 | 24 | 4 | 5 | 5 |
| "k_Bacteria;n_Terrabacteria group;n_Cyanobacteria/Melainabacteria group;p_Cyanobacteria;o_Synechococcales;f_Prochloraceae;" | 73 | 3 | 13 | 2 | 15 | 22 | 18 |
| "k_Bacteria;p_Proteobacteria;c_Alphaproteobacteria;o_Rhizobiales;f_Ancalomicrobiaceae;" | 71 | 6 | 34 | 19 | 1 | 5 | 6 |
| "k_Bacteria;n_Terrabacteria group;p_Actinobacteria;c_Actinobacteria;o_Corynebacteriales;f_Williamsiaceae;" | 70 | 4 | 10 | 9 | 9 | 14 | 24 |
| "k_Bacteria;p_Proteobacteria;c_Gammaproteobacteria;o_Methylococcales;f_Crenotrichaceae;" | 70 | 6 | 14 | 13 | 7 | 7 | 23 |
| "k_Bacteria;n_FCB group;n_Bacteroidetes/Chlorobi group;p_Rhodothermaeota;c_Rhodothermia;o_Rhodothermales;f_Rubricoccaceae;" | 68 | 4 | 6 | 2 | 28 | 6 | 22 |
| "k_Bacteria;p_Proteobacteria;c_Alphaproteobacteria;o_Rhizobiales;f_Cohaesibacteraceae;" | 68 | 6 | 5 | 6 | 21 | 18 | 12 |
| "k_Bacteria;n_Terrabacteria group;n_Cyanobacteria/Melainabacteria group;p_Cyanobacteria;n_Oscillatoriophycideae;o_Chroococcales;f_Aphanothecaceae;" | 67 | 2 | 5 | 7 | 20 | 10 | 23 |
| "k_Bacteria;p_Acidobacteria;c_Blastocatellia;o_Blastocatellales;f_Pyrinomonadaceae;" | 67 | 1 | 2 | 0 | 10 | 6 | 48 |
| "k_Bacteria;p_Calditrichaeota;c_Calditrichae;o_Calditrichales;f_Calditrichaceae;" | 67 | 9 | 4 | 4 | 17 | 12 | 21 |
| "k_Bacteria;p_Proteobacteria;c_Alphaproteobacteria;o_Rhizobiales;f_Roseiarcaceae;" | 66 | 7 | 21 | 7 | 9 | 9 | 13 |
| "k_Bacteria;p_Proteobacteria;c_Gammaproteobacteria;o_Alteromonadales;f_Ferrimonadaceae;" | 66 | 11 | 22 | 16 | 5 | 5 | 7 |
| "k_Bacteria;n_Terrabacteria group;p_Actinobacteria;c_Nitriliruptoria;o_Euzebyales;f_Euzebyaceae;" | 62 | 4 | 1 | 4 | 15 | 16 | 22 |
| "k_Bacteria;p_Thermotogae;c_Thermotogae;o_Kosmotogales;f_Kosmotogaceae;" | 62 | 19 | 12 | 6 | 8 | 7 | 10 |
| "k_Bacteria;n_Terrabacteria group;p_Actinobacteria;c_Actinobacteria;o_Glycomycetales;f_Glycomycetaceae;" | 59 | 0 | 2 | 2 | 18 | 14 | 23 |
| "k_Bacteria;n_Terrabacteria group;n_Cyanobacteria/Melainabacteria group;p_Cyanobacteria;n_Oscillatoriophycideae;o_Oscillatoriales;f_Coleofasciculaceae;" | 58 | 2 | 6 | 5 | 9 | 24 | 12 |
| "k_Bacteria;n_Terrabacteria group;n_Cyanobacteria/Melainabacteria group;p_Cyanobacteria;o_Synechococcales;f_Merismopediaceae;" | 58 | 1 | 3 | 0 | 24 | 16 | 14 |
| "k_Bacteria;n_Terrabacteria group;p_Actinobacteria;c_Nitriliruptoria;o_Egibacterales;f_Egibacteraceae;" | 57 | 1 | 0 | 4 | 9 | 9 | 34 |
| "k_Bacteria;n_PVC group;p_Chlamydiae;c_Chlamydiia;o_Parachlamydiales;f_Simkaniaceae;" | 56 | 2 | 0 | 2 | 30 | 13 | 9 |
| "k_Bacteria;p_Proteobacteria;c_Alphaproteobacteria;o_Kordiimonadales;f_Kordiimonadaceae;" | 56 | 1 | 3 | 2 | 21 | 20 | 9 |
| "k_Bacteria;p_Proteobacteria;c_Gammaproteobacteria;o_Oceanospirillales;f_Saccharospirillaceae;" | 56 | 15 | 6 | 11 | 13 | 2 | 9 |
| "k_Bacteria;n_Terrabacteria group;n_Cyanobacteria/Melainabacteria group;p_Cyanobacteria;n_Oscillatoriophycideae;o_Oscillatoriales;f_Cyanothecaceae;" | 54 | 8 | 6 | 5 | 10 | 12 | 13 |
| "k_Bacteria;n_PVC group;p_Chlamydiae;c_Chlamydiia;o_Parachlamydiales;f_Waddliaceae;" | 52 | 2 | 2 | 0 | 19 | 17 | 12 |
| "k_Bacteria;n_Terrabacteria group;p_Chloroflexi;c_Ktedonobacteria;o_Ktedonobacterales;f_Thermosporotrichaceae;" | 52 | 2 | 1 | 0 | 14 | 20 | 15 |
| "k_Bacteria;n_FCB group;n_Bacteroidetes/Chlorobi group;p_Ignavibacteriae;c_Ignavibacteria;o_Ignavibacteriales;f_Ignavibacteriaceae;" | 51 | 1 | 2 | 1 | 9 | 10 | 28 |
| "k_Bacteria;n_unclassified Bacteria;n_Bacteria candidate phyla;p_Candidatus Riflebacteria;c_Candidatus Ozemobacteria;n_Candidatus Ozemobacterales;f_Candidatus Ozemobacteraceae;" | 51 | 7 | 1 | 9 | 12 | 13 | 9 |
| "k_Bacteria;p_Proteobacteria;c_Acidithiobacillia;o_Acidithiobacillales;f_Thermithiobacillaceae;" | 51 | 1 | 8 | 4 | 15 | 14 | 9 |
| "k_Bacteria;p_Proteobacteria;c_Alphaproteobacteria;o_Rickettsiales;f_Candidatus Midichloriaceae;" | 50 | 0 | 0 | 0 | 22 | 17 | 11 |
| "k_Bacteria;p_Thermotogae;c_Thermotogae;o_Thermotogales;f_Fervidobacteriaceae;" | 50 | 10 | 8 | 9 | 11 | 8 | 4 |
| "k_Bacteria;n_FCB group;n_Bacteroidetes/Chlorobi group;p_Bacteroidetes;c_Flavobacteriia;o_Flavobacteriales;f_Schleiferiaceae;" | 49 | 8 | 7 | 1 | 7 | 6 | 20 |
| "k_Bacteria;n_Terrabacteria group;n_Cyanobacteria/Melainabacteria group;p_Cyanobacteria;o_Nostocales;f_Scytonemataceae;" | 49 | 4 | 2 | 3 | 18 | 12 | 10 |
| "k_Bacteria;p_Proteobacteria;c_Gammaproteobacteria;o_Immundisolibacterales;f_Immundisolibacteraceae;" | 49 | 7 | 1 | 5 | 8 | 6 | 22 |
| "k_Bacteria;p_Thermodesulfobacteria;c_Thermodesulfobacteria;o_Thermodesulfobacteriales;f_Thermodesulfobacteriaceae;" | 48 | 2 | 9 | 6 | 6 | 10 | 15 |
| "k_Bacteria;n_Terrabacteria group;p_Actinobacteria;c_Actinobacteria;o_Actinopolysporales;f_Actinopolysporaceae;" | 47 | 0 | 2 | 1 | 18 | 18 | 8 |
| "k_Bacteria;n_Terrabacteria group;p_Firmicutes;c_Clostridia;o_Clostridiales;f_Catabacteriaceae;" | 46 | 4 | 24 | 13 | 2 | 1 | 2 |
| "k_Bacteria;p_Proteobacteria;c_Alphaproteobacteria;o_Kiloniellales;f_Kiloniellaceae;" | 46 | 3 | 5 | 3 | 11 | 13 | 11 |
| "k_Bacteria;p_Proteobacteria;c_Gammaproteobacteria;o_Cellvibrionales;f_Porticoccaceae;" | 45 | 2 | 4 | 6 | 7 | 5 | 21 |
| "k_Bacteria;p_Proteobacteria;n_delta/epsilon subdivisions;c_Deltaproteobacteria;o_Myxococcales;n_Cystobacterineae;f_Vulgatibacteraceae;" | 45 | 2 | 1 | 1 | 9 | 10 | 22 |
| "k_Bacteria;n_Terrabacteria group;p_Armatimonadetes;c_Chthonomonadetes;o_Chthonomonadales;f_Chthonomonadaceae;" | 44 | 4 | 3 | 2 | 11 | 3 | 21 |
| "k_Bacteria;n_Terrabacteria group;p_Firmicutes;c_Clostridia;o_Thermoanaerobacterales;f_Thermoanaerobacterales Family IV. Incertae Sedis;" | 44 | 1 | 5 | 6 | 8 | 13 | 11 |
| "k_Bacteria;n_Terrabacteria group;p_Actinobacteria;c_Actinobacteria;o_Micrococcales;f_Bogoriellaceae;" | 40 | 2 | 4 | 3 | 8 | 6 | 17 |
| "k_Bacteria;n_Terrabacteria group;p_Firmicutes;c_Bacilli;o_Bacillales;f_Sporolactobacillaceae;" | 40 | 1 | 1 | 10 | 18 | 6 | 4 |
| "k_Bacteria;p_Proteobacteria;c_Alphaproteobacteria;o_Emcibacterales;f_Emcibacteraceae;" | 40 | 5 | 7 | 0 | 13 | 7 | 8 |
| "k_Bacteria;n_Terrabacteria group;n_Cyanobacteria/Melainabacteria group;p_Cyanobacteria;n_Oscillatoriophycideae;o_Chroococcales;f_Chroococcaceae;" | 39 | 1 | 4 | 3 | 14 | 7 | 10 |
| "k_Bacteria;n_Terrabacteria group;p_Actinobacteria;c_Rubrobacteria;o_Rubrobacterales;f_Rubrobacteraceae;" | 39 | 2 | 2 | 3 | 12 | 8 | 12 |
| "k_Bacteria;n_FCB group;n_Bacteroidetes/Chlorobi group;p_Bacteroidetes;c_Cytophagia;o_Cytophagales;f_Amoebophilaceae;" | 38 | 0 | 3 | 1 | 7 | 12 | 15 |
| "k_Bacteria;n_Terrabacteria group;p_Actinobacteria;c_Actinobacteria;o_Catenulisporales;f_Catenulisporaceae;" | 38 | 1 | 3 | 2 | 19 | 9 | 4 |
| "k_Bacteria;p_Proteobacteria;c_Gammaproteobacteria;o_Thiotrichales;f_Thiolinaceae;" | 38 | 14 | 4 | 4 | 7 | 5 | 4 |
| "k_Bacteria;p_Chrysiogenetes;c_Chrysiogenetes;o_Chrysiogenales;f_Chrysiogenaceae;" | 37 | 5 | 15 | 8 | 3 | 2 | 4 |
| "k_Bacteria;n_Terrabacteria group;n_Cyanobacteria/Melainabacteria group;p_Cyanobacteria;c_Gloeobacteria;o_Gloeobacterales;f_Gloeobacteraceae;" | 35 | 3 | 4 | 4 | 9 | 6 | 9 |
| "k_Bacteria;p_Proteobacteria;c_Gammaproteobacteria;o_Nevskiales;f_Algiphilaceae;" | 34 | 1 | 4 | 3 | 8 | 7 | 11 |
| "k_Bacteria;p_Proteobacteria;c_Gammaproteobacteria;o_Oceanospirillales;f_Kangiellaceae;" | 34 | 8 | 6 | 4 | 6 | 2 | 8 |
| "k_Bacteria;p_Spirochaetes;c_Spirochaetia;o_Spirochaetales;f_Borreliaceae;" | 34 | 5 | 3 | 3 | 9 | 7 | 7 |
| "k_Bacteria;n_Nitrospinae/Tectomicrobia group;p_Nitrospinae;c_Nitrospinia;o_Nitrospinales;f_Nitrospinaceae;" | 33 | 2 | 4 | 2 | 8 | 9 | 8 |
| "k_Bacteria;n_Terrabacteria group;p_Tenericutes;c_Mollicutes;o_Anaeroplasmatales;f_Anaeroplasmataceae;" | 33 | 2 | 2 | 3 | 1 | 25 | 0 |
| "k_Bacteria;n_PVC group;p_Verrucomicrobia;c_Verrucomicrobiae;o_Verrucomicrobiales;f_Rubritaleaceae;" | 31 | 1 | 8 | 0 | 8 | 7 | 7 |
| "k_Bacteria;p_Proteobacteria;c_Oligoflexia;o_Silvanigrellales;f_Silvanigrellaceae;" | 30 | 1 | 1 | 1 | 16 | 4 | 7 |
| "k_Bacteria;n_FCB group;n_Bacteroidetes/Chlorobi group;p_Bacteroidetes;c_Cytophagia;o_Cytophagales;f_Microscillaceae;" | 29 | 2 | 5 | 1 | 6 | 5 | 10 |
| "k_Bacteria;n_FCB group;p_Fibrobacteres;c_Chitinispirillia;o_Chitinispirillales;f_Chitinispirillaceae;" | 29 | 2 | 4 | 0 | 13 | 2 | 8 |
| "k_Bacteria;n_Terrabacteria group;p_Actinobacteria;c_Thermoleophilia;o_Thermoleophilales;f_Thermoleophilaceae;" | 29 | 0 | 0 | 1 | 11 | 3 | 14 |
| "k_Bacteria;p_Proteobacteria;c_Zetaproteobacteria;o_Mariprofundales;f_Mariprofundaceae;" | 29 | 1 | 1 | 2 | 12 | 3 | 10 |
| "k_Bacteria;n_PVC group;p_Kiritimatiellaeota;c_Kiritimatiellae;o_Kiritimatiellales;f_Kiritimatiellaceae;" | 28 | 1 | 6 | 2 | 5 | 6 | 8 |
| "k_Bacteria;n_Terrabacteria group;n_Cyanobacteria/Melainabacteria group;p_Cyanobacteria;o_Chroococcidiopsidales;f_Chroococcidiopsidaceae;" | 28 | 1 | 3 | 0 | 12 | 6 | 6 |
| "k_Bacteria;n_Terrabacteria group;p_Actinobacteria;c_Nitriliruptoria;o_Nitriliruptorales;f_Nitriliruptoraceae;" | 28 | 1 | 0 | 4 | 12 | 2 | 9 |
| "k_Bacteria;p_Proteobacteria;c_Gammaproteobacteria;o_Enterobacterales;f_Thorselliaceae;" | 28 | 10 | 5 | 11 | 0 | 2 | 0 |
| "k_Bacteria;n_FCB group;n_Bacteroidetes/Chlorobi group;p_Bacteroidetes;c_Flavobacteriia;o_Flavobacteriales;f_Cryomorphaceae;" | 27 | 2 | 2 | 4 | 4 | 6 | 9 |
| "k_Bacteria;n_FCB group;n_Bacteroidetes/Chlorobi group;p_Bacteroidetes;c_Cytophagia;o_Cytophagales;f_Bernardetiaceae;" | 26 | 2 | 2 | 2 | 5 | 3 | 12 |
| "k_Bacteria;n_Terrabacteria group;n_Cyanobacteria/Melainabacteria group;p_Cyanobacteria;o_Synechococcales;f_Prochlorotrichaceae;" | 26 | 0 | 7 | 3 | 5 | 1 | 10 |
| "k_Bacteria;n_Terrabacteria group;p_Abditibacteriota;c_Abditibacteria;o_Abditibacteriales;f_Abitibacteriaceae;" | 26 | 2 | 1 | 5 | 7 | 5 | 6 |
| "k_Bacteria;p_Proteobacteria;c_Gammaproteobacteria;o_Chromatiales;f_Wenzhouxiangellaceae;" | 26 | 2 | 1 | 4 | 7 | 1 | 11 |
| "k_Bacteria;n_Terrabacteria group;n_Cyanobacteria/Melainabacteria group;p_Cyanobacteria;o_Synechococcales;f_Acaryochloridaceae;" | 25 | 2 | 2 | 1 | 7 | 6 | 7 |
| "k_Bacteria;n_FCB group;n_Bacteroidetes/Chlorobi group;p_Bacteroidetes;c_Bacteroidia;o_Marinilabiliales;f_Salinivirgaceae;" | 24 | 2 | 7 | 2 | 7 | 1 | 5 |
| "k_Bacteria;p_Proteobacteria;n_delta/epsilon subdivisions;c_Deltaproteobacteria;o_Bradymonadales;f_Bradymonadaceae;" | 24 | 1 | 1 | 0 | 6 | 8 | 8 |
| "k_Bacteria;n_Terrabacteria group;p_Actinobacteria;c_Actinobacteria;o_Micrococcales;f_Jonesiaceae;" | 23 | 3 | 1 | 10 | 6 | 3 | 0 |
| "k_Bacteria;n_unclassified Bacteria;n_Bacteria candidate phyla;p_Candidatus Sumerlaeota;c_Candidatus Sumerlaeia;o_Candidatus Sumerlaeales;f_Candidatus Sumerlaeaceae;" | 23 | 1 | 2 | 1 | 7 | 3 | 9 |
| "k_Bacteria;p_Proteobacteria;c_Alphaproteobacteria;o_Rhodospirillales;f_Geminicoccaceae;" | 23 | 8 | 2 | 2 | 0 | 4 | 7 |
| "k_Bacteria;n_FCB group;n_Bacteroidetes/Chlorobi group;p_Bacteroidetes;c_Cytophagia;o_Cytophagales;f_Persicobacteraceae;" | 22 | 1 | 2 | 8 | 3 | 1 | 7 |
| "k_Bacteria;n_FCB group;n_Bacteroidetes/Chlorobi group;p_Ignavibacteriae;c_Ignavibacteria;o_Ignavibacteriales;f_Melioribacteraceae;" | 22 | 1 | 2 | 0 | 11 | 3 | 5 |
| "k_Bacteria;n_FCB group;p_Fibrobacteres;c_Chitinivibrionia;o_Chitinivibrionales;f_Chitinivibrionaceae;" | 22 | 6 | 7 | 5 | 2 | 1 | 1 |
| "k_Bacteria;p_Dictyoglomi;c_Dictyoglomia;o_Dictyoglomales;f_Dictyoglomaceae;" | 22 | 3 | 0 | 3 | 6 | 3 | 7 |
| "k_Bacteria;p_Proteobacteria;c_Alphaproteobacteria;o_Parvularculales;f_Parvularculaceae;" | 22 | 2 | 2 | 1 | 5 | 4 | 8 |
| "k_Bacteria;n_PVC group;p_Planctomycetes;c_Phycisphaerae;o_Sedimentisphaerales;f_Sedimentisphaeraceae;" | 21 | 0 | 2 | 6 | 2 | 2 | 9 |
| "k_Bacteria;n_Terrabacteria group;n_Cyanobacteria/Melainabacteria group;p_Cyanobacteria;o_Pleurocapsales;f_Dermocarpellaceae;" | 21 | 0 | 0 | 1 | 6 | 9 | 5 |
| "k_Bacteria;n_Terrabacteria group;n_Cyanobacteria/Melainabacteria group;p_Cyanobacteria;o_Pleurocapsales;f_Hyellaceae;" | 21 | 4 | 0 | 0 | 5 | 6 | 6 |
| "k_Bacteria;n_Terrabacteria group;p_Actinobacteria;c_Actinobacteria;o_Corynebacteriales;f_Segniliparaceae;" | 21 | 0 | 0 | 0 | 14 | 2 | 5 |
| "k_Bacteria;n_FCB group;n_Bacteroidetes/Chlorobi group;p_Bacteroidetes;c_Cytophagia;o_Cytophagales;f_Thermonemataceae;" | 18 | 1 | 5 | 2 | 6 | 2 | 2 |
| "k_Bacteria;n_PVC group;p_Lentisphaerae;c_Lentisphaeria;o_Lentisphaerales;f_Lentisphaeraceae;" | 18 | 0 | 1 | 3 | 4 | 4 | 6 |
| "k_Bacteria;n_PVC group;p_Verrucomicrobia;c_Verrucomicrobiae;o_Verrucomicrobiales;f_Verrucomicrobia subdivision 6;" | 18 | 0 | 4 | 0 | 3 | 4 | 7 |
| "k_Bacteria;n_Terrabacteria group;n_Cyanobacteria/Melainabacteria group;p_Cyanobacteria;o_Nostocales;f_Chlorogloeopsidaceae;" | 18 | 0 | 1 | 1 | 3 | 5 | 8 |
| "k_Bacteria;n_Terrabacteria group;n_Cyanobacteria/Melainabacteria group;p_Cyanobacteria;o_Synechococcales;f_Chamaesiphonaceae;" | 18 | 0 | 2 | 2 | 8 | 2 | 4 |
| "k_Bacteria;p_Proteobacteria;c_Alphaproteobacteria;o_Rhizobiales;f_Salinarimonadaceae;" | 17 | 4 | 0 | 2 | 2 | 2 | 7 |
| "k_Bacteria;p_Proteobacteria;c_Gammaproteobacteria;o_Methylococcales;f_Methylothermaceae;" | 17 | 0 | 0 | 2 | 7 | 2 | 6 |
| "k_Bacteria;p_Proteobacteria;c_Gammaproteobacteria;o_Oceanospirillales;f_Balneatrichaceae;" | 17 | 4 | 4 | 7 | 1 | 1 | 0 |
| "k_Bacteria;n_FCB group;n_Bacteroidetes/Chlorobi group;p_Bacteroidetes;c_Cytophagia;o_Cytophagales;f_Raineyaceae;" | 16 | 0 | 1 | 1 | 8 | 3 | 3 |
| "k_Bacteria;n_FCB group;p_Candidatus Fermentibacteria;c_Candidatus Fermentibacteria (class);o_Candidatus Fermentibacterales;f_Candidatus Fermentibacteraceae;" | 16 | 3 | 3 | 1 | 6 | 2 | 1 |
| "k_Bacteria;n_Terrabacteria group;p_Actinobacteria;c_Actinobacteria;o_Frankiales;f_Motilibacteraceae;" | 16 | 1 | 3 | 2 | 3 | 2 | 5 |
| "k_Bacteria;n_Caldiserica/Cryosericota group;p_Caldiserica;c_Caldisericia;o_Caldisericales;f_Caldisericaceae;" | 15 | 1 | 2 | 3 | 1 | 4 | 4 |
| "k_Bacteria;n_FCB group;n_Bacteroidetes/Chlorobi group;p_Bacteroidetes;c_Cytophagia;o_Cytophagales;f_Catalimonadaceae;" | 15 | 3 | 2 | 0 | 6 | 3 | 1 |
| "k_Bacteria;p_Coprothermobacterota;c_Coprothermobacteria;o_Coprothermobacterales;f_Coprothermobacteraceae;" | 15 | 2 | 7 | 1 | 2 | 0 | 3 |
| "k_Bacteria;n_FCB group;n_Bacteroidetes/Chlorobi group;p_Chlorobi;c_Chlorobia;o_Chlorobiales;f_Candidatus Thermochlorobacteriaceae;" | 14 | 1 | 2 | 0 | 2 | 2 | 7 |
| "k_Bacteria;n_Terrabacteria group;n_Cyanobacteria/Melainabacteria group;p_Cyanobacteria;o_Spirulinales;f_Spirulinaceae;" | 14 | 0 | 3 | 2 | 0 | 3 | 6 |
| "k_Bacteria;n_Terrabacteria group;p_Chloroflexi;c_Ktedonobacteria;o_Thermogemmatisporales;f_Thermogemmatisporaceae;" | 14 | 0 | 0 | 0 | 4 | 3 | 7 |
| "k_Bacteria;n_Terrabacteria group;p_Firmicutes;c_Bacilli;o_Bacillales;n_Bacillales incertae sedis;f_Bacillales Family X. Incertae Sedis;" | 14 | 1 | 1 | 0 | 6 | 4 | 2 |
| "k_Bacteria;n_Terrabacteria group;p_Firmicutes;c_Clostridia;o_Clostridiales;f_Proteinivoraceae;" | 14 | 8 | 3 | 0 | 3 | 0 | 0 |
| "k_Bacteria;p_Proteobacteria;c_Alphaproteobacteria;o_Rhodothalassiales;f_Rhodothalassiaceae;" | 14 | 0 | 2 | 2 | 5 | 1 | 4 |
| "k_Bacteria;n_PVC group;p_Verrucomicrobia;c_Methylacidiphilae;o_Methylacidiphilales;f_Methylacidiphilaceae;" | 13 | 1 | 0 | 2 | 3 | 1 | 6 |
| "k_Bacteria;n_Terrabacteria group;p_Actinobacteria;c_Actinobacteria;o_Frankiales;f_Sporichthyaceae;" | 13 | 0 | 0 | 0 | 2 | 5 | 6 |
| "k_Bacteria;n_unclassified Bacteria;o_Haloplasmatales;f_Haloplasmataceae;" | 13 | 0 | 6 | 5 | 1 | 1 | 0 |
| "k_Bacteria;n_FCB group;n_Bacteroidetes/Chlorobi group;p_Bacteroidetes;c_Flavobacteriia;o_Flavobacteriales;f_Blattabacteriaceae;" | 12 | 2 | 1 | 1 | 5 | 1 | 2 |
| "k_Bacteria;n_Terrabacteria group;n_Cyanobacteria/Melainabacteria group;p_Cyanobacteria;n_Oscillatoriophycideae;o_Chroococcales;f_Cyanobacteriaceae;" | 12 | 2 | 3 | 0 | 2 | 4 | 1 |
| "k_Bacteria;n_Terrabacteria group;p_Firmicutes;c_Clostridia;o_Clostridiales;f_Caldicoprobacteraceae;" | 12 | 1 | 0 | 1 | 5 | 4 | 1 |
| "k_Bacteria;n_Terrabacteria group;p_Firmicutes;c_Limnochordia;o_Limnochordales;f_Limnochordaceae;" | 12 | 6 | 0 | 1 | 0 | 0 | 5 |
| "k_Bacteria;p_Proteobacteria;c_Gammaproteobacteria;o_Chromatiales;f_Granulosicoccaceae;" | 12 | 1 | 2 | 0 | 2 | 2 | 5 |
| "k_Bacteria;n_Terrabacteria group;n_Cyanobacteria/Melainabacteria group;p_Cyanobacteria;n_Oscillatoriophycideae;o_Oscillatoriales;f_Gomontiellaceae;" | 11 | 0 | 0 | 2 | 8 | 1 | 0 |
| "k_Bacteria;n_Terrabacteria group;n_Cyanobacteria/Melainabacteria group;p_Cyanobacteria;o_Synechococcales;f_Coelosphaeriaceae;" | 11 | 0 | 3 | 1 | 6 | 0 | 1 |
| "k_Bacteria;n_Terrabacteria group;p_Deinococcus-Thermus;c_Deinococci;o_Deinococcales;f_Trueperaceae;" | 11 | 0 | 0 | 2 | 1 | 4 | 4 |
| "k_Bacteria;n_Terrabacteria group;p_Firmicutes;c_Clostridia;o_Candidatus Comantemales;f_Candidatus Comatemaea;" | 11 | 0 | 5 | 3 | 0 | 3 | 0 |
| "k_Bacteria;p_Proteobacteria;c_Alphaproteobacteria;o_Minwuiales;f_Minwuiaceae;" | 11 | 0 | 1 | 1 | 4 | 2 | 3 |
| "k_Bacteria;n_Terrabacteria group;p_Firmicutes;c_Clostridia;o_Clostridiales;f_Gracilibacteraceae;" | 10 | 1 | 2 | 4 | 1 | 0 | 2 |
| "k_Bacteria;n_Terrabacteria group;n_Cyanobacteria/Melainabacteria group;p_Cyanobacteria;o_Pleurocapsales;f_Xenococcaceae;" | 9 | 0 | 0 | 1 | 3 | 1 | 4 |
| "k_Bacteria;n_Terrabacteria group;p_Actinobacteria;c_Actinobacteria;o_Frankiales;f_Cryptosporangiaceae;" | 9 | 0 | 0 | 0 | 3 | 0 | 6 |
| "k_Bacteria;n_Terrabacteria group;p_Actinobacteria;c_Nitriliruptoria;o_Egicoccales;f_Egicoccaceae;" | 9 | 2 | 0 | 0 | 0 | 2 | 5 |
| "k_Bacteria;n_Terrabacteria group;p_Firmicutes;c_Clostridia;o_Clostridiales;n_Clostridiales incertae sedis;f_Clostridiales Family XVI. Incertae Sedis;" | 9 | 1 | 4 | 1 | 2 | 0 | 1 |
| "k_Bacteria;n_Terrabacteria group;p_Firmicutes;c_Clostridia;o_Natranaerobiales;f_Natranaerobiaceae;" | 9 | 4 | 1 | 2 | 0 | 0 | 2 |
| "k_Bacteria;p_Proteobacteria;c_Alphaproteobacteria;o_Rhizobiales;f_Notoacmeibacteraceae;" | 9 | 0 | 1 | 0 | 3 | 4 | 1 |
| "k_Bacteria;p_Proteobacteria;c_Gammaproteobacteria;o_Arenicellales;f_Arenicellaceae;" | 9 | 3 | 2 | 2 | 0 | 0 | 2 |
| "k_Bacteria;p_Thermotogae;c_Thermotogae;o_Mesoaciditogales;f_Mesoaciditogaceae;" | 9 | 3 | 0 | 4 | 1 | 0 | 1 |
| "k_Bacteria;n_FCB group;n_Bacteroidetes/Chlorobi group;p_Rhodothermaeota;c_Rhodothermia;o_Rhodothermales;f_Salinibacteraceae;" | 8 | 0 | 0 | 0 | 2 | 2 | 4 |
| "k_Bacteria;n_Terrabacteria group;p_Firmicutes;c_Clostridia;o_Clostridiales;f_Symbiobacteriaceae;" | 8 | 1 | 0 | 0 | 4 | 2 | 1 |
| "k_Bacteria;p_Acidobacteria;c_Thermoanaerobaculia;o_Thermoanaerobaculales;f_Thermoanaerobaculaceae;" | 8 | 0 | 0 | 0 | 0 | 0 | 8 |
| "k_Bacteria;p_Proteobacteria;c_Gammaproteobacteria;o_Pseudomonadales;f_Ventosimonadaceae;" | 8 | 3 | 3 | 2 | 0 | 0 | 0 |
| "k_Bacteria;p_Proteobacteria;c_Gammaproteobacteria;o_Thiotrichales;f_Thiofilaceae;" | 8 | 1 | 4 | 0 | 0 | 1 | 2 |
| "k_Bacteria;p_Proteobacteria;n_delta/epsilon subdivisions;c_Deltaproteobacteria;f_Candidatus Desulfofervidaceae;" | 8 | 1 | 0 | 1 | 2 | 2 | 2 |
| "k_Bacteria;n_Terrabacteria group;p_Firmicutes;c_Clostridia;o_Thermoanaerobacterales;f_Thermodesulfobiaceae;" | 7 | 3 | 1 | 2 | 0 | 1 | 0 |
| "k_Bacteria;n_Terrabacteria group;p_Tenericutes;c_Mollicutes;o_Entomoplasmatales;f_Entomoplasmataceae;" | 7 | 1 | 2 | 0 | 0 | 4 | 0 |
| "k_Bacteria;p_Proteobacteria;c_Gammaproteobacteria;o_Chromatiales;f_Woeseiaceae;" | 7 | 1 | 0 | 0 | 1 | 0 | 5 |
| "k_Bacteria;n_Terrabacteria group;n_Cyanobacteria/Melainabacteria group;p_Cyanobacteria;n_Oscillatoriophycideae;o_Chroococcales;f_Entophysalidaceae;" | 6 | 0 | 1 | 3 | 1 | 0 | 1 |
| "k_Bacteria;n_Terrabacteria group;p_Chloroflexi;c_Thermomicrobia;o_Thermomicrobiales;f_Thermomicrobiaceae;" | 6 | 2 | 0 | 0 | 1 | 1 | 2 |
| "k_Bacteria;n_Terrabacteria group;p_Tenericutes;c_Mollicutes;o_Entomoplasmatales;f_Spiroplasmataceae;" | 6 | 0 | 2 | 1 | 3 | 0 | 0 |
| "k_Bacteria;p_Proteobacteria;c_Gammaproteobacteria;n_unclassified Gammaproteobacteria;f_Celerinatantimonadaceae;" | 6 | 0 | 2 | 3 | 1 | 0 | 0 |
| "k_Bacteria;n_FCB group;n_Bacteroidetes/Chlorobi group;p_Bacteroidetes;c_Bacteroidia;o_Bacteroidales;f_Balneicellaceae;" | 4 | 1 | 1 | 0 | 1 | 0 | 1 |
| "k_Bacteria;n_PVC group;p_Chlamydiae;c_Chlamydiia;o_Parachlamydiales;f_Candidatus Parilichlamydiaceae;" | 4 | 0 | 0 | 0 | 3 | 1 | 0 |
| "k_Bacteria;n_Terrabacteria group;n_Cyanobacteria/Melainabacteria group;p_Cyanobacteria;n_Oscillatoriophycideae;o_Oscillatoriales;f_Desertifilaceae;" | 4 | 0 | 0 | 0 | 1 | 1 | 2 |
| "k_Bacteria;n_Terrabacteria group;n_Cyanobacteria/Melainabacteria group;p_Cyanobacteria;o_Gloeoemargaritales;f_Gloeomargaritaceae;" | 4 | 0 | 0 | 0 | 0 | 3 | 1 |
| "k_Bacteria;n_Terrabacteria group;n_Cyanobacteria/Melainabacteria group;p_Cyanobacteria;o_Pleurocapsales;f_Hydrococcaceae;" | 4 | 1 | 0 | 1 | 1 | 1 | 0 |
| "k_Bacteria;n_Terrabacteria group;p_Actinobacteria;c_Actinobacteria;o_Catenulisporales;f_Actinospicaceae;" | 4 | 1 | 0 | 0 | 2 | 0 | 1 |
| "k_Bacteria;n_Terrabacteria group;n_Cyanobacteria/Melainabacteria group;p_Cyanobacteria;o_Nostocales;f_Fortieaceae;" | 3 | 0 | 0 | 0 | 3 | 0 | 0 |
| "k_Bacteria;n_Terrabacteria group;p_Actinobacteria;c_Actinobacteria;o_Acidothermales;f_Acidothermaceae;" | 3 | 2 | 0 | 0 | 0 | 0 | 1 |
| "k_Bacteria;p_Proteobacteria;c_Gammaproteobacteria;o_Chromatiales;f_Thioalkalibacteraceae;" | 3 | 0 | 1 | 0 | 0 | 0 | 2 |
| "k_Bacteria;n_Terrabacteria group;n_Cyanobacteria/Melainabacteria group;p_Cyanobacteria;o_Nostocales;f_Symphyonemataceae;" | 2 | 0 | 0 | 0 | 1 | 1 | 0 |
| "k_Bacteria;p_Spirochaetes;c_Spirochaetia;o_Brevinematales;f_Brevinemataceae;" | 2 | 1 | 0 | 0 | 0 | 1 | 0 |
| "k_Bacteria;n_PVC group;p_Chlamydiae;c_Chlamydiia;o_Parachlamydiales;f_Rhabdochlamydiaceae;" | 1 | 0 | 0 | 0 | 0 | 0 | 1 |
| "k_Bacteria;n_Terrabacteria group;p_Actinobacteria;c_Actinobacteria;n_Candidatus Actinomarinidae;o_Candidatus Actinomarinales;n_Candidatus Actinomarineae;f_Candidatus Actinomarinaceae;" | 1 | 0 | 1 | 0 | 0 | 0 | 0 |
| **Total** | **13092319** | **3789664** | **4425540** | **3473630** | **404004** | **365722** | **633759** |

**Table S8. Data of the characteristics of water before (RAW) and after treatment (UV)**

| Parameter | Sampling date | | | | | | | | |
| --- | --- | --- | --- | --- | --- | --- | --- | --- | --- |
|  | Sample 1  June 16, 2015 | | | Sample 2  July 14, 2015 | | | Sample 3  September 15,2015 | | |
|  | RAW | UV | RAW | | UV | RAW | | UV |  |
| pH | 7.30 | 6.52 | 7.65 | | 6.82 | 7.73 | | 6.42 |  |
| Temperature (ºC) | 22.4 | 22.3 | 20.3 | | 26.0 | 22.4 | | 24.6 |  |
| Conductivity (ms/cm, 25ºC) | 904 | 508 | 925 | | 662 | 1128 | | 611 |  |
| DOC (mg O_2_/L) | 813 | <50 | 932 | | <50 | 887 | | <50 |  |
| BOD5 (mg O_2_/L) | 480 | 3 | 510 | | 10 | 550 | | 4 |  |
| TSS (mg/L) | 268 | <10 | 478 | | <10 | 568 | | <10 |  |
| Turbidity (NTU) | n.a. | 2.3 | n.a. | | 3.4 | n.a. | | 2.7 |  |
| Total N (mg/L) | n.a. | n.a. | 90 | | 18 | n.a. | | n.a. |  |
| N-NH_4_ (mg /L) | n.a. | <5.0 | 61 | | 8.4 | n.a. | | 6.9 |  |
| NO_3_ (mg /L) | n.a. | 10.0 | 0.6 | | 7.0 | n.a. | | 10.0 |  |
| Total P (mg/L) | n.a. | n.a. | 12.7 | | 9.7 | n.a. | | n.a. |  |

RAW, wastewater influent; UV, final effluent after secondary treatment and UV disinfection.

DOC: Dissolved organic carbon

BOD5: 5 day biochemical oxygen demand

TSS: Total suspended solids

n.a. Information not available

**Table S9. Amount of DNA per ml extracted from each sample and number of total reads.**

| **Sample** | **DNA (ng/ml)*** | **Number of reads** |
| --- | --- | --- |
| **RAW1** | 182.40 | 6299084 |
| **RAW2** | 165.60 | 8172260 |
| **RAW3** | 161.60 | 6530310 |
| **UVI1** | 31.50 | 6783646 |
| **UVI2** | 31.47 | 6066088 |
| **UVI3** | 29.47 | 6842806 |

*ng of DNA extracted from each ml of sample.
